# Toward evaluating the effect of technology choices on linkages between sustainable development goals

**DOI:** 10.1016/j.isci.2022.105727

**Published:** 2022-12-21

**Authors:** Magdalena M. Klemun, Sanna Ojanperä, Amy Schweikert

**Affiliations:** 1 Division of Public Policy and Energy Institute, The Hong Kong University of Science and Technology, Clear Water Bay, Hong Kong; 2 Oxford Internet Institute, University of Oxford, Oxford, UK; 3 Department of Mechanical Engineering, Colorado School of Mines, Golden, CO, USA; 4 Institute for Data, Systems and Society, Massachusetts Institute of Technology, Cambridge, MA, USA; 5 The Alan Turing Institute, London, UK

**Keywords:** Energy engineering, Energy flexibility, Energy policy, Energy resources, Energy systems

## Abstract

Linkages between the Sustainable Development Goals (SDGs) have sparked research interest because a better understanding of SDG co-benefits may enable faster progress on multiple sustainability fronts. However, SDG linkages are typically analyzed without considering the technologies used to implement a primary SDG, which may have secondary effects on other SDGs. Here, we outline an approach to study this problem by connecting the industries and services required to produce a technology to the United Nations SDG indicator framework, using SDG7 and four energy technologies as an illustrative case. We find that all technologies in our set involve potential co-benefits with SDGs 1, 8–10, 12–13, and 17, and trade-offs with SDGs 6, 8–9, 11–12, and 14–15. Deployment services primarily induce co-benefits; manufacturing has mixed impacts. Our work sheds light on the technology characteristics (e.g., scale, high- or low-tech) that influence linkages while also pointing to SDG-relevant characteristics not captured by UN indicators.

## Highlights

• New method for understanding how technologies link sustainable development goals
• Applied to energy technologies to identify reasons for linkages between the SDGs
• Installation services equally decisive for linkages as manufacturing industries
• Bottom-up view helps identify R&D and policy efforts to strengthen SDG synergies

## Graphical abstract

**Figure:**
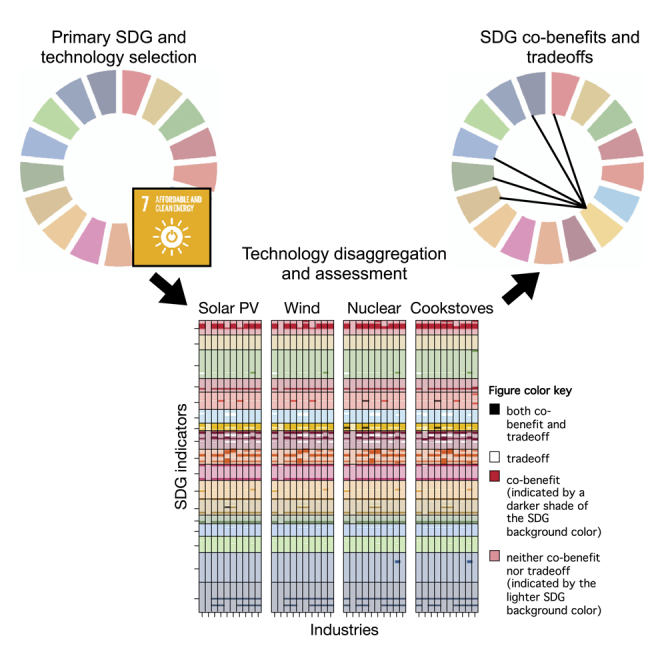

## Introduction

The Sustainable Development Goals (SDGs) are a set of 17 goals and 169 associated targets designed to address the major areas of human development—energy and water access, food security, climate change, and economic development. Following the Millennium Development Goals (2000–2015), the SDGs are meant to represent a more integrated set of priorities that address diverse needs in all regions of the world.^1^^,^^2^^,^^3^ In addition to this high-level framework, 231 official development indicators were assigned to one or more SDG targets to support measurement of SDG-related progress.^4^

Strategies to achieve one specific SDG can affect multiple dimensions of human development, potentially leading to linkages between SDGs. For example, promoting clean energy (SDG7) may involve using new technologies for electricity generation and transportation, and the environmental and economic impacts of technology manufacturing and use may affect indicators related to human health (SDG3), clean water (SDG6), infrastructure development (SDG9), and climate change mitigation and adaptation (SDG13). Understanding such linkages is important to design policies and other efforts that leverage SDG synergies while avoiding trade-offs.

Even prior to the focus on SDG interactions in recent literature, studies have examined co-benefits of environmental policies for other dimensions of human development. Policies to reduce greenhouse gas (GHG) emissions have been linked to better air quality and associated public health improvements, as well as to benefits for ecosystem quality, employment, resource efficiency, and energy and food security (e.g.,^5^^,^^6^^,^^7^^,^^8^). Conversely, air pollution-focused policies have been shown to yield co-benefits for GHG mitigation (e.g.,^9^^,^^10^^,^^11^). Climate policies supporting energy efficiency have been identified as those most likely to achieve synergies across multiple SDG-related targets.^12^ However, much focus in this literature has been placed on a limited set of co-benefits and on developed rather than developing economies.^5^^,^^13^ Moreover, studies on policy co-benefits often use integrated assessment models to evaluate the relationships between changes in geophysical and socio-economic systems. Due to their complexity, these models employ coarse-grained representations of individual technologies and are thus not ideally suited to analyze the impact of technology characteristics on SDG linkages.

A number of recent studies have analyzed SDG linkages using the SDG indicator framework.^2^^,^^14^^,^^15^^,^^16^^,^^17^^,^^18^^,^^19^^,^^20^^,^^21^^,^^22^^,^^23^ These studies differ in how they define SDG linkages, in the analytical methods deployed, and in the specific policy challenges studied.^24^ Studies using expert elicitation have identified SDG7 (“Affordable and Clean Energy”) as highly synergistic with other SDGs, particularly with Goals 8 (economic growth) and 13 (climate change).^14^^,^^15^^,^^25^ Trade-offs between SDG7 and other goals have been found to arise from increases in energy costs due to clean energy expansion^14^ and from increased land competition if SDG7 is pursued using large-scale bioenergy production.^26^ Additionally, the tension between rapid energy infrastructure expansions (e.g., to promote economic growth) poses challenges including the advanced planning required to ensure new infrastructure is suitable for renewable energy integration.^15^ Studies using network analysis have confirmed the central role of SDG7 (along with goals 3, 6, and 9) in the attainment of many other SDGs.^2^ Overall, most literature has pointed toward co-benefits between SDG7 and other goals, though many interactions are context-dependent (i.e., location- or sector-dependent) and warrant further analysis to probe their generalizability.^26^

A small set of studies has probed the reasons for SDG interactions to better understand why and how strongly SDGs interact, and how these interactions can be influenced by policy. These studies have shown, for example, that the way SDGs interact can differ by country income level.^17^ While low-income countries may be able to meet many SDGs without trade-offs due to a lack of existing infrastructure and the comparatively smaller role of technological lock-in, actions to mitigate climate change present barriers to meeting other SDGs in high-income countries.^17^ Shared among these prior studies, however, is the type of information provided on drivers of SDG linkages, which is abstracted from technology-specific characteristics. Reasons cited for SDG linkages are often common knowledge (e.g., the electricity demand of hospitals as a reason for linkages between SDGs 7 and 3) without explicit connections to the SDG indicator framework.

Despite over 90% of possible SDG interactions having been analyzed,^27^ however, no studies exist that investigate how linkages between SDGs are shaped by the technologies chosen to pursue an SDG (e.g., zero-carbon energy technologies supported in pursuit of SDG7, or manufacturing equipment purchased in support of SDG9). Measures to promote energy access and industrial development will often support the manufacturing and deployment of technologies, and the distinct socio-economic and environmental impacts of these technologies may affect how SDGs interact. The recognition that SDG linkages may depend on *how (e.g., with which technology or technology portfolio)*, not just *whether* SDGs are pursued is largely absent from previous work. Several studies have asserted that clean energy technologies such as solar PV or wind may yield benefits for other SDGs, but these studies either do not consider SDG indicators or only consider technology-use phases and omit manufacturing and construction-phase impacts.^28^^,^^29^^,^^30^ Without drawing a connection between technology characteristics and SDG indicators, however, understanding the mechanisms underlying SDG linkages is difficult. Technology-specific impacts may be implicitly considered in expert assessments (e.g.,^3^^,^^15^); but without knowledge of experts’ evaluation methods, it is not possible to know whether and how technologies were analyzed. In addition to prior work on policy co-benefits and SDG linkages, the industrial ecology literature has also contributed to technology evaluation along multiple sustainability dimensions. Comparative life cycle assessments of low-carbon technologies have shown, for example, that wind turbines have lower ecotoxicity and metals depletion impacts per unit of electricity produced compared to polysilicon solar PV, although the greenhouse gas emissions impacts of these technologies are very similar.^31^^,^^32^ Some impact categories used in life cycle analysis (e.g., particulate matter and climate change) can be mapped onto SDG indicators (e.g., particulate matter under SDG11 and ${CO}_{2}$ emissions under SDG9), but others (e.g., ecotoxicity and resource depletion) are difficult to disaggregate. Overall, life cycle assessment impact categories cover metrics related to SDGs 6–9, 12, and 14, but not to SDGs 1–5, 13, and 15–17.

In addition to the academic literature, several tools exist to help evaluate SDG progress at the country level, including the SDG Index and Dashboard^33^ and the Country Development Diagnostics Framework.^34^ While individual tools have been designed to enable assessments of specific implementation strategies,^2^ they do not consider specific technologies and their characteristics (e.g., industry-level impacts).

This paper begins to fill this gap by presenting a bottom-up approach for studying the potential of technologies to induce linkages between SDGs. We use SDG7 as an example and analyze linkages between technology investments in pursuit of SDG7 and non-energy SDGs as captured by the official set of SDG indicators. We consider a set of example energy technologies (photovoltaics, nuclear fission power plants, wind turbines, and clean cookstoves) and present a blueprint analysis of potential linkages between technology-related industries and services and SDG indicators. Our results add new insight on the drivers of SDG linkages and reveal technology characteristics that shape SDG linkages, as well as characteristics not captured by the current indicator framework despite their potential importance for sustainability targets. For the set of technologies analyzed here, linkages between SDG7 and other SDGs are influenced by diverse factors such as technologies’ unit and industry scale, requirement for low- and high-tech industries, and water and waste intensities of manufacturing industries and services. We observe the largest number of potentially co-beneficial linkages between SDG7 and SDGs 1, 8, 9, and 10, and the largest number of potential trade-off linkages between SDG7 and SDGs 6, 8, 12, and 14.

Overall, we find that the prevalence of manufacturing compared to service industries in a technology’s component supply network is a key factor in shaping SDG linkages, due to shared characteristics within these two industry groups. Service industries show potential primarily for inducing co-benefits between SDG7 and 11 other SDGs, while manufacturing industries induce both co-benefits and trade-offs. Regardless of the choice of technology, linkages are highly sensitive to indicator choices for about a third of the 16 non-energy SDGs, suggesting that linkages for these SDGs depend more strongly on how SDGs are interpreted than for others.

## Results

### General framework

**Figure 1:**
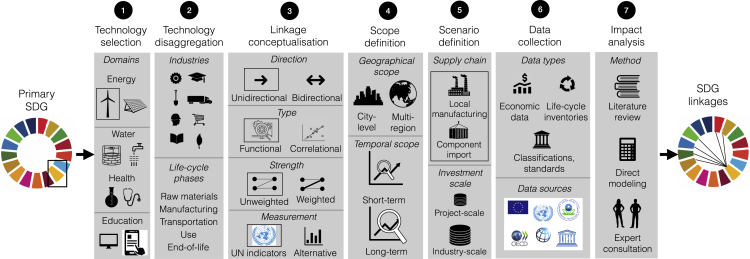
Framework for analyzing SDG linkages The approach developed here follows seven steps: 1) selecting a technology to support progress toward a primary SDG; 2) disaggregating the technology into categories whose impacts on SDG indicators can be studied (e.g., industries, life cycle phases, etc.); 3) conceptualizing linkages in terms of their direction, functional or correlational nature, and weight; 4) defining the scope of the analysis (location, time frame over which linkage is analyzed); 5) defining the scenario; 6) collecting qualitative and quantitative data; and 7) analyzing industry impacts under the assumptions made in steps 1–5. Boxes mark options selected in this study, which focuses on energy technologies such as wind turbines and disaggregates technologies by industries. Linkages are conceptualized as unidirectional (i.e., feedback of energy technology manufacturing and deployment on actions in pursuit of SDG7 are not considered) and functional. Functional linkages are those arising from industries and services strictly required to deliver a technology. This definition excludes secondary effects (e.g., effects of additional employment on sustainable consumption behavior) or effects that may correlate with technology manufacturing and deployment.

We develop a framework in which linkages between a primary SDG and other SDGs are considered possible when the life cycle of a technology (including its design, manufacturing, deployment, use, and end-of-life management) used in pursuit of one SDG shows potential to directly affect one or more indicators of other SDGs. For example, investing in the local manufacturing and deployment of solar photovoltaic (PV) systems to achieve SDG7 is likely to also affect indicators of SDG9 due to the manufacturing and high-tech industry value added (indicators 9.2.1, 9. b.1, see Table S4 for a full list of indicators), and due to additional employment (9.2.2) and possibly R&D expenditure (9.5.1). As shown in Figure 1, assessing a technology’s potential to induce SDG linkages involves, among other steps, the disaggregation of technologies into industries or other categories whose impacts on SDG indicators can be evaluated, a basic description of the type of linkages considered in terms of orientation, causality, and weight (“linkage conceptualization”), and the definition of scenarios for technology component supply. In this paper, each linkage is meant to represent a functional, unidirectional (from SDG7 to other SDGs) connection at the indicator level. We take one connection between an SDG7 indicator and a non-SDG7 indicator as a sufficient condition for a potential linkage between the two SDGs. We do not consider the strength of the connection (e.g., due to small- vs. large-scale investments in a technology, or due to connections to a few vs. many indicators of a non-energy SDG), nor its temporal evolution (short-term vs. long-term changes in SDG indicators). As we discuss in Conclusions, future work could build on our paper to refine the blueprint method developed here.

In our framework, technologies are represented through the industries and services required to manufacture each technology’s physical components (e.g., modules and inverters for PV systems) and the raw materials required for these components. We also include the industries and services used to deploy, operate, and decommission the technology. Industries are differentiated based on the NAICS code system (see STAR methods technology representation for details).

SDGs are represented through the development indicators included in the official UN framework to track countries’ progress on the SDGs. This framework includes 231 official indicators, each assigned to a target associated with progress toward a specific SDG.^35^ To evaluate linkages between SDGs using the UN’s indicator framework, we construct technology-specific matrices where the rows represent technology industries and the columns SDG indicators. As we discuss in section measures to enhance co-benefit and weaken trade-off linkages, future work could expand the set of indicators considered beyond UN guidelines to include more technology-focused metrics, but in this initial work we focus on the UN framework only as it is the currently accepted one. STAR methods section evaluation of SDG linkages gives details on how we evaluate co-beneficial and trade-off linkages.

**Figure 2:**
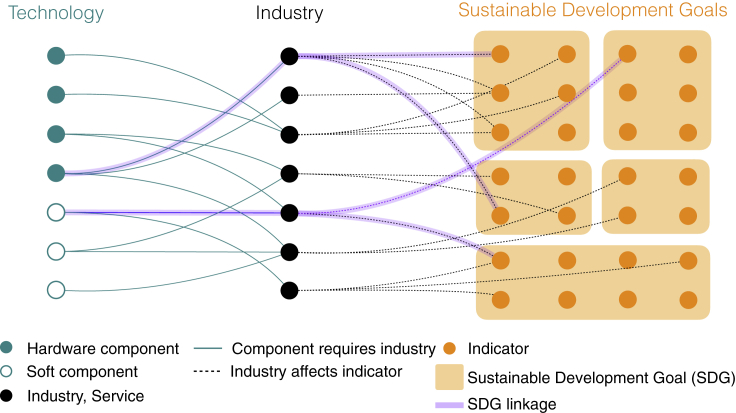
Conceptual representation of technology-induced linkages between the SDGs Technologies consist of hardware components (e.g., photovoltaic panels) and soft components (e.g., installation steps) and are connected to the Sustainable Development Goals through the industries and services needed to manufacture, deploy, operate, and manage the end-of-life of these components (black circles). Progress toward SDGs (orange squares) is measured using SDG indicators (orange circles). Two SDGs are linked (purple lines) when technologies deployed to make progress toward one SDG require components supplied by industries that affect indicators of another SDG.

The relationships between technologies, industries, and SDGs can be understood as a tripartite network connecting technology components and deployment services to SDG indicators (Figure 2). The characteristics of this network are specific to the technology and the SDGs between which linkages are analyzed. Technologies can require a smaller or larger number of manufacturing and deployment steps, and these steps in turn can be completed by a more uniform or diverse set of industries. Similarly, SDGs can differ in terms of the number of indicators. Regardless of the details of the network, a feature of our framework is that SDG linkages are conceptualized in terms of specific mechanisms—technology components requiring manufacturing industries and deployment services to deliver energy services. The non-energy impacts of these industries and deployment services create linkages between SDG 7 and other SDGs.

### Technologies

**Figure 3:**
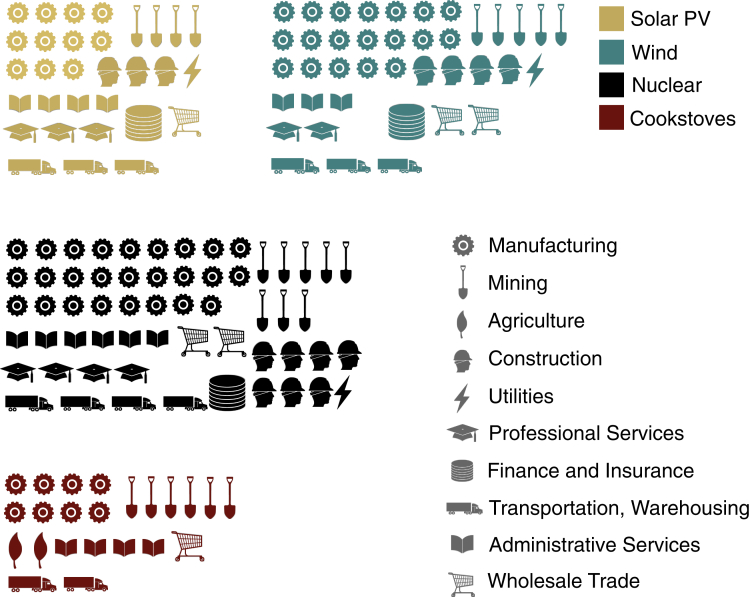
Energy technology industries and services for solar PV, wind turbines, nuclear fission power plants, and clean cookstoves Industries are classified according to 2-digit NAICS codes (names are abbreviated; see Figure 6 for full NAICS category names). Note that for visual simplicity, we use one symbol each to represent two different categories of manufacturing (2-digit NAICS codes 32 and 33) and transportation/warehousing (2-digit NAICS codes 48 and 49), respectively. The number of industries a technology draws on based on two-digit NAICS codes can therefore differ from the number of symbols above, which represents industry sectors within 2-digit NAICS codes.

As shown in Figure 3, the PV component supply network includes 31 industries at the 6-digit NAICS code level (see supplementary information Table S9). The industries in the PV network fall into nine different industry sectors and eleven different industries at the 2-digit NAICS level. The largest number of PV components are supplied by manufacturing industries (11 out of 31 industries), including, for example, manufacturing of solar cells, flat glass, and circuit boards used in solar panels and inverters, and refining and smelting aluminum used in PV mounting rails. The other 20 industries are evenly distributed across mining, construction, transportation & warehousing, professional services, finance, and wholesale trade.

The wind turbine component supply network contains 40 industries at the 6-digit NAICS code level (Figure 3, supplementary information Tables S10 and S11), approximately half of which are manufacturing industries. Compared to PV, the larger number of industries can be explained by additional and separately specialized materials required for turbine, blade, and tower manufacturing, including fabricated structural metals for the tower and synthetic fibers for the blades. In contrast to wind turbines, the core electricity-generating unit for solar PV—the solar cell—is supplied by one single industry (“Semiconductor and related device manufacturing”).

The nuclear fission plant network includes 59 industries (supplementary information Tables S12 and S13), almost twice as many industries at the 6-digit level as the PV network. Nearly half of these industries are classified as “Manufacturing” at the two-digit NAICS level, followed by “Mining” and “Construction”/“Contractors” with eight and seven industries each. Overall, despite the differences observed in the overall number of industries, PV, wind, and nuclear draw on the same number of industry sectors (9 industry sectors each, represented by nine symbols in the respective technology sub-graphics in Figure 3).

Finally, the clean cookstove network is the “simplest” network, both in terms of components and industries (supplementary information Table S15). It includes 23 industries at the 6-digit NAICS code level. As the only required step outside the stove factory is stove shipping and selling, two-thirds of the cookstove industries are either mining or manufacturing industries. Two industries fall under “Agriculture” (due to the required supply of solid fuel).

### SDG linkages

For the scenarios considered, all energy technologies show potential to induce both co-benefits and trade-offs between SDG7 and six non-energy SDGs (5, 6, 8, 9, 11, and 12). For other SDGs, the effects are primarily positive (SDGs 1, 4, 10, 13, and 17) or primarily negative (3, 14, and 15). Although there are some differences across technologies, the differences in SDG7-synergies and trade-offs across SDGs for the same technology are generally larger than across technologies for the same SDG. Note that this result does not account for the strength of individual linkages, which may lead to smaller or larger differences across technologies (see limitations of the study).

Linkages between SDG7 and other SDGs are influenced by diverse factors such as technologies’ unit and industry scale, requirement for high- and low-tech industries and informal labor, industry wage levels, water and waste intensities of manufacturing processes, and technology industries' and services gender distribution in managerial positions. Overall, we find that the potential to create linkages between SDG7 and non-energy SDGs differs between deployment strategies, and that the number of industries influencing this potential differs across SDGs. For some non-energy SDGs (SDG 1, 8, 9, and 17), linkages to SDG7 are driven by just a few industries, while for other SDGs, the potential to induce linkages is spread across almost all technology industries and services.

For the majority of SDGs, technology industries show potential to influence multiple indicators. However, for SDGs related to health, education, sustainable cities, climate change, and institutions (SDGs 3, 4, 5, 11, 13, and 17), linkages are driven by only one or two indicators. As a result, whether or not these linkages are perceived as important will be more sensitive to how SDGs are interpreted in various practical contexts. For example, emphasizing SDG4 (“Quality Education”) indicators related to electricity access for schools will suggest stronger linkages between SDG7 and SDG4 if small-scale low-carbon technologies are deployed at schools, while a focus on SDG4 indicators related to global citizenship and sustainable development education would suggest a weaker connection between SDG4 and SDG7. Similarly, emphasizing SDG11 (“Sustainable Cities”) indicators related to air pollution would suggest that potential linkages between SDG7 and SDG11 exist, while a focus on indicators related to easy transportation access and participatory urban planning would not.

We also observe that the prevalence of manufacturing compared to service industries in a technology’s network is a key factor in determining co-benefits and trade-offs between SDG7 and other SDGs. Service industries show potential primarily for inducing co-benefits between SDG7 and 11 other SDGs, while manufacturing industries induce both co-benefits and trade-offs.

Comparing technology-specific results, the network of PV’s industries and services includes a larger share of industries with potential to create or strengthen co-benefit linkages (corresponding to a higher linkage density, see STAR Methods evaluation of SDG linkages and Equation 2 therein) compared to other technologies. Co-benefits arise from, for example, the potential impacts of PV expansion on R&D-intensive electrical component and semiconductor industries (SDG9), informal employment in specialty contractor and construction companies (SDG8), and public-private partnerships in PV project delivery (SDG17). (We note that indicator 8.3.1, Proportion of Informal Employment in Non-Agricultural Employment is one of the indicators on which countries may have different perspectives depending on their stage of economic development. Developing economies with high rates of unemployment may consider growing informal employment as a positive change, while developed economies may not.)

#### Component import scenario

**Figure 4:**
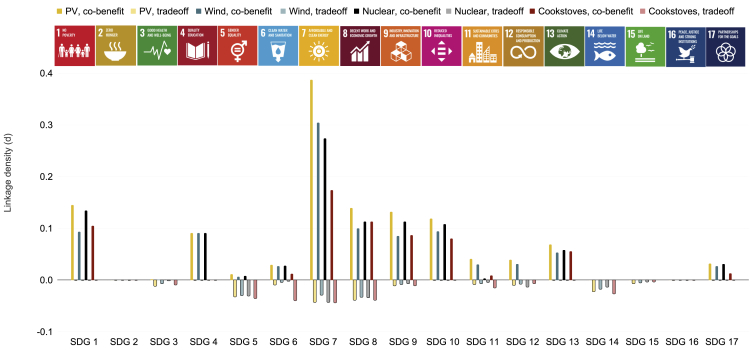
SDG indicator densities for the component import scenario Dark bars indicate co-benefit densities and light bars indicate trade-off densities (see Equations 1 and 2). A higher density means that a larger fraction of possible connections between technology industries and services and SDG indicators is supported by evidence in the literature reviewed for this study. Linkage density is not, however, a direct measure of linkage strength, as one single industry may have larger impacts on a particular indicator than several ones (see section limitations of the study for a discussion of this issue).

**Figure 5:**
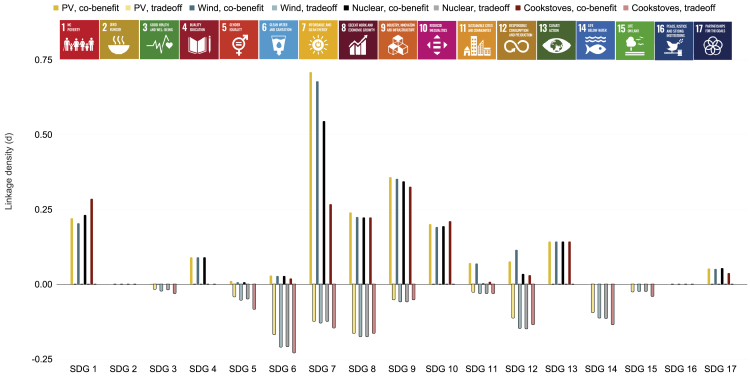
Linkage densities for the local manufacturing scenario Dark bars indicate co-benefit densities and light bars indicate trade-off densities (see Equations 1 and 2).

As shown in Figures 4 and 5, indicator densities differ somewhat across technologies, but the differences are small. PV ranks as the technology with the highest density of potential co-benefit linkages between SDG7 and non-energy SDGs, and clean cookstoves exhibit the lowest densities. For SDG1, PV shows higher potential for co-benefits due to a larger share of simple, non-high-tech industries required for PV deployment. These industries, including electrical contractors, site preparation contractors, power line construction contractors, and freight transportation, are more likely to create jobs for people living in low-income households as compared to high-tech manufacturing industries, thereby influencing indicators related to household income. For SDG6, all electricity-generating technologies (PV, wind, and nuclear) show higher co-benefit indicator densities because a larger share of their industries are service industries which tend to have higher water use efficiencies (indicator 6.4.1, “Change in water use efficiency over time”) than manufacturing industries^36^^,^^37^ (see section location dependency of SDG linkages for a discussion of country dependencies). In contrast, the deployment process for clean cookstoves is simpler and involves fewer industries. For SDG7, the same mechanism is at play (higher share of service industries that can influence energy indicators even if components are imported). PV scores slightly higher because it has the highest share of service industries among all electricity-generating technologies, combined with a high prevalence of low-energy intensity industries (indicator 7.3.1). For SDG8, PV scores slightly higher than other technologies because a larger share of PV industries shows potential for informal employment (indicator 8.3.1, “Proportion of informal employment in non-agriculture employment, by sex”). For SDG9, a higher potential for growth in small-scale industries is among the reasons for PV’s higher co-benefit density. Additionally, PV has a higher share of high-tech industries among its non-manufacturing industries. (Note that the cells representing linkages between manufacturing industries and SDG indicators are set to zero in this scenario since components are not assumed to be manufactured locally. Thus, any effect would not occur within the boundary of the region studied.) For SDGs 11 and 12, differences between solar PV and wind (higher score) and cookstoves and nuclear (lower score) are driven simply by the fact that current generations of nuclear fission reactors are not suitable for deployment in sustainable buildings (SDG11) due to their scale, and that “environmentally sound” technologies (SDG12) are defined as renewable energy technologies. In that sense, the results are not necessarily indicative of technologies’ suitability to support sustainable cities and consumption in general. Rather, these results derive from specific definitions adopted within the SDG indicator framework that could change over time. In addition to sustainable buildings, for example, SDG11 could focus on sustainable communities more broadly, which could include larger scale zero-carbon technologies such as nuclear.

For trade-off densities, clean cookstoves and nuclear fission show higher scores compared to other technologies. Trade-off densities for clean cookstoves and SDG8 are higher due to a) a higher share of non-manufacturing industries with below-average hourly earnings (indicator 8.5.1, “Average hourly earnings of female and male employees, by occupation, age and persons with disabilities”) compared to other energy technologies considered here; these industries include, for example, soil preparation and gathering of forest products; b) a higher share of deployment industries with child-labor risks (indicator 8.7.1, “8.7.1 Proportion and number of children aged 5–17 years engaged in child labor, by sex and age”), such as mining, agriculture, and freight transportation; and c) a higher share of deployment industries with elevated injury risks (indicator 8.8.1, Frequency rates of fatal and non-fatal occupational injuries, by sex and migrant status), including copper and iron ore mining, metals manufacturing, freight transportation, warehousing, and agriculture. Cookstove trade-off densities for SDG9 are higher only due to the higher share of ${CO}_{2}$-intensive industries (indicator 9.4.1, “${CO}_{2}$ emission per unit of value added”) among cookstove deployment industries.

For SDG6, trade-off densities in the component import scenario are driven mainly by number of deployment and usage-related industries in each technology. For PV, this number is low, and thus the lower water use efficiency of PV electricity generation has a greater effect on PV’s summary density score. This result is not meaningful regarding any of PV’s water usage characteristics, however. PV’s water usage is generally below that of other energy technologies even for future, high-penetration scenarios.^38^ The only reason a “-1” is assigned here is that PV’s water use efficiency, like that of other energy technologies, is still significantly lower than the average water use efficiency of most countries (see supplementary information section S-8) and adding a marginal unit of PV would thus decrease rather than increase water use efficiency.

Wind, PV, and nuclear show similar results across different SDGs and indicators in the component import scenario (Figure 4), with a few exceptions among the indicators associated with SDGs 8 and 9. For example, for indicator 8.3.1. (“Proportion of informal employment in non-agriculture employment”), wind and nuclear show a lower co-benefit density than PV and cookstoves. Informal employment is more common in the large network of component suppliers, retailers, and technicians offering small-scale PV end-use products, and in cookstove logistics and sales, than it is for wind which requires mostly high-skilled labor.^39^^,^^40^^,^^41^

#### Local manufacturing scenario

In the local manufacturing scenario, the differences between technology-specific co-benefit densities are smaller, while trade-off densities differ across the energy technologies considered (Figure 5). These results arise from the similarly important role of both manufacturing and service industries in all energy technologies considered. When they grow, all manufacturing and service industries have potential to contribute to economic growth (indicator 8.1.1, “Annual growth rate of real GDP per capita” and indicator 8.1.2, “Annual growth rate of real GDP per employed person”) and employment (indicator 8.5.2, “Unemployment rate, by sex, age, and persons with disabilities”), for example. It is therefore not surprising that all technologies built and deployed by such industries score similarly for SDG8.

The explanation for the similarity of results for SDG9 across technologies is different. Rather than showing similar results across all indicators, better performance of some technologies for some indicators is offset by worse performance for other indicators. For example, PV’s co-benefit density is lower for indicators related to transportation of goods and manufacturing value generated (indicators 9.1.2, “Passenger and freight volumes, by mode of transport”, 9.2.1, “Manufacturing value added as a proportion of GDP and per capita”, and 9.2.2, “Manufacturing employment as a proportion of total employment”), but higher for indicators related to small-scale industries (indicators 9.3.1 and 9.3.2) and research and development (indicators 9.5.1, “Research and development expenditure as a proportion of GDP”, and 9.B.1., “Proportion of medium and high-tech industry value added in total value added”). In other words, PV has a smaller share of manufacturing industries compared to the other technologies considered, but a larger share of R&D-intensive and high-tech industries e.g., six different electrical equipment and semiconductor-related industries, based on the available literature.^42^^,^^43^^,^^44^

Taking a closer look at metrics related to innovation, all three electricity-generating technologies (PV, wind, and nuclear) score similarly and higher than clean cookstoves. This difference is driven primarily by the higher share of R&D-intensive and high-tech industries in the electricity-generating technologies considered. Minor differences between the electricity-generating technologies arise from the higher share of industries classified as mining or primary metals manufacturing in nuclear compared to PV and wind. Neither mining nor primary metals manufacturing are ranked among the most R&D-intensive industries,^42^^,^^43^^,^^44^ and the numerous nuclear mining and manufacturing industries thus lead to a lower R&D intensity score.

For SDG1, co-benefit densities for cookstoves are higher due to the larger prevalence of simple, non-high-tech industries with potential to increase the income of poor households (as the requirements for expensive qualifications are lower). For SDG11, the reason for the difference between PV and wind and the other technologies is the same as for the import scenario—PV and wind are small and modular and can be deployed at the building level, in contrast to nuclear. Trade-off densities in the local manufacturing scenario are highest for clean cookstoves for several SDGs considered here. For SDG5, the reason is the large share of industries with fewer women in managerial positions. For SDG6, the reason is the comparatively smaller share of service industries with high-water use efficiency in the clean cookstove supply chain. For SDG12, wind and nuclear require a slightly larger number of industries producing hazardous waste in proportion to the overall number of industries required. For SDG14, the reason for the higher cookstove score is the comparatively larger share of manufacturing industries with higher potential for coastal eutrophication and marine acidification, as compared to the more service industry-centric electricity-generating industries.

#### Industry contributions to densities in the local manufacturing scenario

While sections component import scenario and local manufacturing scenario summarize the high-level results for each technology and SDG, here we examine the underlying contributions of industries to these results. To study this, we calculate co-benefit and trade-off densities (see Equation 1) for all industries and SDG indicators, and summarize the results at the 2-digit NAICS level. The number of industries can be viewed as a measure for the breadth of efforts required to enhance co-benefits or mitigate trade-offs.

**Figure 6:**
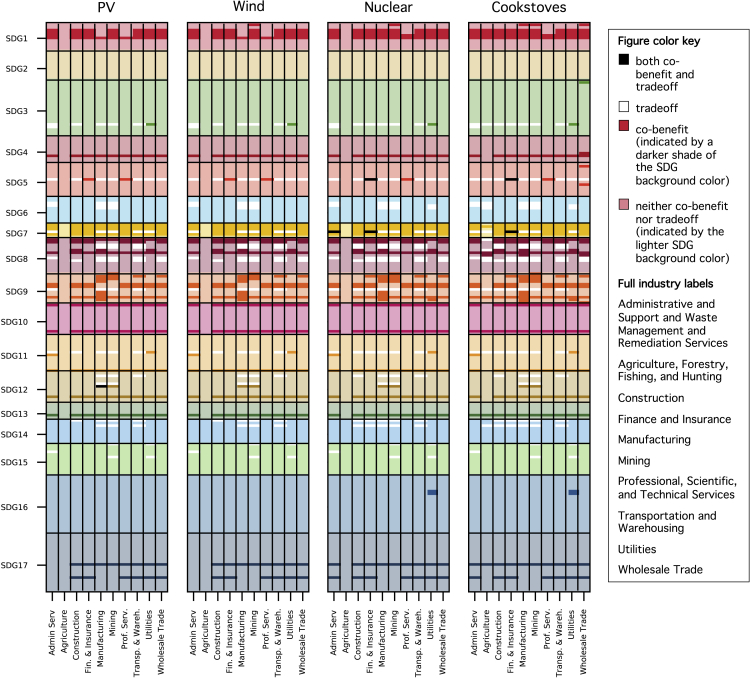
Indicator densities for individual industries in the local manufacturing scenario Densities are calculated for each industry, aggregated for each industry category (matrix columns) and shown separately for all SDG target indicators (matrix rows). The four matrices indicate the results for each of the four technologies. Black cells represent industries that show potential to induce both co-benefits and trade-offs. White cells denote trade-off densities. Co-benefit densities are shown as cells that feature a darker shade of the base color used to illustrate the rows of the indicators belonging to a certain SDG. Lighter base colors without additional coloring are used for industries with neither co-benefits nor trade-offs. (Note that horizontal gridlines are drawn to indicate the rows that belong to a certain SDG but not shown between each row for ease of interpretation. Indicator labels are not included for each row on the y axis due to space constraints.)

As shown in Figure 6, the diversity of industries contributing to the densities varies between the SDGs, but the results are similar across technologies. In the case of SDGs 4, 10, 12, and 13, most of the ten industries show potential to induce co-benefits with these SDGs, meaning that the co-benefit densities in Figure 4 are driven by many different industries rather than just a few. In contrast, in the case of SDG 1, 8, 9, and 17, only some industries (e.g., a handful of high-tech industries for SDG9) contribute to potential co-benefits.

Co-benefits are driven mainly by service industries and their potential effects on SDG8 indicators, as well as by manufacturing industries and their potential effects on SDG9 indicators. Service industries affecting SDG8 include, for example, industries related to site preparation, installation, component supply, and end-of-life management. The manufacturing industries in our set also show potential to induce trade-offs with SDG6 (“Clean Water and Sanitation”). Overall, the co-benefit and trade-off densities of industries show fairly similar patterns across the four technologies. Whereas most of the trade-offs derive from the manufacturing industries (metals and electrical components), those industries also offer co-benefits especially for SDG9. Service industries tend to mainly contribute to co-benefit densities though they also show potential to induce trade-offs, especially for SDGs 3 (due to potential contributions to air pollution from freight transport and waste incineration), 11 (due to potential contributions of freight transport to fine particulate matter pollution), 14 (due to potential contributions of ${NO}_{x}$ and ${CO}_{2}$ emissions from freight transport to coastal eutrophication and ocean acidity), and 15 (due to the spatial footprint of energy technologies and its potential impact on forest areas). In the interest of brevity, we have included the industry contributions here only for the local manufacturing scenario, but the same analysis could be carried out for the component import scenario.

### Location dependency of SDG linkages

The majority of our results hold independent of location due to the global scope of our literature review. However, some results show a higher dependency on the location in which linkages are evaluated than others. Location dependencies stem from a) significant differences in current indicator values across countries, such that growth in an industry with a certain indicator value has the potential to induce beneficial impacts in some countries and detrimental impacts in others; b) definitional differences across countries (e.g., what counts as hazardous waste in the context of SDG12 or as high-tech industry in the context of SDG9); and c) geographical differences that affect the degree to which a country is sensitive to coastal eutrophication (SDG14) or changes in land use and mountain biodiversity (SDG15). We discuss specific location dependencies for each SDG and indicator below.

For air pollution indicators (3.9.1 and 11.6.1), deploying zero-pollution technologies such as wind and solar PV will have a greater effect in areas where current electricity generation has high particulate matter-intensity. For indicators related to water use efficiency, countries with very low water use efficiency have greater potential for improvement induced by investments in energy technology industries. The reason is that the water use efficiency of most manufacturing industries is higher than the relatively low national average in these countries, contrary to countries with higher national averages (e.g., Luxembourg, UK, Switzerland, and Denmark), where only service industries have greater water use efficiency but not manufacturing industries. For example, the water use efficiency of several developing nations in South America (Chile and Nicaragua), Asia (Indonesia and Vietnam), and Africa (Niger, Sudan, and Mali) is below 10 USD/ $m^{3}$ (see supplementary information Figure S1), which is below standard manufacturing industry water use efficiencies. In these countries, technologies where a high fraction of indicator densities comes from manufacturing industries will show greater potential for positive change in the form of higher co-benefit densities for SDG7 and 6. Similar to the country dependency of linkages between SDG7 and 6, less economically developed countries with lower R&D intensities show greater potential for co-benefits from industry investments because relative to these countries’ national average R&D intensity, a larger number of industries have a greater R&D intensity. Such countries include, for example, Brazil, India, and Pakistan.

For SDG12, country dependencies may arise from differences in the definitions of hazardous waste (indicator 12.4.1). Our results are based on the EU’s list of wastes and the specific waste categories labeled as hazardous therein (see supplementary information section S-10.2); other regions or countries may use broader or narrower definitions that could include or exclude certain industries in our set. For indicator 12.5.1. (recycling rate), countries with low national average recycling rates will show greater potential to experience positive change due to investments in energy technologies, because a greater number of industries exhibit high recycling rates relative to these countries’ national average. Finally, countries with larger fractions of mountainous areas (SDG15) will be affected more strongly by deployment of energy technologies in mountainous regions than other countries. Such differences would not affect the results presented here because potential linkages either exist or not, but they could affect results based on the strength of individual industry-indicator linkages.

### Measures to enhance co-benefit and weaken trade-off linkages

In addition to identifying potential linkages, conceptualizing linkages as industry impacts is also useful to prioritize measures that may create or enhance co-benefit linkages and weaken trade-off linkages. Here, we briefly discuss such measures, and the extent to which they apply to the energy technologies in our set.

**Figure 7:**
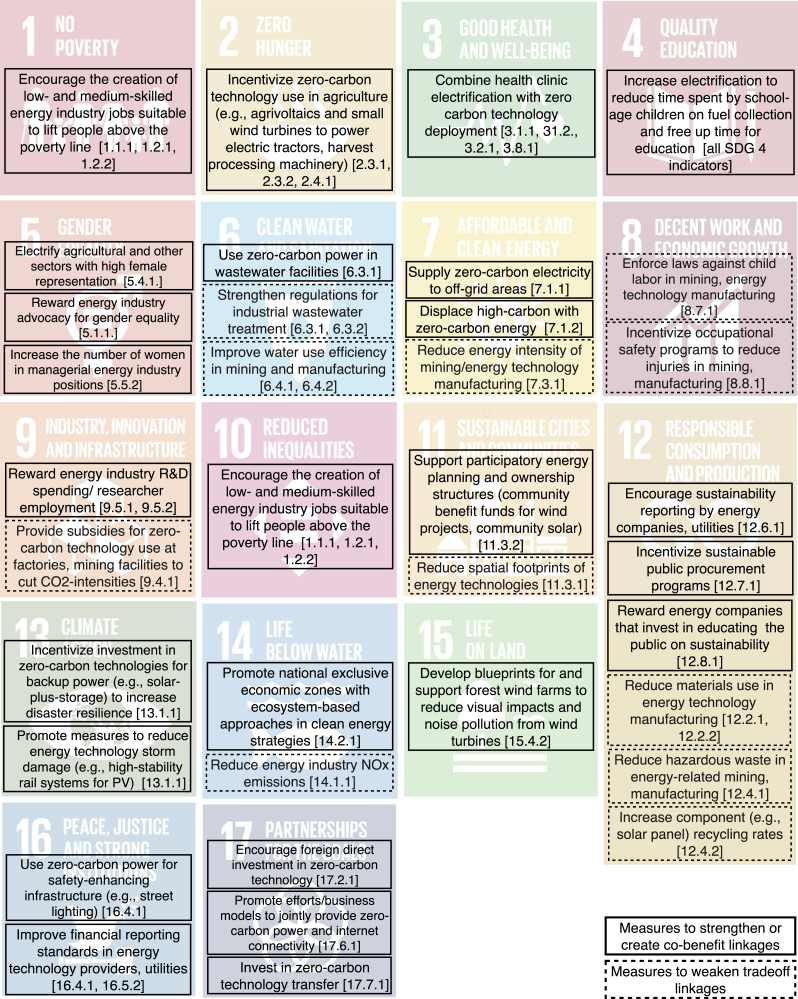
Example measures to enhance co-benefit and weaken trade-off linkages between SDG7 and other SDGs Examples are derived from comparatively low co-benefit densities (indicating that more can be done to induce technology impacts on SDG indicators) and comparatively high trade-off densities (indicating a need to reduce technology impacts on SDG indicators). “Energy companies” refers to all types of private businesses involved in energy technology component manufacturing, deployment, usage for electricity and heat generation, and end-of-life management.

Looking at Figures 4 and 5, it is apparent that measures to strengthen existing co-benefit linkages could focus on linkages between SDG7 and SDGs 1, 4, 6, 7–13, and 17. This is based on the non-energy SDGs in Figures 4 and 5 where densities are already positive but below one, which means that not all possible connections between industries and indicators contribute to the density value. In addition, measures to create co-benefit linkages could focus on areas where our review did not currently identify direct linkages, including SDGs 2, 3, 15, and 16. To weaken trade-off linkages, linkages between SDG7 and SDGs 3, 5–9, 11, 12, 14, and 15 appear important target areas. A list of measures derived from this assessment is shown in Figure 7.

The majority of these measures can be applied to all four energy technologies in our set, but there are some noteworthy exemptions. PV and wind are more suitable for sustainable farming systems (linkage between SDG7 and 2) than nuclear fission, for example (unless small-scale nuclear reactors are commercialized and small enough for agricultural use). A similar conclusion can be drawn for other linkages, including those between SDG7 and SDGs 3, 6, and 16, as small-scale wind turbines or PV are better suited for supplying zero-carbon power to health clinics, wastewater treatment facilities, and street lighting than nuclear.

### Potential SDG linkages not captured by SDG indicators

In addition to identifying potential linkages represented within the UN indicator framework, analyzing technologies in terms of their industries and services is also useful to identify potential linkages not directly reflected in any of the official indicators. While a formal analysis of these linkages is beyond the scope of this study, here we discuss these linkages qualitatively, focusing on six topics (growth rates, supply chain vulnerabilities, energy equity, innovation ecosystems, institutional support, and resilience and climate change adaptation).

#### Growth rates and scalability

Industries have been shown to scale at different rates.^45^ Historically achieved growth rates may thus be relevant to assessing a technology’s suitability to meet climate change mitigation targets, considering the limited time remaining to decarbonize the global energy system. Global solar PV capacity, for example, has grown at twice the rate of wind power over the past decade.^46^ Acknowledging this difference in indicators (e.g., under SDG13 or SDG9) could lead to different linkages depending on the choice of clean energy technology. From a country perspective, countries that achieve high growth rates in clean energy markets but start from a small base may want to see this achievement reflected in sustainability indicators sooner rather than later. However, these countries would continue to score low in terms of the renewable energy share in the total final energy consumption (indicator 7.2.1), not differentiating themselves from other countries with slower growth in clean energy industries. The inclusion of clean energy technology growth rates (e.g., multi-year averages), for example, could address this, although indicator designs should also avoid incentivizing aggressive short-term policies and boom-and-bust cycles.

Relatedly, project success rates also differ across technologies.^66^ Nuclear fission reactors, thermal power plants, hydropower projects, and mining facilities, for example, have exhibited larger schedule and cost overruns than other projects.^47^^,^^67^ SDG indicators could be developed to capture the ability of industries to deliver on schedule and on budget in a specific geographical context, thereby contributing to multiple SDGs (e.g., 1, 8, 9, and 13 in addition to 7).

#### Supply chain vulnerability

Materials availability is key to maintaining clean energy industry growth rates, with certain wind turbine generator designs particularly affected due to their use of rare earth metals. Although materials usage is tracked by several indicators across multiple SDGs (SDGs 8 and 12), there is not a single metric in the SDG framework to capture metals criticality or other types of supply chain vulnerabilities in the form of frequent and/or extreme price spikes, lack of materials substitutability, and high import dependencies. Many of these dimensions of supply chain vulnerability are both technology- and country dependent, and inclusion in sustainability indicator frameworks could therefore motivate countries to invest in the robustness of their supply chains.

#### Energy equity

Energy technologies with similarly low environmental impacts may differ in other ways that affect their perception and support by local communities. For example, local stakeholders may place more emphasis on the availability of community benefit funds (or related policy frameworks to help ensure local socio-economic benefits from energy projects) than on macro-economic impacts when evaluating new energy projects. Standardized procedures for community involvement are somewhat more prevalent for wind power than for other energy technologies.^48^ Safety considerations in using electricity systems (e.g., avoiding injuries from illegal power line extensions in developing countries), the ability to minimize electricity theft, and expectations regarding the funds needed for potential repairs after extreme weather events may be similarly important as environmental goals.^49^^,^^50^ Developing indicators that measure technology aspects that are important to individuals and communities, especially marginalized groups, is thus critical. Since giving separate consideration to all community-level priorities may be beyond the scope of standardized SDG indicators, developing composite indices representing various “community-level” benefits of technologies could be one promising direction for expanding the UN indicator framework.

#### Innovation ecosystems and employment

The ability to create new, useful knowledge and to promote entrepreneurial activity has been shown to differ across technology ecosystems. For example, some technology ecosystems generate more patents than other industries.^51^ However, since neither SDG8 nor 9 includes indicators related to invention and innovation rates, these differences would not be captured by the SDG framework. While changes in R&D intensity and in the prevalence of high-tech enterprises are covered by SDG indicators under SDGs 8 and 9, both metrics are focused on inputs to the innovation process rather than outputs in terms of new ideas and technologies. Including metrics that capture patenting rates and start-up funding, for instance, could spark country-level interest in strengthening local innovation ecosystems using SDG-related funding sources.

#### Institutional support

Institutional support for technology market growth and performance monitoring may have been an important factor in the rapid scaling of technologies in certain countries, including photovoltaics in Germany and the US as well as nuclear fission in France. In all three countries, institutions such as Fraunhofer, the National Renewable Energy Laboratory (NREL), as well as Électricité de France and the French Alternative Energies and Atomic Energy Commission, for example, were actively involved in research and development, data collection and sharing, and continuous monitoring of solar PV and nuclear technology performance (e.g., in the form of NREL’s cost benchmarks or Fraunhofer reports on solar PV markets). Direct connections are difficult to draw, but these institutions likely played a role in the successful scaling of energy industries. It is thus reasonable to assume that the degree of institutional support for new and emerging technologies may influence linkages with SDGs 16 and 17. However, institutional support for technology development and market scaling is not captured by the SDG indicator framework.

#### Resilience and climate change adaptation

The deployment of electricity-generating technologies with higher resilience to natural disasters can reduce economic damages due to extreme weather events. However, SDG13 only covers direct effects on human life (Indicator 13.1.1, Number of deaths, missing persons, and directly affected persons attributed to disasters), rather than indirect economic effects due to power outages, which may be reduced in distributed energy systems as compared to centralized systems vulnerable to few, large-scale power plants and transmission lines. Relatedly, power grid performance and resiliency measures are not covered by SDG7. Indicators could cover, e.g., the share of electricity generation capacity with black start capability (ability of generation to restart parts of the power system to recover from a blackout), as well as the duration, frequency, and predictability of power grid outages. Blackstart capabilities differ across energy technologies, and including metrics like this one could thus lead to different results on SDG linkages across technologies.

## Discussion

### Conclusions

This research examines the potential of energy technology investments to induce linkages between the primary investment goal, SDG7 (“Affordable and Clean Energy”) and the other 16 SDGs. We introduce a method for screening technologies regarding their potential to induce SDG linkages based on the manufacturing and service industries required over the life cycle of these technologies, and the risks and opportunities these industries pose when evaluated against SDG indicators using currently available literature. We apply the method to an example set of technologies (PV, wind, nuclear, and clean cookstoves) to investigate how SDG linkages are shaped by technology characteristics, and we use this bottom-up perspective on SDG linkages to discuss the suitability of the SDG indicator framework for sustainability-focused technology evaluation more generally.

We find that technologies’ potential to create linkages between SDG7 and non-energy SDGs is affected by a diverse set of factors including a technology's unit and industry scale, requirement for high-tech and R&D-intensive industries, industry wage and skill levels, particulate and carbon emissions, as well as water and waste intensities of manufacturing industries. For some non-energy SDGs, linkages to SDG7 are driven by only a few industries, potentially simplifying activities to monitor SDG linkages, while for other SDGs, all industries in technologies’ supply networks contribute. Overall, we observe the largest number of potentially co-beneficial linkages between SDG7 and SDGs 1, 8, 9, and 10, and the largest number of potential trade-off linkages between SDG7 and SDGs 6, 8, 12, and 14.

SDG linkages not only differ by the number of industries that show potential to influence an SDG but also by the number of SDG indicators in which this influence is likely to manifest. While most linkages between SDG7 and other SDGs are rooted in multiple SDG indicators (i.e., multiple indicators show potential to be affected by technology industries), linkages for some SDGs are driven only by one or two indicators and are thus highly sensitive to the choice of indicator. These SDGs include health, education, gender equality, sustainable cities, climate change, and institution-related goals (SDGs 3, 4, 5, 11, 13, and 17). Whether the few indicators potentially affected by a technology are ultimately considered important can also depend on the location. For example, installing clean energy technologies in underdeveloped areas to improve access to energy and transportation services in slums will induce stronger linkages between SDG7 and SDG11 than in locations where such infrastructure already exists (i.e., fewer SDG11 indicators would be affected). Such observations suggest that linkages between two SDGs are not universal but should be considered in the local context and given specific priorities defined by planners and policy makers.

The prevalence of manufacturing compared to service industries in a technology’s supply network is an important factor in determining linkages. Service industries show potential primarily for inducing co-benefits between SDG7 and 11 other SDGs, while manufacturing industries induce both co-benefits and trade-offs. We observe the largest share of potentially beneficial industry-indicator linkages between SDG7 and SDGs 1, 8, 9, and 10. If components are manufactured locally, the share of trade-off linkages in all possible linkages increases, with the highest share of trade-off linkages observed between SDG7 and SDGs 6, 8, 12, and 14.

Overall, this paper is intended as a starting point to demonstrate challenges and opportunities in screening technologies for SDG linkages using SDG indicators. By linking technology components to SDG indicators, we analyze SDG linkages “from the bottom-up”, breaking down the influence of technologies on SDGs into industries and deployment services that can be targeted by researchers (for more detailed study) and policymakers (for regulatory intervention). For example, supporting clean energy and clean water goals at the same time may be achieved by enhancing water use efficiency in specific manufacturing industries, or by shifting economic activity from less water-efficient manufacturing industries to more water-efficient deployment services altogether.

Conceptualizing SDG linkages through technologies can also support advanced thinking about tensions between climate, economic, and broader development goals. For example, while the cost of factory-manufactured hardware components has fallen steadily (e.g., in PV, wind), the growing share of deployment costs in energy technologies has been identified as a potential barrier for decarbonization.^52^^,^^53^ At the same time, and as demonstrated by our analysis, deployment services can enhance SDG linkages. Strategies should therefore be developed that maximize cost reduction while also enabling progress toward non-cost targets. For example, automating construction processes or combining information (“smart”) and energy technologies could reduce costs over the life cycle while also supporting SDG9.

### Comparison to previous analyses of SDG linkages

Our results differ from those of previous analyses at both the SDG level (i.e., the number and specific SDGs for which we identify linkages) and at the indicator level. Overall, our approach points to a larger number of linkages between SDG7 and other SDGs compared to other studies. While a recent report identifies no connections induced by SDG7 energy technologies to SDGs 4, 5, and 10,^15^ for example, our analysis points to possible linkages stemming from renewables used for school electrification. Our analysis also finds a similar amount of linkages across renewables and nuclear, while nuclear shows fewer linkages in prior work.^54^ Similarly, while in a previous paper, SDG7 appears equally connected to SDGs 1, 14, and 15 in terms of the number of sub-targets affected,^15^ for example, considering industry-specific indicators suggests a much larger share of potentially co-beneficial linkages between SDG7 and 1 as compared to 14 and 15. This result is rooted in the larger share of industries directly affecting poverty-related metrics used for SDG1 as compared to metrics related to marine life (SDG14) and life on land (SDG15). Similarly, while trade-offs between SDG7 and SDGs 4 and 15 appear most likely based on the number of targets negatively affected, our analysis identifies the largest share of trade-off linkages for an entirely different set of SDGs (5, 6, 7, 8, 12, and 14). These differences indicate that analyzing SDG linkages in terms of technology choices and industry impacts can lead to different results as compared to expert elicitation and other methods. This may be so because experts discard the impact of certain industry-indicator connections as unimportant based on their knowledge (which could be seen as desirable), or because some connections are omitted unintentionally due to cognitive limitations. Future work could explore these differences in more detail.

There are also similarities to previous results that help validate our analytical framework. For instance, we identify twice as many potential co-benefit linkages as trade-off linkages at the indicator level, similar to a prior analysis at the SDG target level.^15^ Another similarity is that the SDG with the most targets positively affected by energy technology deployment in pursuit of SDG7, SDG9, is also among the set of SDGs with the highest co-benefit scores in our work. These comparisons show that some conclusions on SDG linkages may be robust to the type of analytical framework used, while others are more sensitive.

## Limitations of the study

### Linkage strength

This blueprint analysis assigns uniform weights to all linkages between industries and indicators in order to enable the coverage of all SDG indicators in one single study. It is clear, however, that technology life cycles involve different levels of activity across industries (e.g., more or less materials usage and manufacturing labor) per unit of service provided, and that industries differ in how strongly they affect various SDG indicators. Identifying the most relevant linkages in a practical context would thus require an evaluation of how technology investments are distributed across industries, and a method to quantify the effects of industry growth on SDG indicators. Direct modeling of indicator changes is one approach for linkages where historical data are available for model calibration and/or change mechanisms are fully understood and can be modeled directly (e.g., emissions reductions from the displacement of high- with low-carbon electricity); inviting experts to rate the linkages derived from the literature or consulting stakeholders in focus groups are other approaches.

Regardless of the method for quantifying the strength of a linkage, normalizing indicator changes (e.g., to historical trends) and considering context-specific priorities will be important to meaningfully compare linkages. For instance, a small percent change in some indicators (e.g., GDP growth and other growth rate indicators; R&D expenditure as a proportion of GDP; indicators expressed in number of countries) may be broadly considered as more influential than the same change in other indicators; stakeholder values assigned to indicators may also differ.^55^

### Future work

#### Application to other technologies

Our framework is sector agnostic in the sense that it can be applied to any sector where technology investments are used to support progress toward an SDG, not just in clean energy. Artificial intelligence technologies may be an interesting future focus, for instance, to help explore linkages between gender-, equity-, and institutions-related goals (SDGs 5, 10, and 16) and SDGs 8 or 9. Similarly, analyzing agricultural- and health-related technologies using the approach presented in this paper could be useful to explore linkages between poverty reduction, health improvements, and other SDGs.

#### Analysis of geographical and timescale

Considering impacts of technology choices on SDG linkages at different geographical scales and over a variety of timescales is another challenge. Many technology-induced risks and opportunities will be present regardless of where a technology is deployed, but how these risks and opportunities will be managed depends on local environmental regulations, economic policies, and other regional factors. The impact of technology choices on SDG linkages will likely also change with time, e.g., depending on changes in industries’ capital and labor intensity. While our blueprint analysis only considers the effect of marginal changes (all else equal), future work could derive edge weights for specific time periods and phases of economic development.

#### Development of alternative indicator frameworks

Analyzing SDG linkages through the lens of technologies can be useful to evaluate the suitability of current SDG indicators for capturing technologies’ diverse socio-economic and environmental impacts. As we discuss, the UN framework is not ideally suited to capture technologies’ scalability, growth rates, disaster resilience, equity implications, and project delivery on schedule on budget, all of which can induce positive and negative linkages to other SDGs. No indicator framework can capture all aspects of technology investments that different domain experts or stakeholders may consider important; yet the urgency of the climate crisis and the critical role of zero-carbon technology point to a need to re-evaluate sustainability indicator frameworks from a technology-centric perspective. Future work could analyze linkages using an indicator framework expanded relative to the UN’s. Such analyses may reveal larger differences between technologies’ potential to induce linkages than observed here, as well as new drivers of linkages. Using stakeholder workshops to better understand the priorities of local communities and companies investing in energy technologies or other SDG-related interventions could point to indicators more indicative of impacts that matter on-the-ground and may affect adoption dynamics.

## STAR★Methods

### Key resources table

**Table:** 

REAGENT or RESOURCE	SOURCE	IDENTIFIER
**Other**		

NAICS Code List	NAICS Association	https://www.naics.com/search/
Criteria Air Pollutants by sector	United States Environmental Protection Agency	https://www.epa.gov/air-emissions-inventories/2017-national-emissions-inventory-nei-data
National average water use intensity by country	United Nations	https://sdg6data.org/en/indicator/6.4.1
Research and development expenditure	World Bank	https://data.worldbank.org/indicator/GB.XPD.RSDV.GD.ZS

### Resource availability

#### Lead contact

Further information and requests for resources should be directed to and will be fulfilled by the lead contact, Magdalena M. Klemun (magdalena@ust.hk).

#### Materials availability

This study did not generate new materials.

#### Data and code availability

• The attached supplemental information includes all datasets analyzed to perform this study
• This study does not report original code
• Any additional information is available from the lead contact upon request

### Methods details

#### Technology representation

We differentiate industries and services based on the North American Industry Classification System (NAICS). We draw on bill-of-materials (BOM) used in life cycle analyses and bottom-up-cost models to identify each technology’s components, industries, and services. For photovoltaic (PV) systems, we use the BOM presented in the National Renewable Energy Laboratory’s annual PV cost benchmark reports (e.g.,^56^). For wind turbines, we draw on the component and materials list used in the life cycle analysis of a standard wind turbine sold by Vestas.^57^ For nuclear power plants, we use the list of NAICS code industries specific to employment generated by nuclear industry and construction,^58^ as well as the cost and component breakdown provided for light-water reactors in the Department of Energy’s Energy Economic Database which was maintained from 1976-1987.^59^ For cookstoves, we use the components listed in the ACE clean cookstove’s user manual and fill in the gaps with information from a life cycle assessment of ${LiFePO}_{4}$ batteries.^60^^,^^61^ Note that we draw on these diverse sources because there is no single source enlisting energy technology components and industries in a standardized manner. However, while the number of technology components is sensitive to the level of technology decomposition, the number of industries (which is the important parameter for the analysis) is not. For example, it does not matter whether a solar panel is broken down into cells, frame, and back glass, or listed as one single component, as engineering knowledge is required regardless to identify the industries supplying these components. Finally, while the sample of technologies analyzed here is not exhaustive, it covers several of the largest sources of estimated zero-carbon technology capacity growth under nationally determined contributions and net-zero emissions targets,^54^^,^^62^ while omitting coal and natural gas-based technologies due to their high ${CO}_{2}$ and methane emissions impacts.^32^^,^^63^ Future work could apply our approach to other supply- and demand-side investments made in pursuit of SDG7, including hydropower, geothermal, biomass, energy efficiency, and demand response.

#### Evaluation of SDG linkages

We consider both beneficial linkages (‘co-benefit linkages’) and detrimental linkages (‘tradeoff linkages’). Co-benefit linkages are assumed to be possible if we find evidence in the peer-reviewed academic and gray literature for a technology industry influencing an SDG indicator in a beneficial direction. Our literature search is focused on causal and functional rather than correlational linkages between industries and indicators. In other words, we search for mechanisms or industry characteristics that are likely to connect industries to SDG indicators, or have done so in the past. If we find such evidence, we set the corresponding matrix element ‘+1’. These uniform weights are a simplification of real-world impacts of industries on SDG indicators, which likely differ in strength. We adopt this simplification here to facilitate coverage of several technologies and all SDGs in a single study. We discuss possible extensions of our work to compute non-uniform weights in section limitations of the study.

Our search for published evidence does not constitute a formal review of current knowledge on potential industry-indicator linkages since our work is not focused on the strength of these linkages. One single published journal paper or report was considered sufficient to indicate the potential of a manufacturing activity or service to affect an SDG indicator. References used to support individual linkages are given in supplementary information sections S-3-S17 and supplementary information Tables S16–S47.

Tradeoff linkages are assumed to be possible if we find evidence in the peer-reviewed literature for a technology industry influencing an SDG indicator in direction that is likely undesirable. We then set the corresponding matrix elements equal to ‘-1’. For example, growth in industry 484,122 (‘General Freight Trucking, Long Distance’) to deliver PV system or other energy technology components has the potential to induce undesirable change in indicator 8.8.1 (‘Frequency rates of fatal and non-fatal occupational injuries’) because transportation is the third most dangerous industry by death rates and the most dangerous industry in terms of non-fatal injury rates.^64^ We note that industries will have different degrees of influence on an indicator, which is not captured by the three-point scale we use. Future work could model specific technology manufacturing and deployment pathways and quantify related effects on SDG indicators, assuming data is available for such an analysis. The degree of influence could then be derived from the percent change in each indicator due to increased activity in each industry.

Since the impacts of technology deployment on development indicators will depend on whether technology components are manufactured locally or imported, we consider two scenarios. In the local manufacturing scenario, all technology components are assumed to be manufactured within the boundary of the region, country, or city in which SDG linkages are analyzed. In contrast, the import scenario assumes that only final construction and installation activities occur at the location of interest; technology hardware components are assumed to be imported from locations outside the system boundary. We consider these two scenarios as boundary cases as they span a range of possible technology compositions, locally sourced and imported, that may be chosen in real-world energy projects. We do not aim to evaluate the likelihood of each scenario since it depends on the technology and the regional context and manufacturing track record. For nuclear power plants in developing countries, for example, proliferation risks and the lack of industrial capabilities make local manufacturing scenarios appear unlikely in the near-term (while clean cookstoves are already manufactured locally).

For each combination of technology and SDG indicator, we define a technology-indicator density $d_{i}$ to measure the number of connections with documented potential for influence over the total number of possible connections:

(Equation 1) $$d_{i}=\frac{1}{n}\sum_{k=1}^{n}l_{k}\text{,}$$

where n is the total number of industries in a technology’s supply network and $l_{k}$ is an industry-specific indicator variable that is either ‘+1’ or ‘-1’. We compute $d_{i}$ separately for indicators that are influenced by technology industries in a beneficial way (‘co-benefit density’) and for those that are influenced in a detrimental way (‘trade-off density’).

To summarize the potential of each energy technology to create a linkage between SDG7 and other SDGs across all indicators of a particular SDG, we define an aggregated density metric, the linkage density d:

(Equation 2) $$d=\frac{1}{s}\sum_{i=1}^{s}d_{i}\text{.}$$

Here, s is the total number of indicators associated with a particular SDG. Equation 2 sums up the densities across all indicators of a particular SDG, resulting in 17 values for each technology in our technology sample. While these values are a measure for prevalence of industry-indicator linkages in each technology-SDG network (i.e., how many connections out of all possible links between industries and indicators are likely to be meaningful in the sense that they may lead to indicator changes), they do not constitute a measure for linkage strength. For example, while there may be several industries influencing multiple indicators for a particular combination of technology and SDG, a single connection between one industry and one indicator may ultimately represent a stronger link. We discuss the issue of linkage strength, and how future work could build on this paper to address it, in Conclusions.

In the supplementary material, we discuss specific industry-indicator linkages separately for each SDG. Tables S16–S47 give the data sources and references used to assign a value (‘1’ for desirable change, ‘-1’ for undesirable change) to each industry-indicator matrix element. Each table covers one technology and one SDG, with the assigned value for each potential link listed by SDG indicator.

## References

[1] Collste D., Pedercini M., Cornell S.E. (2017). Policy coherence to achieve the SDGs: using integrated simulation models to assess effective policies. Sustain. Sci..

[2] El-Maghrabi M.H., Gable S.E., Osorio-Rodarte I., Verbeek J. (2018).

[3] Nilsson M., Griggs D., Visbeck M. (2016). Policy: map the interactions between sustainable development goals. Nature.

[4] UN Economic and Social (2016). Council. “Report of the interagency and expert group on sustainable development goal indicators”. Stat. Comm.

[5] Deng H.M., Liang Q.M., Liu L.J., Anadon L.D. (2017). Co-benefits of greenhouse gas mitigation: a review and classification by type, mitigation sector, and geography. Environ. Res. Lett..

[6] Jochem E., Madlener R. (2003). Workshop on the Benefits of Climate Policy: Improving Information for Policy Makers.

[7] Nemet G.F., Holloway T., Meier P. (2010). Implications of incorporating air-quality co-benefits into climate change policymaking. Environ. Res. Lett..

[8] Phelps J., Webb E.L., Adams W.M. (2012). Biodiversity co-benefits of policies to reduce forest-carbon emissions. Nat. Clim. Change.

[9] Bollen J., van der Zwaan B., Brink C., Eerens H. (2009). Local air pollution and global climate change: a combined cost-benefit analysis. Resour. Energy Econ..

[10] Gu A., Teng F., Feng X. (2018). Effects of pollution control measures on carbon emission reduction in China: evidence from the 11th and 12th five-year plans. Clim. Pol..

[11] Qian H., Xu S., Cao J., Ren F., Wei W., Meng J., Wu L. (2021). Air pollution reduction and climate co-benefits in China’s industries. Nat. Sustain..

[12] von Stechow C., Minx J.C., Riahi K., Jewell J., McCollum D.L., Callaghan M.W., Bertram C., Luderer G., Baiocchi G. (2016). 2 C and SDGs: united they stand, divided they fall?. Environ. Res. Lett..

[13] Klemun M.M., Edwards M.R., Trancik J.E. (2020). Research priorities for supporting subnational climate policies. Wiley Interdiscip. Rev. Clim. Change.

[14] Nilsson M. (2017).

[15] Nerini F., Tomei J., To L.S., Bisaga I., Parikh P., Black M., Borrion A., Spataru C., Castán Broto V., Anandarajah G., Milligan B. (2018). Mapping synergies and trade-offs between energy and the sustainable development goals. Nat. Energy.

[16] Le Blanc D. (2015). Towards integration at last? The sustainable development goals as a network of targets. Sustain. Dev..

[17] Lusseau D., Mancini F. (2019). Income-based variation in sustainable development goal interaction networks. Nat. Sustain..

[18] Pradhan P., Costa L., Rybski D., Lucht W., Kropp J.P. (2017). A systematic study of Sustainable Development Goal (SDG) interactions. Earths Future.

[19] Griggs D.J., Nilsson M., Stevance A., McCollum D. (2017).

[20] Dawes J.H. (2020). Are the sustainable development goals self-consistent and mutually achievable?. Sustain. Dev..

[21] Ospina-Forero L., Castañeda G., Guerrero O.A. (2020). Estimating networks of sustainable development goals. Inf. Manag..

[22] Moyer J.D., Bohl D.K. (2019). Alternative pathways to human development: assessing trade-offs and synergies in achieving the Sustainable Development Goals. Futures.

[23] Bali Swain R., Ranganathan S. (2021). Modeling interlinkages between sustainable development goals using network analysis. World Dev..

[24] Bennich T., Weitz N., Carlsen H. (2020). Deciphering the scientific literature on SDG interactions: a review and reading guide. Sci. Total Environ..

[25] Madurai Elavarasan R., Pugazhendhi R., Jamal T., Dyduch J., Arif M., Manoj Kumar N., Shafiullah G., Chopra S.S., Nadarajah M. (2021). Envisioning the UN Sustainable Development Goals (SDGs) through the lens of energy sustainability (SDG 7) in the post-COVID-19 world. Appl. Energy.

[26] McCollum D.L., Echeverri L.G., Busch S., Pachauri S., Parkinson S., Rogelj J., Krey V., Minx J.C., Nilsson M., Stevance A.S., Riahi K. (2018). Connecting the sustainable development goals by their energy inter-linkages. Environ. Res. Lett..

[27] Messerli P., Murniningtyas E., Eloundou-Enyegue P., Foli E.G., Furman E., Glassman A., Hernández Licona G., Kim E.M., Lutz W., Moatti J.P. (2019). Global Sustainable Development Report.

[28] Adenle A.A. (2020). Assessment of solar energy technologies in Africa-opportunities and challenges in meeting the 2030 agenda and sustainable development goals. Energy Pol..

[29] Cohen B., Cowie A., Babiker M., Leip A., Smith P. (2021). Co-benefits and trade-offs of climate change mitigation actions and the Sustainable Development Goals. Sustain. Prod. Consum..

[30] Bisaga I., Parikh P., Tomei J., To L.S. (2021). Mapping synergies and trade-offs between energy and the sustainable development goals: a case study of off-grid solar energy in Rwanda. Energy Pol..

[31] Hertwich E.G., Gibon T., Bouman E.A., Arvesen A., Suh S., Heath G.A., Bergesen J.D., Ramirez A., Vega M.I., Shi L. (2015). Integrated life-cycle assessment of electricity-supply scenarios confirms global environmental benefit of low-carbon technologies. Proc. Natl. Acad. Sci. USA.

[32] Laurent A., Espinosa N., Hauschild M.Z. (2018). Life Cycle Assessment.

[33] SDG Dashboard. https://dashboards.sdgindex.org.

[34] Country Diagnostics Working Group https://www.countrydiagnostics.%20com.

[35] United Nations General Assembly (2017).

[36] Progress on water-use efficiency: global baseline for SDG indicator 6.4.1. Tech. rep. Food, Agricultural Organization Of The United Nations, And United Nations Water

[37] GEMI (2019). Tech. rep. Integrated Monitoring Initiative for SDG 6.

[38] Macknick J., Cohen S. (2015).

[39] Kuldeep N., Chawla K., Ghosh A. (2017). https://www.powerforall.org/application/files/8915/6310/7906/Powering-Jobs-Census-2019.pdf.

[40] (2020). Renewable Energy and Jobs - Annual Review 2020.

[41] Shirley R., Otieno M., Nyambura H. (2020).

[42] Galindo-Rueda F., Verger F. (2016).

[43] Hecker D. (2005). High-technology employment: a NAICSbased update. Mon. Labor Rev..

[44] Wolf M. (2016). Tech. rep. Bureau of Labor Statistics.

[45] Wilson C., Grubler A., Bauer N., Krey V., Riahi K. (2013). Future capacity growth of energy technologies: are scenarios consistent with historical evidence?. Climatic Change.

[46] IRENA (2022). https://www.irena.org/-/media/Files/IRENA/Agency/Publication/2022/Apr/IRENA_RE_Capacity_Statistics_2022.pdf.

[47] Braeckman J., Disselhoff T., Kirchherr J. (2019). Cost and schedule overruns in large hydropower dams: an assessment of projects completed since 2000. Int. J. Water Resour. Dev..

[48] Glasson J. (2017). Large energy projects and community benefits agreements-some experience from the UK. Environ. Impact Assess. Rev..

[49] Baker E., Nock D., Levin T., Atarah S.A., Afful-Dadzie A., Dodoo-Arhin D., Ndikumana L., Shittu E., Muchapondwa E., Sackey C.V.H. (2021). Who is marginalized in energy justice? Amplifying community leader perspectives of energy transitions in Ghana. Energy Res. Soc. Sci..

[50] Lo K., Wang G., Leung M.K., Lo A.Y., Hills P., Mah D.N.Y. (2018). Barriers to adopting solar photovoltaic systems in Hong Kong. Energy Environ..

[51] Johnstone N., Haščič I., Popp D. (2010). Renewable energy policies and technological innovation: evidence based on patent counts. Environ. Resour. Econ..

[52] Eash-Gates P., Klemun M.M., Kavlak G., McNerney J., Buongiorno J., Trancik J.E. (2020). Sources of cost overrun in nuclear power plant construction call for a new approach to engineering design. Joule.

[53] Klemun M.M. (2020).

[54] Clarke L., Wei Y.-M., De La Vega Navarro A., Garg A., Hahmann A.N., Khennas S. (2022). Climate Change 2022: Mitigation of Climate Change.

[55] Allen C., Reid M., Thwaites J., Glover R., Kestin T. (2020). Assessing national progress and priorities for the Sustainable Development Goals (SDGs): experience from Australia. Sustain. Sci..

[56] Fu R., Feldman D., Margolis R., Woodhouse M., Ardani K. (2017).

[57] Razdan P., Garrett P. (2019). https://www.vestas.com/content/dam/vestas-com/global/en/sustainability/reports-and-ratings/lcas/LCA%5C%20of%5C%20Electricity%5C%20Production%5C%20from%5C%20an%5C%20onshore%5C%20V15042MW%5C%20Wind%5C%20PlantFinal.pdf.coredownload.inline.pdf.

[58] Nuclear Energy Agency and International Atomic Energy Agency. Measuring Employment Generated by the Nuclear Power Sector, p. 96. doi: https://doi.org/10.1787/9789264305960-en. url: https://www.oecd- ilibrary.org/content/publication/9789264305960-en

[59] United Engineers & Constructors (1986).

[60] (2021). ACE Ultra-clean Biomass Cookstove User Manual.

[61] Zhu L., Chen M. (2020). Research on spent LiFePO4 electric vehicle battery disposal and its life cycle inventory collection in China. Int. J. Environ. Res. Publ. Health.

[62] Bouckaert S., Pales A.F., McGlade C., Remme U., Wanner B., Varro L., D'Ambrosio D., Spencer T. (2021).

[63] Klemun M.M., Trancik J.E. (2019). Timelines for mitigating the methane impacts of using natural gas for carbon dioxide abatement. Environ. Res. Lett..

[64] Most Dangerous Industries: Industry Incidence and Rates. Tech. rep. National Safety Council. https://injuryfacts.%20nsc.org/work/industry-incidence-rates/most-dangerousindustries/.

[66] Sovacool B.K., Gilbert A., Nugent D. (2014). Risk, innovation, electricity infrastructure and construction cost overruns: Testing six hypotheses. Energy.

[67] Sovacool B.K., Gilbert A., Nugent D. (2014). An international comparative assessment of construction cost overruns for electricity infrastructure. Energy Research & Social Science.

