# Supplementary material for: Toward evaluating the effect of technology choices on linkages between sustainable development goals
Source: iScience. 2022 Dec 21;26(2):105727. doi: 10.1016/j.isci.2022.105727 (PMC9869479; doi:10.1016/j.isci.2022.105727)
Supplement: Document S1. Figures S1–S3 and Tables S1–S47 [file mmc1.pdf]

iScience, Volume *26*

Supplemental information

Toward evaluating the effect of technology choices on linkages between sustainable development goals

Magdalena M. Klemun, Sanna Ojanperä, and Amy Schweikert

Supplemental Information: Towards evaluating the effect of technology choices on linkages between sustainable development goals

Magdalena M. Klemun[a,d,**], Sanna Ojanperä[b,e], Amy Schweikert[c]

[a]*Division of Public Policy and Energy Institute, The Hong Kong University of Science and Technology, Clear Water Bay, Hong Kong*
[b]*Oxford Internet Institute, University of Oxford, Oxford, UK*
[c]*Department of Mechanical Engineering, Colorado School of Mines, Golden, Colorado, USA*
[d]*Institute for Data, Systems and Society, Massachusetts Institute of Technology, Cambridge, MA, USA*
[e]*The Alan Turing Institute, London, UK*

Here we discuss industry-indicator linkages separately for each SDG. Tables S-16-S-47 give the data sources and references used to assign a value ('1' for desirable change, '-1' for undesirable change) to each industry-indicator matrix element. Each table covers one technology and one SDG, with the assigned value for each potential link listed by SDG indicator. The explanations in the tables are short and designed for quick reference. Details on individual calculations are given in the following sections.

S-1. SDG1: No Poverty

S-1.1. Indicators 1.1.1, 1.2.1, 1.2.2

Technology manufacturing and deployment can reduce poverty through jobs creation.[1,2] This relationship can affect indicators 1.1.1 ('Proportion of population below the international poverty line, by sex, age, employment status and geographical location (urban/rural)'), 1.2.1 ('1.2.1 Proportion of population living below the national poverty line, by sex and age'), and 1.2.2 (Proportion of men, women and children of all ages living in poverty in all its dimensions according to national definitions). We treat these indicators equally due to their similarity. We assign a '1' to all low-tech industries in a technology's network based on the assumption that jobs lifting people above the poverty line will tend to be low-skilled jobs in industries outside the medium- and high-tech industry cluster as defined by the UN.[3] We therefore assign a '1' to all industries except for ISIC Rev. 4 codes 20 (Manufacture of chemicals and chemical products), 26 (Manufacture of computer, electronic and optical products), 27 (Manufacture of electrical equipment), and 28 (Manufacture of machinery and equipment n.e.c.), which are the industries listed in[3] as medium-high-tech that also appear in the technology networks developed here.

We assign a '1' to all power generation industries (221113, Solar/nuclear electric power generation; 221115, Wind electric power generation) because of documented relationships between electricity consumption per capita and economic development indicators such as the human development index, for example.[4] It should be noted, however, that the empirical evidence on linkages between electricity access and poverty is mixed, with some studies finding no consistent pattern of energy-driven poverty reduction (e.g., in Kenya[5]) or weak impacts on standard poverty indicators (e.g., in Rwanda[6]). We neglect these contradictions since a '1' in our framework only indicates a potential linkage, not a predetermined relationship. Future work could evaluate the presence and strength of linkages in the context of specific countries' technology markets.

S-1.2. Indicator 1.4.1

We assign a '1' to all power generation industries (221113, Solar/nuclear electric power generation; 221115, Wind electric power generation) since power generation is the industry in the set of industries covered here that is most directly related to indicator 1.4.1 ('Proportion of population living in households with access to basic services (water, electricity, sanitation, clean cooking fuels)). For cookstoves, we assign a '1' to the linkage between cookstove sellers and indicator 1.4.1 as a placeholder for cooking, the basic service provided by this technology, which is not listed in the NAICS code. Using industry 423620 (Household Appliances, Electric Housewares, and Consumer Electronics Merchant Wholesalers) instead allows us to treat all technologies consistently in the sense that the final service provided is connected to indicator 1.4.1.

S-1.3. Other SDG1 indicators

We do not identify any clear linkages between technology industries and other indicators of SDG1. Government spending on poverty reduction programmes (indicator 1a1, Proportion of resources allocated by the government directly to poverty reduction programmes), essential services (indicator 1a2, Proportion of total government spending on essential services (education, health and social protection)), as well as women and other marginalized groups (1.b.1, Proportion of government recurrent and capital spending to sectors that disproportionately benefit women, the poor and vulnerable groups) can be indirectly associated with

*Correspondence: magdalena@ust.hk
**Lead Contact

industrial development and technology deployment if the same programs support both. However, the link is not functional in the sense that poverty reduction programs (e.g., to reduce hunger) do not necessarily require energy technology investments. In addition, no central repository on development programs and their focal points exist, as would be needed to compare industries and services in terms of their simultaneous focus on industrial development.

For indicator 1.3.1 ('Proportion of population covered by social protection floors/systems, by sex, distinguishing children, unemployed persons, older persons, persons with disabilities, pregnant women, newborns, work- injury victims and the poor and the vulnerable'), data would be required on an industry-by-industry basis on the level and value of insurance provided to employees in order to assess differences between technologies. Since this data is not available to our knowledge, we do not assume any potential linkages for indicator 1.3.1.

For the two disaster-related indicators 1.5.1 ('Number of deaths, missing persons and persons affected by disaster per 100,000 people') and 1.5.2 ('Direct disaster economic loss in relation to global gross domestic product (GDP)'), we assign a '1' to the link to the power generation industries in the respective technology networks (221113, Solar/nuclear electric power generation; 221115, Wind electric power generation). This choice is motivated by the importance of electricity access for disseminating early warning signals and avoid deaths, and for locating missing persons in the aftermath of disasters.[7,8]

The two remaining SDG1 indicators appear largely unrelated to technology industries, including 1.4.2 ('Proportion of total adult population with secure tenure rights to land, with legally recognized documentation and who perceive their rights to land as secure, by sex and by type of tenure') and 1.5.3 ('1.5.3 Number of countries with national and local disaster risk reduction strategies').

S-2. SDG2: Zero Hunger

Our review did not identify direct linkages between technology manufacturing and deployment and SDG2 indicators. Malnourishment (indicators 2.1.1, Prevalence of undernourishment; 2.1.2, prevalence of moderate or severe food insecurity in the population, based on the Food Insecurity Experience Scale (FIES); 2.2.1, prevalence of stunting among children under 5 years of age; 2.2.2, prevalence of malnutrition among children under 5 years of age, by type (wasting and overweight)) can only be reduced if the additional income from jobs in technology industries can be spent on food. However, this potential link is not functional because technology industries or services are not strictly required to reduce malnutrition, and food supply may not be available (e.g., due to shortages) even if disposable income is.

Similarly, energy technology industries and services are largely unrelated to productivity in agriculture (indicator 2.3.1, is the volume of production per labour unit by classes of farming/pastoral/forestry enterprise size) and food producer income levels (indicator 2.3.2, average income of small-scale food producers, by sex and indigenous status), as well as to agricultural practices (2.4.1, the proportion of agricultural area under productive and sustainable agriculture), genetic diversity in agriculture (2.5.1, number of plant and animal genetic resources for food and agriculture secured in either medium- or longterm conservation facilities) and extinction levels (2.5.2, proportion of local breeds classified as being at risk, not at risk or at unknown level of risk of extinction) and government expenditures on agriculture (2.a.1, 2.a.2).

S-3. SDG3: Good Health and Well-Being

S-3.1. Indicator 3.9.1

The only indicator for which we expect direct effects from technology investment is indicator 3.9.1 ('Mortality rate attributed to household and ambient air pollution'). Specifically, we assign a '1' to all power generation industries in the solar PV, wind, and nuclear fission technology network because they cause lower per-kWh particulate matter emissions than most fossil-fired power plants.[9] For other industries, we assign a '-1' to all industries covered in the U.S. Environmental Protection Agency's 2017 National Emissions Inventory,[10] which provides particulate matter emissions factors for all major industries. We use the US data as a proxy for industry particulate matter emissions globally. While emissions factors may differ across locations, the high-level fact that some industries have higher potential to cause PM emissions than others is rooted in fundamental industry characteristics such as fuels and combustion processes employed. We therefore do not conduct a detailed review of country-level emissions factors. The relevant sectors covered by EPA's inventory and the matching industries in the NAICS-code for which we assign a '1' are listed below. We treat these industries equally for all technologies in our set.

- 'Agriculture' (crops and livestock dust, fertilizer application, and livestock waste): 113210, 115112
- 'Dust' (construction dust, paved road dust): 237130
- 'Industrial processes' (cement manufacturing, chemical manufacturing, ferrous metals, mining, non-ferrous metals, storage and transfer): 212322, 212299, 212230, 212291, 325211, 327211, 331314, 331313, 331110, 212291, 331210, 332111, 332410, 332313, 332312, 332811, 332911, 327320, 212325, 325199
- 'Mobile' (commercial marine vessels, on-road diesel and gasoline vehicles): 483111, 484122

S-3.2. Other SDG3 indicators

Several SDG3 indicators related to maternal health, child mortality, cardiovascular disease and cancer, could be indirectly affected by changes in air pollution. For instance, improvements in indicator 3.9.1 may lead to improvements

in these indicators at a later point in time. However, our work focuses on functional linkages and direct relationships. We therefore assign a '0' to linkages between technology industries and the above described health indicators.

As for the remaining indicators, we do not identify a functional connection between air pollution caused by technology industries and indicators related to malaria, HIV, and tuberculosis, or alcohol consumption, suicide rates, road traffic accidents, tobacco use, mortality rate due to poisoning and unsafe water, access to contraception, and household level health expenditures. We therefore assign a '0' to these indicators.

S-4. SDG4: Quality Education

It is clear that energy technology deployment (if not strictly limited to industry use) can increase a population's electricity access, and better electricity access in turn can support better education through electric lighting and computers. Freeing up time for education that was previously used for fuel collection (e.g.,[11,12]) is another mechanisms through which better electricity access can support education goals. However, since here we focus on direct, functional linkages, we assign a '0' to the linkages between technology industries and services and the indicators of SDG4.

S-5. SDG5: Gender Equality

Most SDG5 indicators are development metrics not directly affected by energy technology investments, including violence against women (indicators 5.2.1, 5.2.2), child marriage (indicator 5.3.1), and access to reproductive care (indicators 5.6.1, 5.6.2). The only indicator that could be directly affected by energy technology choices and the expansion of related industries is indicator 5.5.2, women in managerial positions, due to differences across technology industries and services in terms of how frequently women assume leadership positions. We use data from the International Labor Organisation[13] to group sectors into those with a above-average and below-average share of women in managerial positions. The grouping is based on weighted averages of data from 14 countries for which recent data is available (Argentina, Brazil, France, Italy, Mexico, Russian Federation, Rwanda, Spain, South Korea, Switzerland, Turkey, United Arab Emirates, United Kingdom, United States, see Figure 2 in[13] for details). Given an average share of 38% women in managerial positions, there are 10 sectors with an above average share, and 10 sectors with a below average share (see below). We assign a '1' to the former and a '-1' to the latter group of industries in the respective technology networks.

- **Industries with an above-average share of women in managerial positions:** Human health and social service activities; Education; Activities of households as employers; Other service activities; Arts, entertainment, and recreation; Financial and insurance activities; Public administration and defence; Accommodation and food service activities; Professional, scientific, and technical activities; Real estate activities;
- **Industries with a below-average share of women in managerial positions:** Administrative and support service activities; wholesale and retail trade; Transportation and storage; Manufacturing; Agriculture, forestry and fishery; Electricity, gas, steam, and air conditioning supply; Water supply, sewerage, waste management; Mining and quarrying; construction.

We map the sectors above to industries in the NAICS code. For most industries in our technology-industry networks, the matching is clear (e.g., because these industries fall under 'Manufacturing'). For other industries, we do the mapping based on a review of industry category definitions. For instance, we group 'Specialty Trade Contractors' (NAICS) under 'Construction' (ILO grouping) and 'Professional Services' (NAICS) under 'Professional, technical, and scientific activities' (ILO).

S-6. SDG6: Clean Water and Sanitation

S-6.1. Indicator 6.4.1: Water use efficiency

We assign a '1' to all service industries and a '-1' to all manufacturing industries based on a review of currently available water use efficiency data. As shown in the 'Step-by-step monitoring methodology for SDG Indicator 6.4.1'[14] for the Netherlands (p. 15, Table 1), service water use efficiency (435.5 USD/m^3) is more than a factor 30 (a factor 36) greater than industrial water use efficiency (12.0 USD/m^3).[14] While this applies to one country only, the difference between the two industry efficiencies is large enough to assume that investments in service industries may have more potential to increase a country's water use efficiency, while investments in manufacturing industries may have more potential to decrease a country's water use efficiency. In 2018, global average water use efficiency was 43.4 USD/m^3,[15] with 93 out of a 163 countries for which data was available in 2018 exhibiting an average water use efficiency greater than the industrial water use efficiency in the Netherlands. All except one countries' water use efficiency was smaller than the service industry's water use efficiency in the Netherlands (see Fig S-1 for country water use efficiencies). (The country with the highest water use efficiency in 2018, Luxembourg, has reported a value of 1096.77 USD/m^3, which is the only country with a water use efficiency greater than that of service industries in the Netherlands. The next country, Switzerland, has a water use efficiency of 339 USD/m^3, which is still 22% smaller than the service industry's water use efficiency in the Netherlands.) The overall global range of water use efficiencies in 2018 is 0.20 (Somalia) to 1096.77 USD/m^3 (Luxembourg). For countries not included in the set of above-industry water use efficiency countries, the linkage value for technology

industries should be changed from '-1' (inducing lower water use efficiency relative to national average) to '1' (higher water use efficiency than the national average). These countries include the following (listed from highest to lowest water use efficiency in 2018): Peru, Burkina Faso, Kenya, Gambia, Georgia, Albania, El Salvador, Tunisia, North Macedonia, Chad, Ecuador, Guinea, Jamaica, Morocco, Sierra Leone, Bulgaria, Dominican Republic, Republic of Moldova, Mozambique, Venezuela, Sao Tome and Principe, Cambodia, United Republic of Tanzania, Senegal, Thailand, Eritrea, Kazakhstan, Suriname, Nicaragua, Ukraine, Bhutan, Burundi, Bangladesh, Sri Lanka, Serbia, Iraq, Yemen, , Haiti, Ethiopia, Zimbabwe, Liberia, Egypt, Iran, Libya, Indonesia, Mauritania, Azerbaijan, Niger, Eswatini, Armenia, Philippines, Malawi, Guinea-Bissau, India, Sudan, Vietnam, Chile, Timor-Leste, Nepal, Guyana, Lao People's Democratic Republic, Mali, Myanmar, Democratic People's Republic of Korea, Pakistan, Turkmenistan, Uzbekistan, and Syria.

S-6.1.1. Technology-specific water use efficiencies

Photovoltaic systems. We use a median estimate of PV's life cycle water consumption from the literature (94 gallons per MWh in total[16]) and a global average LCOE value for 2019 from the International Energy Agency (89 2017 USD/MWh[17]). This results in an estimated water use efficiency of 210 (210.10) USD/m^3.

Wind turbines. We take a median estimate of life cycle water consumption and withdrawal for onshore wind power generation from the literature (26 gallons per MWh in total[16]). We then use a global weighted average LCOE for wind power from the International Energy Agency (52.6 USD/MWh in 2019[18]) to compute the water use efficiency of wind power generation as the product of life-cycle water use and levelized electricity costs. The result is 515 (514.65) USD/m^3.

Nuclear fission. We use a median estimate of life-cycle water consumption from a harmonization of previous literature estimates (780 gallons/MWh[16]), and an LCOE value of USD 163/MWh.[19] These assumptions result in a water use efficiency of 55 (55.21) USD/m^3. We therefore assign a '1' (potential for positive linkage) to all countries with a water use efficiency smaller than 55 (the majority of countries, see Fig. S-1), and a '-1' to all countries with a water use efficiency greater than 55.

S-7. SDG9

S-7.1. Indicator 9.3.1: Proportion of small-scale industries in total industry value added

As we explain in Table S-37, small-scale in industries are defined by the UN as enterprises with 5-19 employees. While the prevalence of these small-scale enterprises in different sectors depends on the country, there is also a general trend across countries. Based on 2017 OECD data on the number of enterprises by size and sector, the service sector tends to have a significantly larger number of enterprises with fewer than 19 employees than the construction and manufacturing sectors, respectively.[20] (Note that we use the number of companies with fewer than 19 employees as a proxy since data on companies with 5-19 employees is not available for several countries.) For example, the United States, the United Kingdom, France, Italy, Japan, Brazil, the Netherlands, and Germany had 11 (5.5), 12.5 (5.2), 10.2 (4), 7.4 (5.2), 5.5 (4.5), 9.8 (27), 14.3 (5.3), and 10.3 (5.6) times as many small-scale enterprises in the service sector, respectively, than in the manufacturing (construction) sector (see Table 2.1 in[20] for the employee numbers for each country). Among OECD countries, no country has more small-scale industries in the manufacturing or construction industry than in the service sector. Standardized data for non-OECD countries is scarcely available, but those where data is available show similar results. In South Africa, for example, approximately 70% of small and medium sized enterprises are in the services sector, compared to 15%, 9%, and less than 1% in construction, manufacturing and mining, respectively (2018 data, Table 6 in[21]). Based on this, we assign a '1' to all service industries in our technology-industry networks, and a '0' to all construction and manufacturing industries.

S-7.2. Indicator 9.5.1: Research and development expenditure as a proportion of GDP

We consider the R&D intensity of different industries based on R&D expenditure per unit of gross value added, using data provided by the OECD.[22] Industries classified as high- and medium-high-R&D intensity exhibit a range of 24-32% and 6-19% R&D as a % of gross value added, respectively (data is from 2018; see Table 1 in[22]). In comparison, the R&D intensity of all countries for which data was available in 2018 is below 5%[23] (see Fig. S-2 for a selection of countries that covers the current range of global R&D intensities). We therefore assign a '1' to all high- and medium-high R&D-intensity industries for all countries. This assignment is based on the assumption that investment in any industry with a higher-than-average R&D intensity has the potential to increase a country's overall R&D intensity, all else equal.

For the next lower industry category, medium-R&D intensity, we assign a '1' only to selected industries for countries with less than 1.9 % RD spending as a % of gross value added. The list of these countries includes all countries for which data was available in 2018,[23] except Austria, Belgium, China, Czech Republic, Denmark, Finland, France, Germany, Israel, Japan, the Republic of Korea, the Netherlands, Norway, Singapore, Slovenia, Sweden, Switzerland, and the U.S. The cut-off is based on the % spending in the medium R&D intensity industry with the lowest R&D spending in this category (Repair and installation of machinery and equipment, 1.93 % RD spending as a % of gross value added). Other medium-R&D-intensity industries include 'Other non-metallic industry products'(R&D-intensity of 2.24%), 'Other manufacturing'(R&D-intensity of 2.85%), and 'Basic metals' (R&D-intensity of 2.07%).[22]

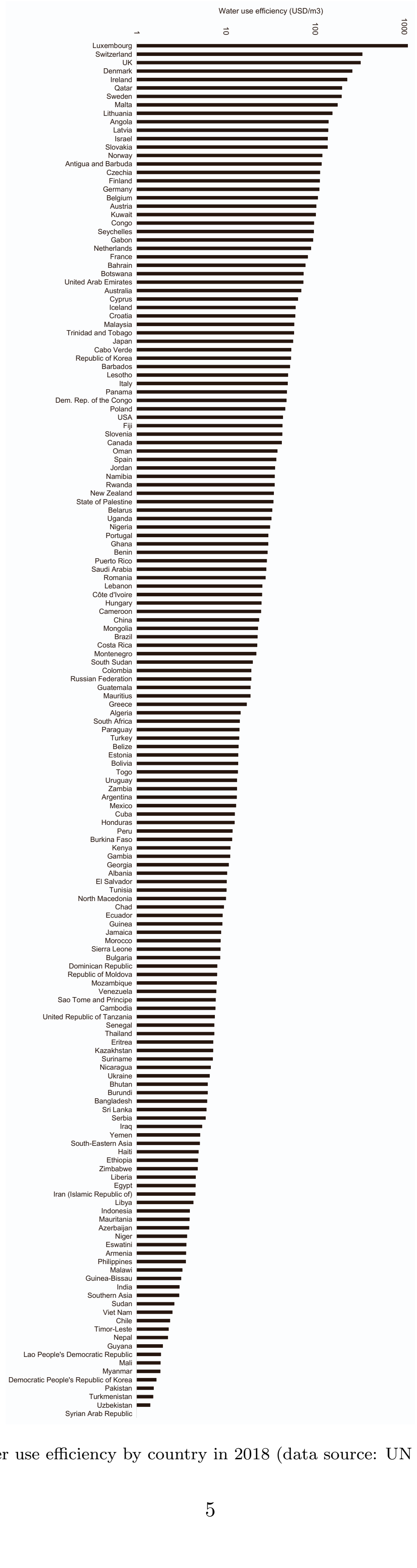

Figure S-1: National average water use efficiency by country in 2018 (data source: UN SDG Database, indicator 6.4.1[15]).

S-7.3. *Indicator 9.5.2: Researchers per million inhabitants*

Our review points to a correlation between countries' R&D-intensity and the number of researchers per million inhabitants. E.g., the Republic of Korea, Japan, Switzerland, Sweden, Denmark, Germany, the Netherlands, Finland, Norway, and Israel all have 4000 or more researchers per million inhabitants[23,24] (see Fig. S-3), putting them among the top 10 countries globally. The same countries are also among the top countries in terms of R&D expenditure in % of GDP[23] (see Fig. S-2). For this reason, and since industry-specific data on researcher employment is not available, we use the same assignments for indicators 9.5.1 and 9.5.2.

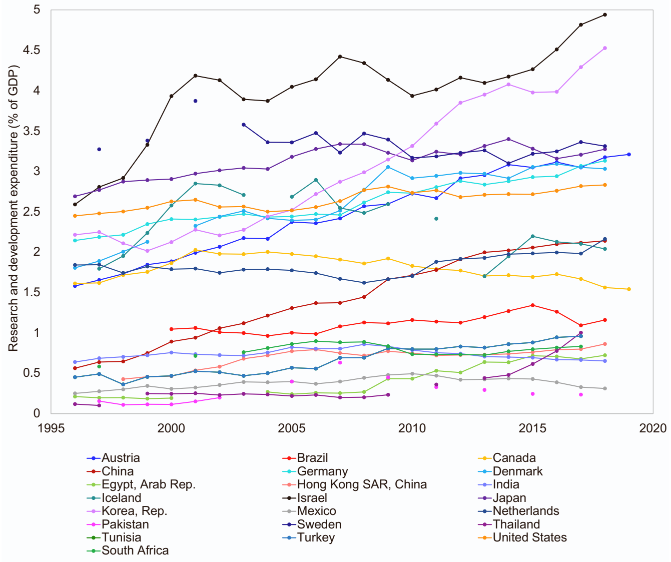

Figure S-2: Countries by R&D intensity, where R&D intensity is measured as R&D expenditure in % of GDP (data source: World Bank[23]).

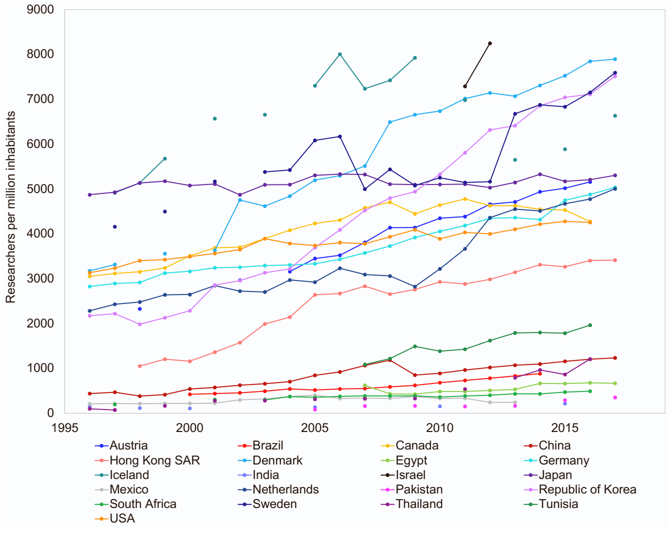

Figure S-3: Countries by researcher density in researchers per million inhabitants (data source: UNESCO[24]).

S-7.4. *Indicator 9.A.1: Official international support for infrastructure*

According to OECD data and UNCTAD calculations,[25] most economic sectors and related industries receive official international support (i.e., industries can't be grouped into those that do not receive support and those that do). Sectors receiving international support and supplying energy technology components or services include Transport and Storage (approximately 12% of official international support over the 2016-2019 period), Energy (11%), Banking (7%), Business and other services (2%), as well as Industry, Mining and Construction (5%).[25] Based on this, we assign a '1' to all industries and services since any investment in an energy technology could lead to additional international support for any required component or service industry. (Note that this indicator simply measures total flows, so we do not need to account for a change relative to baseline international support for infrastructure.)

S-8. SDG10: Reduce inequality within and among countries

S-8.1. *Indicators 10.1.1 and 10.1.2*

We treat indicator 10.1.1 (Growth rates of household expenditure or income per capita among the bottom 40 per cent of the population and the total population) and 10.1.2 (Proportion of people living below 50 per cent of median income, by sex, age and persons with disabilities) in the same way as indicator 1.1.1 (Proportion of population below the international poverty line, by sex, age, employment status and geographical location (urban/rural)). The main rationale is that jobs that lift people above the poverty line, like jobs that show potential to increase the income of the bottom 40% of the population or the lower 50% of the median incom) are more likely to be low-skilled jobs outside the high- and medium-high -tech industry. We therefore assign a '1' for all industries and services not considered high-tech (also see goal 9.B.1) as defined by the UN.[26]

S-8.2. *Indicators 10.b.1*

We assign a '1' to all linkages between technology industries and services and indicator 10.b.1 (Total resource flows for development, by recipient and donor countries and type of flow). The rationale is that investments in all industries and services can contribute to official development flows since no industry or service is by definition excluded from development assistance.

S-8.3. *Other indicators*

We did not find evidence for linkages between the energy technology industries and services covered here and other indicators of SDG10. For indicators related to discrimination and representation (10.3.1 (proportion of population reporting having personally felt discriminated against or harassed in the previous 12 months on the basis of a ground of discrimination prohibited under international human rights law) and 10.6.1 (Proportion of members and voting rights

of developing countries in international organizations), it is not possible to draw a connection to industries or services. For indicator 10.3.1, this is the case because there is no data available on discrimination rates by industry. For indicator 10.6.1, there is no direct connection between growth in technology industries and representations in international organizations. For example, investment in technology manufacturing and service industries to scale photovoltaics may correlate with progress towards the OECD's membership criteria (e.g, open, transparent and free-market economies), but that investment would need to be paired with poltical and economic reforms as technology industries per se do not require transparent economies. Similar points about indirect connections rather than functional connections can be made about indicators related to financial markets (10.5.1, financial soundness indicator).

We also do not find any evidence or rationale for a potential linkage between industries and indicators related to migration (10.7.1 to 10.7.4). Distributed energy technologies may contribute to improving the conditions at migrant shelters by improving electricity access, thereby potentially improving indicator 10.7.3 (number of people who died or disappeared in the process of migration towards an international destination), but the effects are likely small compared to those of policy decisions and availability of public and private funds to better manage migration and border crises.

S-9. SDG11: Make cities inclusive, safe, resilient and sustainable

S-9.1. Indicators 11.1.1-11.4.1

Among the indicators related to sustainable development in cities, most do not appear to be directly influenced by growth of technology-related industries or services. Low-cost, distributed energy technologies such as rooftop PV may improve the living conditions in slums by providing electricity, but that does not imply changes in the proportion of urban populations living in slums overall (indicator 11.1.1, proportion of urban population living in slums, informal settlements or inadequate housing'). Low-cost availability of low-carbon technologies may simultaneously ease the transition to low-carbon transportation systems, yet that may not mean that a larger share of the population has access to transportation systems (indicator 11.2.1, 'Proportion of population that has convenient access to public transport, by sex, age and persons with disabilities'). We therefore do not assume any potential for linkages between technology-related industries and services and these two indicators.

Similarly, it is highly uncertain how growth in technology-related industries and services might affect land consumption rates of urban agglomerates (indicator 11.3.1, 'ratio of land consumption rate to population growth rate'), participatory planning (indicator 11.3.2, 'proportion of cities with a direct participation structure of civil society in urban planning and management that operate regularly and democratically'), and investments in the protection of natural and cultural heritage (indicator 11.4.1, 'Total expenditure (public and private) per capita spent on the preservation, protection and conservation of all cultural and natural heritage'). For indicator 11.3.1 specifically, it should be noted that land consumption here is defined as land consumption by urban agglomerates,[27] not by power plants or technologies more broadly. We therefore do not use the land consumption of industries as the basis for assessing potential linkages. (A connection between growing technology industries and services is likely only if industry growth displaces rural populations and if these populations as a consequence migrate to urban areas while also increasing the land consumed by these areas, rather than just increasing population density.)

S-9.2. Indicators related to natural disasters (11.5.1, 11.5.2, 11.B.1., 11.B.2

Although low-carbon electricity technologies may provide electricity access during or immediately following natural disasters, it is not clear that this would contribute to a reduction in deaths of missing persons (11.5.1), or reduce direct economic losses (11.5.2). Note that direct economic losses measures the monetary value of total or partial destruction of physical assets in the area affected by a disasters, which means that investments in energy technologies could also have a negative effect on losses (i.e., increase losses) if they make local assets more valuable. Due to these uncertainties, we do not assume that there exists potential for the technology industries and services in our network to affect indicators related to natural disasters.

S-9.3. Other SDG11 indicators

We did not find empirical or other reasons to assume that energy technologies directly affect urban solid waste management (indicator 11.6.1, 'Proportion of urban solid waste regularly collected and with adequate final discharge out of total urban solid waste generated, by cities'), or the availability of open and safe spaces in cities (11.7.1-11.7.2). To influence indicator 11.7.1 ('Average share of the built-up area of cities that is open space for public use for all, by sex, age and persons with disabilities'), specifically, energy technologies would need to be constructed within already built-up areas in cities that are also of interest for public use. Deployment of energy technologies in urban areas will likely occur on rooftops or in industrial parks, however, and is thus unlikely to affect this indicator.

For urban air pollution, (indicator 11.6.1, 'Annual mean levels of fine particulate matter (e.g. PM2.5 and PM10) in cities (population weighted)), we use the same rationale as for indicator 3.9.1 (Mortality rate attributed to household and ambient air pollution) since mean particulate matter levels and mortality rates have been shown to correlate.[28,29,30] See SI section S-3 for details.

For urban planning (indicator 11.A.1, 'Proportion of population living in cities that implement urban and regional development plans integrating population projections and resource needs, by size of city') our review also did not point to a direct relationship with growth in technology-related industries and services. If a larger fraction of the

overall population lives in cities with development plans, this development may correlate with growth in technology industries if these plans cover energy technologies and induce growth in energy technology markets. Due to these required conditions, and the direction of the effect (from indicator to industries rather than the other way around), we assign a '0' to all linkages between industries and indicator 11.A.1.

For sustainable buildings (indicator 11.C.1, 'Proportion of financial support to the least developed countries that is allocated to the construction and retrofitting of sustainable, resilient and resource-efficient buildings utilizing local materials'), we distinguish between energy technologies that can realistically be deployed in sustainable buildings (rooftop PV, wind turbines) given their flexible scale and because they have already been used in this context in the past, and all other energy technologies in our set. For the former, we assign a '1' to all industries and services, because market growth and associated cost reductions has the potential to increase the proportion of financial support dedicated to sustainable buildings.

S-10. SDG12: Ensure sustainable consumption and production patterns

Several SDG12 indicators focus on policies and monitoring activities to promote sustainable consumption. Such policies and activities may correlate with growth in clean energy industries, but we did not find any evidence for direct, functional linkages between technology-related industries and services and indicators related to the prevalence of national-level or global sustainability policies (12.1.1, 'Number of countries with sustainable consumption and production (SCP) national action plans'; 12.4.1, 'Number of parties to international multilateral environmental agreements on hazardous waste'; 12.7.1, 'Number of countries implementing sustainable public procurement policies and action plans'; 12.8.1, 'Extent to which global citizenship education and education for sustainable development (including climate change education) are mainstreamed') or monitoring activities (12.6.1, 'Number of companies publishing sustainability reports').

For the indicator focused specifically on sustainable technologies (12.A.1, 'Amount of support to developing countries on research and development for sustainable consumption and production and environmentally sound technologies'), we use the UNEP's definition of environmentally sound technologies, which includes renewable energy technologies and waste management technologies.[31] We therefore assign a '1' to all linkages between technology industries required for solar PV and wind and indicator 12.A.1, and a '0' for linkages between clean cookstoves and nuclear fission and the same indicator.

S-10.1. Indicators related to materials usage and food loss

Indicators 12.2.1 ('Material footprint, material footprint per capita, and material footprint per GDP) and 12.2.2 ('Domestic material consumption, domestic material consumption per capita, and domestic material consumption per GDP') are the same as indicators 8.4.1 and 8.4.2. Please refer to Tables S-27 (PV), S-31 (wind), S-33 (nuclear fission), and S-34-S-35 (cookstoves) for details.

We did not find evidence supporting a potential linkage between technology-related industries and services and the rate of global food waste production because energy technology industries do not generate food waste.

S-10.2. Indicators related to waste generation

For indicator 12.4.1 ('Hazardous waste generated per capita and proportion of hazardous waste treated, by type of treatment'), we use the European Union's list of waste as referred to in Article 7 of directive 2008/98/EC[32] to identify technology industries that show potential to influence indicator 12.4.1 negatively because they have generated hazardous wastes in the past. In the EU's list, these industries are marked with an asterisk. We assign a '-1' to all technology industries that match the EU's waste categories at the six-digit level. As a result, all industries that fall into the NAICS categories mining, basic metals, and manufacturing (including metals, glass, plastics, electronic products, and machinery) show potential to influence indicator 12.4.1 negatively. The waste produced by contractors and other technology-related service industries, on the other hand, are not listed in,[32] and we therefore assign a '0' to all the cells linking them to indicator 12.4.1.

For indicator 12.5.1 ('National recycling rate, tons of material recycled out of total waste generated'), we use region-specific municipal solid waste recycling rates as a starting point to determine whether an industry is likely to influence the national recycling rate positively or negatively. Municipal solid waste tends to constitute the majority of waste generated by mass. We therefore assume that the national recycling rate is most strongly influenced by municipal solid waste recycling rates. These rates range between 32.1% in the U.S. (data from 2018[33]) and 48% in the EU (data from 2020[34]) to 5-20% in China (https://waste-management-world.com/recycling/recycling-in-china-from-zero-to-hero/).

In comparison to municipal solid waste, metals are recycled at significantly higher rates. In particular, several metals have achieved recycling rates of 50% or greater, including Ti, Cr, Mn, Fe, Co, Ni, Cu, Zn, Nb, Rh, Pd, and Ag.[35] We therefore assign a '1' to linkages between copper and iron mining, processing and manufacturing industries, since all these industries contribute to the production of metals with higher recycling rates than the national average in the U.S., EU, and China. We also assign a '1' to aluminum-related industries and industries manufacturing electronics because recycling rates tend to be higher.[36]

S-11. SDG13: Take urgent action to combat climate change and its impacts

S-11.1. Indicator 13.1.1

Energy technologies can help 'strengthen resilience and adaptive capacity to climate-related hazards and natural

disasters in all countries', which is one of the sub-goals of SDG13. Distributed renewable generation could, particularly if combined with storage, reduce the number of people affected by consequences of natural disasters, for example. However, the potential linkages between energy technology manufacturing and services are not captured in the current indicator framework. Indicator 13.1.1 ('Number of deaths, missing persons and directly affected persons attributed to disasters per 100,000 population') only accounts for direct effects of disasters and not for indirect economic effects of, for example, disaster-related power outages. We discuss this topic in section 3.6.6 in the main text.

S-11.2. Indicators related to risk management, planning, and education

Our review did not point to evidence for direct connection between energy technology investments and the prevalence of national and local disaster management strategies (indicators 13.1.2, 'Number of countries that adopt and implement national disaster risk reduction strategies in line with the Sendai Framework for Disaster Risk Reduction 2015â2030' and 13.1.3, 'Proportion of local governments that adopt and implement local disaster risk reduction strategies in line with national disaster risk reduction strategies'). Investments in climate change mitigation (and thus in energy technologies) may correlate with greater resource availability for adaptation, but energy technologies do not directly affect governments' planning decisions.

Regarding the integration of climate change into national policy and education (indicators 13.2.1, 'Number of countries that have communicated the establishment or operationalization of an integrated policy/strategy/plan which increases their ability to adapt to the adverse impacts of climate change and foster climate resilience and low greenhouse gas emissions development', and 13.3.1, 'Number of countries that have integrated mitigation, adaptation, impact reduction and early warning into primary, secondary and tertiary curricula'), there may be indirect effects of and correlations with technology investments, but we did not find evidence for direct effects. Investments in low-carbon energy technologies will, if displacing high-carbon energy supply, simultaneously support low greenhouse gas emissions development as mentioned in the definition of indicator 13.2.1. At the same time, economies of scale, learning-by-doing and other phenomena emerging from these investments may make these technologies cheaper and prompt further investment. However, that does not necessarily affect the communication or establishment of climate strategies. Similarly, education will only be affected if increasing prevalence of energy technologies motivates more emphasis on climate change in curricula, but one can also happen without the other.

S-11.3. Indicators related to development finance

Technology investments may correlate with changes in development finance indicators if these investments are supported by the Green Climate Fund (indicator 13.A.1, 'mobilized amount of United States dollars per year between 2020 and 2025 accountable towards the 100 billion commitment') or other types of development funding (indicator 13.B.1, 'number of least developed countries and small island developing States that are receiving specialized support, and amount of support, including finance, technology and capacity-building, for mechanisms for raising capacities for effective climate change-related planning and management'). Technology investments can be made independent of development funding, however. There is thus no functional link, with the exception of special circumstances in failed or heavily indebted countries that aren't the main focus here.

S-12. SDG14: Conserve and sustainably use the oceans, seas and marine resources

S-12.1. Indicator 14.1.1

Indicator 14.1.1 is the 'index of coastal eutrophication and floating plastic debris density'. Since none of the industries in our technology-industry networks are significant plastic polluters, we focus our review on potential industry contributions to coastal eutrophication due to NO_x emissions. We assign a '-1' to industries that are significant NO_x emitters based on the EPA national emissions inventory,[10] including industries using on-road Diesel vehicles (General freight trucking and Deep sea freight transportation in the NAICS system, 3-digit level), cement manufacturing, chemical manufacturing, mining, ferrous metals, non-ferrous metals, storage and transfer ('Warehousing and storage' in the NAICS system), and construction dust ('Power line construction' in the NAICS system). We note that direct discharge of NO_x-pollutants into coastal waters can also cause eutrophication. However, most of the larger sources of industrial nutrient pollution (pulp and paper mills, food and meat processing, agro-industries, and direct discharge of sewage from maritime vessels) are not included in our technology networks. (For maritime vessels we already assign a '-1' due to NO_x emissions, and note here that direct sewage discharge can contribute additionally to eutrophication.)

S-12.2. Indicator 14.3.1

Indicator 14.3.1 is the 'average marine acidity (pH) measured at agreed suite of representative sampling stations'. Since CO_2 emissions are the main cause of ocean acidification, we treat indicator 14.3.1 in the same way as indicator 9.4.1 (CO_2 emissions per value added, see SI Table S-38).

S-12.3. Other SDG14 indicators

We did not find evidence for a potential link between energy technology investments and other indicators of SDG14. For indicators related to the environmental management and regulation of maritime zones (indicators 14.5.1, 'coverage of protected areas in relation to marine areas', and 14.C.1, 'number of countries making progress in ratifying, accepting and implementing through legal, policy and institutional frameworks, ocean-related instruments that

implement international law, as reflected in the United Nations Convention on the Law of the Sea') or, more broadly, economic zones (indicator 14.2.1, 'proportion of national exclusive economic zones managed using ecosystem-based approaches'), there is no discernible direct effect of industries because investments in industry alone cannot be expected to prompt changes in practices of environmental regulation and environmental management.

We also did not find evidence for any of the industries and services in our list directly creating obstacles for the support of sustainable or small-scale fishing industries (indicators 14.4.1, 'Proportion of fish stocks within biologically sustainable levels', 14.7.1, 'sustainable fisheries as a proportion of GDP' and 14.B.1, 'Progress by countries in the degree of application of a legal/regulatory/policy/institutional framework which recognizes and protects access rights for small-scale fisheries') or the fight against illegal fishing (indicator 14.6.1., 'Progress by countries in the degree of implementation of international instruments aiming to combat illegal, unreported and unregulated fishing'). Governments deciding on a greater push for sustainability overall may invest in clean energy technologies and more sustainable fisheries at the same time, but energy technologies do not directly reduce overfishing or illegal fishing.

Lastly, all of the technologies in our set are land-based and we therefore do not find evidence pointing towards a possible increase in the share of R&D budgets available for marine technologies (indicator 14.A.1, 'proportion of total research budget allocated to research in the field of marine technology'). Moreover, 'marine technology' in the SDG context refers to instruments, equipment, vessels, processes and methodologies required to produce and use knowledge to improve the study and understanding of the nature and resources of the ocean and coastal areas,[37] rather than to technologies used in oceans. We therefore do not draw a connection between technology industries on our list and indicator 14.A.1.

S-13. SDG15: Sustainably manage forests, combat desertification, halt and reverse land degradation, halt biodiversity loss

S-13.1. Indicators related to forest coverage and desertification

In high-penetration scenarios, solar PV is projected to cover up to 5% of land in Europe, India, and other parts of Asia,[38] for example. Wind turbine pads, access roads, substations, and service buildings can also lead to significant direct land use.[39] These numbers suggest that there is potential for solar PV and wind deployment to negatively influence indicators related to forest coverage (15.1.1, 'Forest area as a proportion of total land area'; 15.2.1, 'Annual change in forest area', 'Biomass within the forest area', and 'Protection and maintenance of biodiversity'). Nuclear fission plants also involve direct land use for mining, milling, for the power plant itself and for waste processing and storage.[40] Clean cookstoves may negatively influence indicators 15.1.1. and 15.2.1 due to fuel wood consumption. We therefore assign a '-1' for all mining industries in the respective technology-industry network due to potential forest coverage losses after land clearing,[41] for power generation, and for agriculture industries (which only applies to clean cookstoves). We assign a '0' for all other industries.

For indicator 15.3.1 ('Proportion of land that is degraded over total land area'), the definition of 'degraded' covers factors that can be both positively and negatively influenced by energy technology investment. The United Nations Economic Commission for Europe (UNECE) defines land degradation as the reduction or loss of the biological or economic productivity and complexity of rain fed cropland, irrigated cropland, or range, pasture, forest and woodlands resulting from a combination of pressures, including land use and management practices.[42] While the biological productivity of land used by energy technologies can be reduced, economic productivity may be increased, and the net result will depend on the specific circumstances. We therefore do not assume that there is a potential for change in a positive or negative direction (the value in the corresponding industry-indicator cells is '0').

S-13.2. Indicators related to mountain biodiversity

Several factors indicate potential for linkages between energy technology investments and indicators related to mountain biodiversity (15.4.1, 'Coverage by protected areas of important sites for mountain biodiversity; 15.4.2, 'Mountain Green Cover Index'). In the context of SDG15, mountain areas are defined as either 'Mountain elevation 5' (an elevation of 1,000 to 1,500 meters and a slope greater than 5, or a local elevation range above the surrounding area of at least 300 meters for a 7 kilometer radius) or 'Mountain elevation 6' (an elevation of 300 to 1,000 meters and a local elevation range above the surrounding area of at least 300 meters for a 7 kilometer radius).[43] Although for solar PV most installations are located in agricultural areas, deserts, or grasslands, all of which have limited overlap with mountain terrain,[44] future scenarios of energy technology deployment under climate goals anticipate deployment of both PV and wind in mountaneous areas (e.g.,[45]). Due to the need for cooling water supply, easy transport of heavy components, links to transmission networks, and the potential difficulties in seismic designs for steep terrains, nuclear power plants are less likely to be constructed in mountain terrain. A potential linkage to mountain biodiversity can, however, come from mining industries located in mountain areas. We capture these findings by assigning for indicator 15.4.2 a '-1' to all mining industries (for all technologies in our set), a '-1' to solar and PV construction and power generation, and a '0' to nuclear construction and power generation. For cookstoves, we assign a '-1' to industries related to agriculture, since deployment of cookstoves in mountain regions may involve direct sourcing of fuelwood from these areas. We do not assume any potential for indicator 15.4.1 because any interaction of energy technology manufacturing, deployment, or operation with mountain biodiversity does not directly affect coverage by protected areas.

S-13.3. Other SDG15 indicators

Our review did not identify reasons to expect a relationship between energy technology investments and indicators related to conservation (indicator 15.5.1, 'Red List Index'), the sharing of genetic resources (15.6.1, 'Number of countries that have adopted legislative, administrative and policy frameworks to ensure fair and equitable sharing of benefits'), wildlife trafficking (15.7.1, 'Proportion of traded wildlife that was poached or illicitly trafficked'), the control of invasive species (15.8.1, 'Proportion of countries adopting relevant national legislation and adequately resourcing the prevention or control of invasive alien species'), integrated ecosystem and biodiversity planning (15.9.1, 'Progress towards national targets established in accordance with Aichi Biodiversity Target 2 of the Strategic Plan for Biodiversity 2011-2020'), and development assistance for biodiversity and forest management (15.A.1, 15.B.1). Desirable changes in all these indicators may correlate with energy technology investments as part of a broader (and successful) push for sustainability, but we do not identify any direct connections. (For example, the manufacturing and deployment of energy technologies typically does not require or induce better sharing of genetic resources or better control of invasive species.)

S-14. SDG16: Promote just, peaceful and inclusive societies

We did not find evidence indicating potential linkages between energy technology investments and indicators of SDG16. For some indicators, this is due to lack of data availability. Data on intentional homicides (16.1.1), conflict-related deaths (16.1.2), exposure to non-lethal forms of physical violence (16.1.3) or sexual violence (16.2.3), and dispute resolution frequency (16.3.3) are collected at the country-level and not available by industry. Even if data was available by industry, it is likely any differences would be circumstantial (conflicts and violence that occurred in specific factories for specific reasons) rather than due to a causal linkage between manufacturing activities and the metrics captured by SDG16 indicators.

Other indicators related to public safety (indicator 16.1.4), violence by caregivers against children (16.2.1), human trafficking (16.2.2), crime reporting frequencies (16.3.1), unsentenced prison detainee proportions (16.3.2), seized arms (16.4.2), the proportion of population who believe decision-making is inclusive (16.7.2), the proportion of births that are registered (16.9.1), and the number of verified journalist killings (16.10.1) are entirely unrelated to specific energy technology industry and service characteristics. We therefore do not assume any potential for technology investments to influence these indicators.

Lastly, there are also indicators for which the UN's indicator definition precludes a direct connection to technology-related industries or services, even though a connection to the broader indicator theme might exist. For example, bribery indicators (15.6.1, 15.6.2) are defined for contacts with public officials, not for bribery in business transactions. Similarly, the indicators for target 16.6 ('Develop effective, accountable and transparent institutions') do not cover institutional capacities of industries at all. For example, the existence of national labs and industry organizations that monitor energy technology performance and regularly share standardizaed information with the public would not be captured by the indicators of SDG16 (or by any indicators, including those under SDG9). We discuss this issue in section 3.3 in the main text.

S-15. SDG17: Revitalize the global partnership for sustainable development

S-15.1. Indicator 17.7.1

The only indicator for which we find potential linkages to energy-related industries and services is 17.17.1 ('Amount of United States dollars committed to (a) public-private partnerships and (b) civil society partnerships'). Searching for energy- and electricity infrastructure projects involving public private partnerships in The World Bank's Private Participation in Infrastructure Database yields multiple hits for wind, solar PV, and nuclear fission,[46] apart from other energy technologies not covered in our set. Clean cookstoves are not included in this database as it is focused on infrastructure projects, but public-private partnerships to promote clean cookstove adoption exist (most notably the Global Alliance for Clean Cookstoves, a public-private partnership hosted by the United Nations Foundation) and therefore appear possible in the future.

To isolate specific technology industries where additional investment is likely to influence indicator 17.17.1 positively, we take industries' and sectors' coverage by the World Bank's public private partnership legal resource center[47] as an indication of additional investment in these industries having the potential to increase funds committed to public private partnerships. These industries include energy and power, telecommunications, information and communications technology, transportation infrastructure, and water and sanitation.

Given the above findings, we note a '1' in all cells representing connections between industries likely to increase funds committed to infrastructure project delivery through public private partnerships, and '0' to all other industries. These other industries include most mining and manufacturing industries required to build energy technologies. Our rationale is that these industries are likely outside the boundary of a public private partnership designed to deliver an infrastructure project, in the sense that the partnership will involve component purchase and construction but focus to a lesser degree on scaling underlying mining and sub-component manufacturing industries.

S-15.2. All other SDG17 indicators

For most other SDG17 indicators it is difficult to draw a connection to energy technology industries and services. In some cases, the indicator definitions are too broad to relate them to specific industries. For example, we did not find evidence for technology investments directly influencing

the share of government revenue in total GDP (17.1.1), domestic taxes (17.1.2), volume of remittances (17.3.2), or debt service (17.4.1). Changes in indicator 17.1.1 related to technology industries will depend on the tax rate applied to these industries relative to other industries, and the growth and resulting contribution to GDP of technology-related industries relative to other industries contributing to the GDP. For development assistance and direct foreign investment (17.2.1, 17.3.1), as well as for the number of countries adopting investment promotion schemes (17.5.1), and the dollar value of financial and technical assistance committed to developing countries (17.9.1) there is a potential for positive changes in these indicators to induce investments in technology industries and services, instead of the other way around.

For indicators related to internet access, we assign a '1' to the connection between power generation industries (solar PV, wind, and nuclear) and the respective indicators (17.6.1, the number of fixed Internet broadband subscriptions; 17.8.1., the proportion of individuals using the Internet) due to the relationships between electricity and internet access. We note that this is an indirect connection, however, since electricity is a necessary but not sufficient condition for internet access.

For indicator 17.11.1 ('developing countriesâ and least developed countriesâ share of global exports'), we assign a '1' to all technology industries and services. This indicator provides calculations of developing countries exports of goods and services toward the rest of the world, and all items in our technology networks are either industries or services.

**SDG1**	**End poverty in all its forms everywhere**
**Indicator**	**Indicator name**
1.1.1	Prop. of population below the international poverty line, by sex, age, employment status and geographical location (urban/rural)
1.2.1	Prop. of population living below the national poverty line, by sex and age
1.2.2	Prop. of men, women and children of all ages living in poverty in all its dimensions according to national definitions
1.3.1	Prop. of population covered by social protection floors/systems, by sex, distinguishing children, unemployed persons, older persons, persons with disabilities, pregnant women, newborns, work-injury victims and the poor and the vulnerable
1.4.1	Prop. of population living in households with access to basic services
1.4.2	Prop. of total adult population with secure tenure rights to land, with legally recognized documentation, and who perceive their rights to land as secure, by sex and type of tenure
1.5.1	Number of deaths, missing persons and directly affected persons attributed to disasters per 100,000 population
1.5.2	Direct economic loss attributed to disasters in relation to global gross domestic product (GDP)
1.5.3	Number of countries that adopt and implement national disaster risk reduction strategies in line with the Sendai Framework for Disaster Risk Reduction 2015-2030
1.5.4	Prop. of local governments that adopt and implement local disaster risk reduction strategies in line with national disaster risk reduction strategies
1.a.1	Prop. of domestically generated resources allocated by the government directly to poverty reduction programmes
1.a.2	Prop. of total government spending on essential services (education, health and social protection)
1.a.3	Sum of total grants and non-debt-creating inflows directly allocated to poverty reduction programmes as a prop. of GDP
1.b.1	Prop. of government recurrent and capital spending to sectors that disproportionately benefit women, the poor and vulnerable groups

**SDG2**	**End hunger, achieve food security and improved nutrition and promote sustainable agriculture**
2.1.1	Prevalence of undernourishment
2.1.2	Prevalence of moderate or severe food insecurity in the population, based on the Food Insecurity Experience Scale (FIES)
2.2.1	Prevalence of stunting (height for age <-2 standard deviation from the median of the World Health Organization (WHO) Child Growth Standards) among children under 5 years of age
2.2.2	Prevalence of malnutrition (weight for height >+2 or <-2 standard deviation from the median of the WHO Child Growth Standards) among children under 5 years of age, by type
2.3.1	Volume of production per labour unit by classes of farming/pastoral/forestry enterprise size
2.3.2	Average income of small-scale food producers, by sex and indigenous status
2.4.1	Prop. of agricultural area under productive and sustainable agriculture
2.5.1	Number of plant and animal genetic resources for food and agriculture secured in either medium- or long- term conservation facilities
2.5.2	Prop. of local breeds classified as being at risk, not at risk or at unknown level of risk of extinction
2.a.1	The agriculture orientation index for government expenditures
2.a.2	Total official flows (official development assistance, other official flows) to the agriculture sector
2.b.1	Agricultural export subsidies
2.c.1	Indicator of food price anomalies

**SDG3**	**Ensure healthy lives and promote well-being for all at all ages**
3.1.1	Maternal mortality ratio
3.1.2	Prop. of births attended by skilled health personnel
3.2.1	Under-5 mortality rate
3.2.2	Neonatal mortality rate
3.3.1	Number of new HIV infections per 1,000 uninfected, by sex, age and key populations

Table S-1: **SDG indicators as defined by the UN,[48] Related to STAR Methods.** Proportion is abbreviated as 'Prop'.

SDG 3 cont.	
**Indicator**	**Indicator name**
3.3.2	Tuberculosis incidence per 100,000 population
3.3.3	Malaria incidence per 1,000 population
3.3.4	Hepatitis B incidence per 100,000 population
3.3.5	Number of people requiring interventions against neglected tropical diseases
3.4.1	Mortality rate attributed to cardiovascular disease, cancer, diabetes or chronic respiratory disease
3.4.2	Suicide mortality rate
3.5.1	Coverage of treatment interventions (pharmacological, psychosocial and rehabilitation and aftercare services) for substance use disorders
3.5.2	Harmful use of alcohol, defined according to the national context as alcohol per capita consumption (aged 15 years and older) within a calendar year in litres of pure alcohol
3.6.1	Death rate due to road traffic injuries
3.7.1	Prop. of women of reproductive age (aged 15-49 years) who have their need for family planning satisfied with modern methods
3.7.2	Adolescent birth rate (aged 10-14 years; aged 15-19 years) per 1,000 women in that age group
3.8.1	Coverage of essential health services (defined as the average coverage of essential services based on tracer interventions that include reproductive, maternal, newborn and child health, infectious diseases, non-communicable diseases and service capacity and access, among the general and the most disadvantaged population)
3.8.2	Prop. of population with large household expenditures on health as a share of total household expenditure or income
3.9.1	Mortality rate attributed to household and ambient air pollution
3.9.2	Mortality rate attributed to unsafe water, unsafe sanitation and lack of hygiene (exposure to unsafe Water, Sanitation and Hygiene for All (WASH) services)
3.9.3	Mortality rate attributed to unintentional poisoning
3.a.1	Age-standardized prevalence of current tobacco use among persons aged 15 years and older
3.b.1	Prop. of the target population covered by all vaccines included in their national programme
3.b.2	Total net official development assistance to medical research and basic health sectors
3.b.3	Prop. of health facilities that have a core set of relevant essential medicines available and affordable on a sustainable basis
3.c.1	Health worker density and distribution
3.d.1	International Health Regulations (IHR) capacity and health emergency preparedness

**SDG4**	**Ensure inclusive and equitable quality education and promote life-long learning opportunities for all**
4.1.1	Prop. of children and young people (a) in grades 2/3; (b) at the end of primary; and (c) at the end of lower secondary achieving at least a minimum proficiency level in (i) reading and (ii) mathematics, by sex
4.2.1	Prop. of children under 5 years of age who are developmentally on track in health, learning and psychosocial well-being, by sex
4.2.2	Participation rate in organized learning (one year before the official primary entry age), by sex
4.3.1	Participation rate of youth and adults in formal and non-formal education and training in the previous 12 months, by sex
4.4.1	Prop. of youth and adults with information and communications technology (ICT) skills, by type of skill
4.5.1	Parity indices (female/male, rural/urban, bottom/top wealth quintile and others such as disability status, indigenous peoples and conflict-affected, as data become available) for all education indicators on this list that can be disaggregated
4.6.1	Prop. of population in a given age group achieving at least a fixed level of proficiency in functional (a) literacy and (b) numeracy skills, by sex
4.7.1	Extent to which (i) global citizenship education and (ii) education for sustainable development, including gender equality and human rights, are mainstreamed at all levels in (a) national education policies; (b) curricula; (c) teacher education; and (d) student assessment

Table S-2: **SDG indicators as defined by the UN,[48] Related to STAR Methods.** Proportion is abbreviated as 'Prop'.

**SDG 4 cont.**	
**Indicator**	**Indicator name**
4.a.1	Prop. of schools with access to (a) electricity; (b) the Internet for pedagogical purposes; (c) computers for pedagogical purposes; (d) adapted infrastructure and materials for students with disabilities; (e) basic drinking water; (f) single-sex basic sanitation facilities; and (g) basic handwashing facilities (as per the WASH indicator definitions)
4.b.1	Volume of official development assistance flows for scholarships by sector and type of study
4.c.1	Prop. of teachers in (a) pre-primary; (b) primary; (c) lower secondary; and (d) upper secondary education who have received at least the minimum organized teacher training (e.g. pedagogical training) pre-service or in-service required for teaching at the relevant level in a given country

**SDG5**	**Achieve gender equality and empower all women and girls**
5.1.1	Whether or not legal frameworks are in place to promote, enforce and monitor equality and non-discrimination on the basis of sex
5.2.1	Prop. of ever-partnered women and girls aged 15 years and older subjected to physical, sexual or psychological violence by a current or former intimate partner in the previous 12 months, by form of violence and by age
5.2.2	Prop. of women and girls aged 15 years and older subjected to sexual violence by persons other than an intimate partner in the previous 12 months, by age and place of occurrence
5.3.1	Prop. of women aged 20-24 years who were married or in a union before age 15 and before age 18
5.3.2	Prop. of girls and women aged 15-49 years who have undergone female genital mutilation/cutting, by age
5.4.1	Prop. of time spent on unpaid domestic and care work, by sex, age and location
5.5.1	Prop. of seats held by women in (a) national parliaments and (b) local governments
5.5.2	Prop. of women in managerial positions
5.6.1	Prop. of women aged 15-49 years who make their own informed decisions regarding sexual relations, contraceptive use and reproductive health care
5.6.2	Number of countries with laws and regulations that guarantee full and equal access to women and men aged 15 years and older to sexual and reproductive health care, information and education
5.a.1	(a) Prop. of agricultural population with ownership or secure rights over agricultural land, by sex; (b) share of women among owners or rights-bearers of agricultural land, by type of tenure
5.a.2	Prop. of countries where the legal framework (including customary law) guarantees women's equal rights to land ownership and/or control
5.b.1	Prop. of individuals who own a mobile telephone, by sex
5.c.1	Prop. of countries with systems to track and make public allocations for gender equality and women's empowerment

**SDG6**	**Ensure availability and sustainable management of water and sanitation for all**
6.1.1	Prop. of population using safely managed drinking water services
6.2.1	Prop. of population using (a) safely managed sanitation services and (b) a hand-washing facility with soap and water
6.3.1	Prop. of wastewater safely treated
6.3.2	Prop. of bodies of water with good ambient water quality
6.4.1	Change in water-use efficiency over time
6.4.2	Level of water stress: freshwater withdrawal as a prop. of available freshwater resources
6.5.1	Degree of integrated water resources management implementation (0â100)
6.5.2	Prop. of transboundary basin area with an operational arrangement for water cooperation
6.6.1	Change in the extent of water-related ecosystems over time
6.a.1	Amount of water- and sanitation-related official development assistance that is part of a government-coordinated spending plan
6.b.1	Prop. of local administrative units with established and operational policies and procedures for participation of local communities in water and sanitation management

Table S-3: **SDG indicators as defined by the UN,[48] Related to STAR Methods.** Proportion is abbreviated as 'Prop'.

SDG7	Ensure access to affordable, reliable, sustainable and modern energy for all
**Indicator**	**Indicator name**
7.1.1	Prop. of population with access to electricity
7.1.2	Prop. of population with primary reliance on clean fuels and technology
7.2.1	Renewable energy share in the total final energy consumption
7.3.1	Energy intensity measured in terms of primary energy and GDP
7.a.1	International financial flows to developing countries in support of clean energy research and development and renewable energy production, including in hybrid systems
7.b.1	Investments in energy efficiency as a prop. of GDP and the amount of foreign direct investment in financial transfer for infrastructure/technology to sustainable development services

SDG8	Promote sustained, inclusive and sustainable economic growth, full and productive employment and decent work for all
**Indicator**	**Indicator name**
8.1.1	Annual growth rate of real GDP per capita
8.2.1	Annual growth rate of real GDP per employed person
8.3.1	Prop. of informal employment in non-agriculture employment, by sex
8.4.1	Material footprint, material footprint per capita, and material footprint per GDP
8.4.2	Domestic material consumption, domestic material consumption per capita, and domestic material consumption per GDP
8.5.1	Average hourly earnings of female and male employees, by occupation, age and persons with disabilities
8.5.2	Unemployment rate, by sex, age and persons with disabilities
8.6.1	Prop. of youth (aged 15-24 years) not in education, employment or training
8.7.1	Prop. and number of children aged 5-17 years engaged in child labour, by sex and age
8.8.1	Frequency rates of fatal and non-fatal occupational injuries, by sex and migrant status
8.8.2	Level of national compliance with labour rights (freedom of association and collective bargaining) based on International Labour Organization textual sources and national legislation, by sex and migrant status
8.9.1	Tourism direct GDP as a prop. of total GDP and in growth rate
8.9.2	Prop. of jobs in sustainable tourism industries out of total tourism jobs
8.10.1	(a) Number of commercial bank branches per 100,000 adults and (b) number of automated teller machines (ATMs) per 100,000 adults
8.10.2	Prop. of adults (15 years and older) with an account at a bank or other financial institution or with a mobile-money-service provider
8.a.1	Aid for Trade commitments and disbursements
8.b.1	Existence of a developed and operationalized national strategy for youth employment, as a distinct strategy or as part of a national employment strategy

SDG9	Build resilient infrastructure, promote inclusive and sustainable industrialization and foster innovation
**Indicator**	**Indicator name**
9.1.1	Prop. of the rural population who live within 2 km of an all-season road
9.1.2	Passenger and freight volumes, by mode of transport
9.2.1	Manufacturing value added as a prop. of GDP and per capita
9.2.2	Manufacturing employment as a prop. of total employment
9.3.1	Prop. of small-scale industries in total industry value added
9.3.2	Prop. of small-scale industries with a loan or line of credit
9.4.1	CO_2 emission per unit of value added
9.5.1	Research and development expenditure as a prop. of GDP
9.5.2	Researchers (in full-time equivalent) per million inhabitants
9.a.1	Total official international support (official development assistance plus other official flows) to infrastructure
9.b.1	Prop. of medium and high-tech industry value added in total value added
9.c.1	Prop. of population covered by a mobile network, by technology

Table S-4: **SDG indicators as defined by the UN,[48] Related to STAR Methods.** Proportion is abbreviated as ‘Prop.’.

SDG10	Reduce inequality within and among countries
**Indicator**	**Indicator name**
10.1.1	Growth rates of household expenditure or income per capita among the bottom 40 per cent of the population and the total population
10.2.1	Prop. of people living below 50 per cent of median income, by sex, age and persons with disabilities
10.3.1	Prop. of population reporting having personally felt discriminated against or harassed in the previous 12 months on the basis of a ground of discrimination prohibited under international human rights law
10.4.1	Labour share of GDP, comprising wages and social protection transfers
10.5.1	Financial Soundness Indicators
10.6.1	Prop. of members and voting rights of developing countries in international organizations
10.7.1	Recruitment cost borne by employee as a prop. of yearly income earned in country of destination
10.7.2	Number of countries that have implemented well- managed migration policies
10.a.1	Prop. of tariff lines applied to imports from least developed countries and developing countries with zero-tariff
10.b.1	Total resource flows for development, by recipient and donor countries and type of flow (e.g. official development assistance, foreign direct investment and other flows)
10.c.1	Remittance costs as a prop. of the amount remitted

SDG11	Make cities and human settlements inclusive, safe, resilient and sustainable
11.1.1	Prop. of urban population living in slums, informal settlements or inadequate housing
11.2.1	Prop. of population that has convenient access to public transport, by sex, age and persons with disabilities
11.3.1	Ratio of land consumption rate to population growth rate
11.3.2	Prop. of cities with a direct participation structure of civil society in urban planning and management that operate regularly and democratically
11.4.1	Total expenditure (public and private) per capita spent on the preservation, protection and conservation of all cultural and natural heritage, by type of heritage (cultural, natural, mixed and World Heritage Centre designation), level of government (national, regional and local/municipal), type of expenditure (operating expenditure/investment) and type of private funding (donations in kind, private non-profit sector and sponsorship)
11.5.1	Number of deaths, missing persons and directly affected persons attributed to disasters per 100,000 population
11.5.2	Direct economic loss in relation to global GDP, damage to critical infrastructure and number of disruptions to basic services, attributed to disasters
11.6.1	Prop. of urban solid waste regularly collected and with adequate final discharge out of total urban solid waste generated, by cities
11.6.2	Annual mean levels of fine particulate matter (e.g. PM2.5, PM10) in cities (population weighted)
11.7.1	Average share of the built-up area of cities that is open space for public use for all, by sex, age and persons with disabilities
11.7.2	Prop. of persons victim of physical or sexual harassment, by sex, age, disability status and place of occurrence, in the previous 12 months
11.a.1	Prop. of population living in cities that implement urban and regional development plans integrating population projections and resource needs, by size of city
11.b.1	Number of countries that adopt and implement national disaster risk reduction strategies in line with the Sendai Framework for Disaster Risk Reduction 2015-2030
11.b.2	Prop. of local governments that adopt and implement local disaster risk reduction strategies in line with national disaster risk reduction strategies
11.c.1	Prop. of financial support to the least developed countries that is allocated to the construction and retrofitting of sustainable, resilient and resource- efficient buildings utilizing local materials

Table S-5: **SDG indicators as defined by the UN,[48] Related to STAR Methods.** Proportion is abbreviated as 'Prop.'.

**SDG12**	**Ensure sustainable consumption and production patterns**
**Indicator**	**Indicator name**
12.1.1	Number of countries with sustainable consumption and production (SCP) national action plans or SCP mainstreamed as a priority or a target into national policies
12.2.1	Material footprint, material footprint per capita, and material footprint per GDP
12.2.2	Domestic material consumption, domestic material consumption per capita, and domestic material consumption per GDP
12.3.1	Global food loss index
12.4.1	Number of parties to international multilateral environmental agreements on hazardous waste, and other chemicals that meet their commitments and obligations in transmitting information as required by each relevant agreement
12.4.2	Hazardous waste generated per capita and proportion of hazardous waste treated, by type of treatment
12.5.1	National recycling rate, tons of material recycled
12.6.1	Number of companies publishing sustainability reports
12.7.1	Number of countries implementing sustainable public procurement policies and action plans
12.8.1	Extent to which (i) global citizenship education and (ii) education for sustainable development (including climate change education) are mainstreamed in (a) national education policies; (b) curricula; (c) teacher education; and (d) student assessment
12.a.1	Amount of support to developing countries on research and development for sustainable consumption and production and environmentally sound technologies
12.b.1	Number of sustainable tourism strategies or policies and implemented action plans with agreed monitoring and evaluation tools
12.c.1	Amount of fossil-fuel subsidies per unit of GDP (production and consumption) and as a prop. of total national expenditure on fossil fuels

**SDG13**	**Take urgent action to combat climate change and its impacts**
**Indicator**	**Indicator name**
13.1.1	Number of deaths, missing persons and directly affected persons attributed to disasters per 100,000population
13.1.2	Number of countries that adopt and implement national disaster risk reduction strategies in line with the Sendai Framework for Disaster Risk Reduction 2015â2030
13.1.3	Prop. of local governments that adopt and implement local disaster risk reduction strategies in line with national disaster risk reduction strategies
13.2.1	Number of countries that have communicated the establishment or operationalization of an integrated policy/strategy/plan which increases their ability to adapt to the adverse impacts of climate change, and foster climate resilience and low greenhouse gas emissions development in a manner that does not threaten food production (including a national adaptation plan, nationally determined contribution, national communication, biennial update report or other)
13.3.1	Number of countries that have integrated mitigation, adaptation, impact reduction and early warning into primary, secondary and tertiary curricula
13.3.2	Number of countries that have communicated the strengthening of institutional, systemic and individual capacity-building to implement adaptation, mitigation and technology transfer, and development actions
13.a.1	Mobilized amount of United States dollars per year between 2020 and 2025 accountable towards the $100 billion commitment
13.b.1	Number of LDC and SIDS that are receiving specialized support, and amount of support, including finance, technology and capacity-building, for mechanisms for raising capacities for effective climate change-related planning and management, including focusing on women, youth and local and marginalized communities

Table S-6: **SDG indicators as defined by the UN,[48] Related to STAR Methods.** SIDS= small island developing states; LDC= least developed countries. Proportion is abbreviated as ‘Prop.’.

SDG14	Conserve and sustainably use the oceans, seas and marine resources for sustainable development
**Indicator**	**Indicator name**
14.1.1	Index of coastal eutrophication and floating plastic debris density
14.2.1	Prop. of national exclusive economic zones managed using ecosystem-based approaches
14.3.1	Average marine acidity (pH) measured at agreed suite of representative sampling stations
14.4.1	Prop. of fish stocks within biologically sustainable levels
14.5.1	Coverage of protected areas in relation to marine areas
14.6.1	Progress by countries in the implementation of int. instruments to combat illegal, unreported/unregulated fishing
14.7.1	Sustainable fisheries as a prop. of GDP in SIDS, LDC, and all countries
14.a.1	Prop. of total research budget allocated to research in the field of marine technology
14.b.1	Progress by countries in the degree of application of a legal/regulatory/ policy/institutional framework which recognizes and protects access rights for small-scale fisheries
14.c.1	Number of countries making progress in ratifying, accepting and implementing through legal, policy and institutional frameworks, ocean-related instruments that implement international law, as reflected in the UNCLOS, for the conservation and sustainable use of the oceans and their resources

SDG15	Protect, restore and promote sustainable use of terrestrial ecosystems, sustainably manage forests, combat desertification, halt and reverse land degradation and halt biodiversity loss
15.1.1	Forest area as a prop. of total land area
15.1.2	Prop. of important sites for terrestrial and freshwater biodiversity covered by protected areas
15.2.1	Progress towards sustainable forest management
15.3.1	Prop. of land that is degraded over total land area
15.4.1	Coverage by protected areas of important sites for mountain biodiversity
15.4.2	Mountain Green Cover Index
15.5.1	Red List Index
15.6.1	Nr. of countries with leg., admin. and policy frameworks to ensure fair and equitable sharing of benefits
15.7.1	Prop. of traded wildlife that was poached or illicitly trafficked
15.8.1	Prop. of countries adopting relevant national legislation and adequately resourcing the prevention or control of invasive alien species
15.9.1	Progress towards national targets under Aichi Biodiversity Target 2
15.a/b.1	Official dev. assistance and public expenditure on conservation and sustainable use of biodiversity and ecosystems
15.c.1	Prop. of traded wildlife that was poached or illicitly trafficked

SDG16	Promote peaceful and inclusive societies for sustainable development, provide access to justice for all and build effective, accountable and inclusive institutions at all levels
16.1.1	Number of victims of intentional homicide per 100,000 population, by sex and age
16.1.2	Conflict-related deaths per 100,000 population, by sex, age and cause
16.1.3	Prop. of population subjected to physical, psychological and sexual violence in the previous 12 m.
16.1.4	Prop. of population that feel safe walking alone around the area they live
16.2.1	Prop. of children (1-17 y.) who experienced any physical punishment and/or psychological aggression by caregivers in the past m.
16.2.2	Number of victims of human trafficking per 100,000 population, by sex, age and form of exploitation
16.2.3	Prop. of young women and men aged 18-29 years who experienced sexual violence by age 18
16.3.1	Prop. of victims of violence in the previous 12 months who reported their victimization to competent authorities or other officially recognized conflict resolution mechanisms
16.3.2	Unsentenced detainees as a prop. of overall prison population
16.4.1	Total value of inward and outward illicit financial flows (in current United States dollars)
16.4.2	Prop. of seized, found or surrendered arms whose illicit origin or context has been traced or established by a competent authority in line with international instruments
16.5.1	Prop. of persons who had at least one contact with a public official and who paid a bribe to a public official, or were asked for a bribe by those public officials, during the previous 12 m.
16.5.2	Prop. of businesses that had at least one contact with a public official and that paid a bribe to a public official, or were asked for a bribe by those public officials during the previous 12 m.
16.6.1	Primary government expenditures as a prop. of original approved budget, by sector or budget codes
16.6.2	Prop. of population satisfied with their last experience of public services
16.7.1	Prop. of positions (by sex, age, persons with disabilities, population groups) in public institutions (national/local legislatures, public service, and judiciary) compared to national distributions

Table S-7: **SDG indicators as defined by the UN,[48] Related to STAR Methods.** Proportion is abbreviated as 'Prop.' and UNCLOS refers to the United Nations Convention on the Law of the Sea.

SDG16 cont.	
**Indicator**	**Indicator name**
16.7.2	Prop. of population who believe decision- making is inclusive and responsive, by sex, age, disability and population group
16.8.1	Prop. of members and voting rights of developing countries in international organizations
16.9.1	Proportion of children under 5 years whose births have been registered with a civil authority, by age
16.10.1	Number of verified cases of killing, kidnapping, enforced disappearance, arbitrary detention and torture of journalists, associated media personnel, trade unionists and human rights advocates in the previous 12 months
16.10.2	Number of countries that adopt and implement constitutional, statutory and/or policy guarantees for public access to information
16.a.1	Existence of independent national human rights institutions in compliance with the Paris Principles
16.b.1	Prop. of population reporting having personally felt discriminated against or harassed in the previous 12 months on the basis of a ground of discrimination prohibited under international human rights law

**SDG17**	**Strengthen the means of implementation and revitalize the Global Partnership for Sustainable Development**
17.1.1	Total government revenue as a prop. of GDP, by source
17.1.2	Prop. of domestic budget funded by domestic taxes
17.2.1	Net official development assistance, total and to LDC, as a prop. of the Organization for Economic Cooperation and Development (OECD) Development Assistance Committee donors' gross national income (GNI)
17.3.1	Foreign direct investment (FDI), official development assistance and South-South cooperation as a prop. of total domestic budget
17.3.2	Volume of remittances (in United States dollars) as a prop. of total GDP
17.4.1	Debt service as a prop. of exports of goods and services
17.5.1	Number of countries that adopt and implement investment promotion regimes for LDC
17.6.1	Number of science and/or technology cooperation agreements and programmes between countries, by type of cooperation
17.6.2	Fixed Internet broadband subscriptions per 100 inhabitants, by speed
17.7.1	Total amount of approved funding for developing countries to promote the development, transfer, dissemination and diffusion of environmentally sound technologies
17.8.1	Prop. of individuals using the Internet
17.9.1	Dollar value of financial and technical assistance (including through North-South, South-South and triangular cooperation) committed to developing countries
17.10.1	Worldwide weighted tariff-average
17.11.1	Developing countries' and LDC's share of global exports
17.12.1	Average tariffs faced by developing countries, LDC and SIDS
17.13.1	Macroeconomic Dashboard
17.14.1	Number of countries with mechanisms in place to enhance policy coherence of sustainable development
17.15.1	Extent of use of country-owned results frameworks and planning tools by providers of development cooperation
17.16.1	Number of countries reporting progress in multi- stakeholder development effectiveness monitoring frameworks that support the achievement of the Sustainable Development Goals
17.17.1	Amount of United States dollars committed to (a) public-private partnerships and (b) civil society partnerships
17.18.1	Prop. of sustainable development indicators produced at the national level with full disaggregation when relevant to the target, in accordance with the FPOS
17.18.2	Number of countries that have national statistical legislation that complies with the FPOS
17.18.3	Number of countries with a national statistical plan that is fully funded and under implementation, by source of funding
17.19.1	Dollar value of all resources made available to strengthen statistical capacity in developing countries
17.19.2	Prop. of countries that have conducted at least one population/ housing census in the last 10 y. and have achieved 100 per cent birth registration and 80 per cent death registration

Table S-8: **SDG indicators as defined by the UN,[48] Related to STAR Methods.** Proportion is abbreaviated as 'Prop.', Fundamental Principles of Official Statistics as FPOS, small island developing states as SIDS, and least developed countries as LDC.

PV SYSTEMS

**NAICS Code**	**Industry**	**Component/Installation step**
Power generation		
221113	Solar electric power generation	All
Mining		
212210	Iron ore mining	Inverter (steel)
212230	Copper, nickel, lead, and zinc mining	Module interconnections, cables, inverter
212299	All other metal ore mining (aluminum, etc.)	Module frame, inverter, mounting system
212322	Industrial sand mining	PV panel
Construction		
237130	Power and communication line and related structures construction	All
Specialty trade contractors		
238210	Electrical contractors and other wiring installation contractors	Electrical installation
238910	Site preparation contractors	Site preparation
Manufacturing		
311110	Iron and steel mills and ferroalloy manufacturing	Inverter (steel)
327211	Flat glass manufacturing	Panel front/back glass
331313	Alumina refining	Panel frame, mounting rails, inverter
331314	Alumina secondary smelting	See line above
334412	Bare circuit board manufacturing	Inverter
334413	Semiconductor and related device manufacturing (includes solar cell manufacturing)	Cells, inverter
334417	Electrical connector manufacturing	Cable connections
334418	Printed circuit assembly manufacturing	Inverter
334515	Instrument manufacturing for measuring and testing electricity and electrical signals	Inverter, system monitor
325211	Plastics material and resin manufacturing	Encapsulation (ethylvinylacetate), backsheet (polyethylene terephthalate, polyvinylfluoride film)
335931	Current-carrying wiring device manufacturing	Cables
Merchant wholesalers		
423690	Other electronic parts and equipment wholesalers	Component purchase
Transportation		
483111	Deep sea freight transportation	Component shipping
484122	General freight trucking, long-distance	Component shipping
Warehousing		
493110	Warehousing and storage	Component storage
Finance and insurance		
522110	Commercial banking	Financing
Professional services		
541310	Architectural services	System design, building integration
541330	Engineering services	System design
541350	Building inspection services	Inspection, permitting
Administrative support and	*waste management remediation services*	
562111	Solid waste collection	Collection of all components
562112	Hazardous waste collection	Panels containing heavy metals (e.g., silver, copper, lead, arsenic) above threshold toxicity concentrations
562212	Solid waste landfill	PV waste is landfilled in some countries[49]
562213	Solid Waste Combustors and Incinerators	Panels[50]

Table S-9: **Industries and services required for manufacturing and deploying the components of a photovoltaic system, listed in ascending order based on 3-digit NAICS codes, Related to STAR Methods.** Material inputs are based on the bill of materials for multi-crystalline silicon panels and inverters in[51] and matched to industries in the NAICS code.

WIND (PART 1)		
**NAICS Code**	**Industry**	**Component/Installation step**
Power generation		
221115	Wind electric power generation	
Mining		
212210	Iron ore mining	Tower, foundation
212230	Copper, nickel, lead, and zinc mining	Tower, foundation, cables, switchgears
212299	All other metal ore mining (bauxite, etc.)	Turbine, cables (bauxite), turbine generator (e.g., neodymium, dysprosium, terbium)
212312	Crushed and broken limestone mining and quarrying	Access roads
212321	Construction sand and gravel mining	Access roads
Construction		
237130	Power and communication line and related structures construction	Full system
Specialty trade contractors		
238110	Poured concrete foundation and structure contractors	Tower foundation
238210	Electrical contractors and other wiring installation contractors	Electrical installation
238910	Site preparation contractors	Site preparation
Manufacturing		
331313	Alumina refining	Turbine, cables
331314	Alumina secondary smelting	Turbine, cables
334412	Bare circuit board manufacturing	Controller
334413	Semiconductor and related device manufacturing	Controller
334417	Electrical connector manufacturing	Cable connectors
334418	Printed circuit assembly manufacturing	Controller
334515	Instrument manufacturing for measuring and testing electricity and electrical signals	Controller, anemometer
335931	Current-carrying wiring device manufacturing	Cables
331410	Non-ferrous (except aluminum) metal melting and refining	
331492	Secondary smelting, refining, and alloying of nonferrous metal	Tower, gearbox
332312	Fabricated structural metal manufacturing	Tower
332313	Plate work manufacturing	Tower
332811	Metal heat treating	Tower
333611	Turbine and turbine generator set units manufacturing	Turbine
333612	Speed changer, industrial high-speed drive, and gear manufacturing	Gearbox
335311	Power, distribution, and specialty transformer manufacturing	Step-up transformer inside nacelle
325220	Artificial and synthetic fibers and filaments manufacturing	Turbine blades
327320	Ready mix concrete manufacturing	Tower foundation
Merchant wholesalers		
423320	Brick, stone, and related construction material merchant wholesalers	Component purchase
423690	Other electronic parts and equipment wholesalers	Component purchase

Table S-10: **Industries and services required for manufacturing and deploying the components of wind turbines, listed in ascending order based on 3-digit NAICS codes, Related to STAR Methods.** Material inputs are based on the bill of materials for a 4.2 MW onshore wind turbine as given in[52] and matched to industries in the NAICS code.

WIND (PART 2)		
**NAICS Code**	**Industry**	**Component/Installation step**
Transportation		
483111	Deep sea freight transportation	Component shipping
484122	General freight trucking, long-distance	Component shipping
Warehousing		
493110	Warehousing and storage	Component storage
Finance and insurance		
522110	Commercial banking	Financing
Professional services		
541330	Engineering services	System design, construction
541360	Geophysical surveying	Site selection
Administrative support and waste management remediation services		
562111	Solid waste collection	Collection of all components
562212	Solid waste landfill	Landfill and incineration are the main end of life strategies for wind turbines currently.[53]
562213	Solid Waste Combustors and Incinerators	See above.

Table S-11: **Industries and services required for manufacturing and deploying the components of wind turbines, listed in ascending order based on 3-digit NAICS codes, Related to STAR Methods.** Material inputs are based on the bill of materials for a 4.2 MW onshore wind turbine as given in[52] and matched to industries in the NAICS code.

NUCLEAR FISSION PLANTS	(PART 1)	
**NAICS Code**	**Industry**	Component/installation step
Power generation		
221113	Nuclear electric power generation	All
Mining		
212230	Copper, nickel, lead, and zinc mining	Electrical components, pressure vessel
212291	Iron ore mining	Pressure vessel, all structures using reinforced concrete
212291	Uranium-radium-vanadium ore mining	Fuel assemblies
212299	All other metal ore mining (bauxite, etc.)	Pressure vessel, fuel cladding, other plant components
212322	Industrial sand mining	Access roads
212399	All other nonmetallic mineral mining	Reactor, electrical components, other plant components
Construction		
237130	Power and Communication Line and Related Structures Construction	All major structures
Specialty trade contractors		
238110	Poured concrete contractors	Containment, cooling tower, other concrete structures
238210	Electrical contractors and other wiring installation contractors	All major structures
238120	Structural steel and precast concrete contractors	All major structures
238220	Plumbing, heating, AC contractors	All major structures
238910	Site preparation contractors	Entire site
Manufacturing		
325180	Other basic inorganic chemical manufacturing	Fuel assemblies
325220	Artificial and synthetic fibers and filaments manufacturing	Door and equipment seals, electrical/thermal insulation
327320	Ready mix concrete manufacturing	All major structures
331110	Iron/steel mills, ferroalloy manufacturing	All major plant components
331210	Iron steel pipes and tubes manufacturing	All major plant components
331313	Alumina refining	All major plant components
331314	Alumina secondary smelting	All major plant components
331410	Non-ferrous (except Aluminum) metal melting and refining	Reactor, wires, cables
331492	Secondary smelting, refining, and alloying of nonferrous metal	Reactor, wires, cables
332312	Fabricated structural metal manufacturing	Concrete reinforcements (e.g., containment, cooling tower)
332313	Plate work manufacturing	Pressure vessel, other plant components
332410	Power boiler/heat exchanger manufacturing	Reactor building
332420	Metal tank (heavy gauge) manufacturing	Nuclear waste casks
332811	Metal heat treating	Pressure vessel, piping, turbine and compressor components
332911	Industrial valve manufacturing	All major structures
333415	Air-Conditioning and warm air heating equipment and commercial and industrial refrigeration equipment manufacturing	All major structures
333611	Turbine and turbine generator set units manufacturing	Steam generator

Table S-12: **Industries and services required for manufacturing and deploying the components of nuclear fission power plants, listed in ascending order based on 3-digit NAICS codes, Related to STAR Methods.** The list is based on the meta-analysis of nuclear fission lifecycle inventories conducted in[54] and the resulting inventory for an average pressurized water reactor representative of global production in 2020.

NUCLEAR FISSION PLANTS	(PART 2)	
**NAICS Code**	**Industry**	**Component/installation step**
Manufacturing, continued from Table S-12		
333612	Speed changer, industrial high-speed drive, and gear manufacturing	Generator, substation
334413	Semiconductor and related device manufacturing	Controls in all major structures
334417	Electrical connector manufacturing	Electrical components
334418	Printed circuit assembly manufacturing	Controls
334515	Instrument manufacturing for measuring and testing electricity and electrical signals	Detectors, sensors, and meters in all major plant components
335931	Current-carrying wiring device manufacturing	Wires, cables
333912	Air and gas compressor manufacturing	Reactor building
333914	Measuring, dispensing, and other pumping equipment manufacturing	All plant components
334412	Bare circuit board manufacturing	All plant components
334413	Semiconductor and related device manufacturing	All plant components
334417	Electrical connector manufacturing	All plant components
334418	Printed circuit assembly manufacturing	All plant components
334515	Instrument manufacturing for measuring and testing electricity and electrical signals	All plant components
335311	Power, distribution, and specialty transformer manufacturing	Step-up transformers for plant facilities; main substation
335312	Motor and generator manufacturing	Steam generator, emergency Diesel generators
335313	Switchgear and switchboard apparatus manufacturing	Plant facilities and substation
Merchant wholesalers		
423690	Other electronic parts and equipment wholesalers	Electrical plant components
Transportation		
483111	Deep sea freight transportation	Project-/supplier-dependent, but likely more specialized equipment such as the reactor and steam generator
484122	General freight trucking	All components
Warehousing		
493110	Warehousing and storage	All plant components, construction materials

Table S-13: **Industries and services required for manufacturing and deploying the components of nuclear fission power plants, listed in ascending order based on 3-digit NAICS codes, Related to STAR Methods.** The list is based on the meta-analysis of nuclear fission lifecycle inventories conducted in.[54]

NUCLEAR FISSION PLANTS	(PART 3)	
**NAICS Code**	**Industry**	**Component/installation step**
Finance and insurance		
522110	Commercial banking	Plant financing
Professional services		
541310	Architectural services	Reactor and other building design
541330	Engineering services	Plant design, operation, maintenance
541350	Building inspection services	Construction quality control, safety inspections, maintenance
541360	Geophysical services	Seismic assessment
561210	Facilities support services	Plant operation, waste storage facility operation
561612	Security guard, patrol services	See above
Administrative support and waste management remediation services		
562111	Solid waste collection	Collection of building materials
562112	Hazardous waste collection	Spent fuel collection; wastes from spent fuel reprocessing;
562212	Solid waste landfill	Solid materials after decontamination if needed[55]
562213	Solid Waste Combustors and Incinerators	Operational wastes (tools, clothing),[56] solid materials after decontamination if needed.[55]

Table S-14: **Industries and services required for manufacturing and deploying the components of nuclear fission power plants, listed in ascending order based on 3-digit NAICS codes, Related to STAR Methods.** The list is based on the meta-analysis of nuclear fission lifecycle inventories conducted in.[54]

CLEAN COOKSTOVES		
**NAICS Code**	**Industry**	**Component/installation step**
Agriculture		
115112	Soil preparation, planting, and	Fuelwood production
cultivating		
113210	Forest nurseries and gathering of forest products	Fuelwood production
Mining		
212230	Copper, nickel, lead, and zinc mining	Battery collector foil and connections, cable
212291	Iron ore mining	Steel body/stove top/base
212299	All other metal ore mining (bauxite, etc.)	Battery (Aluminum foil, film)
212395	Clay and ceramic and refractory minerals mining	Burning chamber
212393	Other chemical and fertilizer mineral mining	Battery (lithium)
212399	All other nonmetallic mineral mining	Battery (graphite cathode)
Manufacturing		
325199	All other basic organic chemical manufacturing	Battery (dimethyl and ethyl methyl carbonate)
327120	Clay building material and refractories manufacturing	Burning chamber
331110	Iron/steel mills, ferroalloy manufacturing	Steel body/stove top/base
331313	Alumina refining	Battery (Aluminum foil, film)
331314	Alumina secondary smelting	See above
332111	Iron and steel forging	Steel body/stove top/base
332215	Metal kitchen cookware, utensil, cutlery, and flatware manufacturing	All components
335912	Primary battery manufacturing	LiFePO4 battery
Merchant wholesalers		
423620	Household appliances, electric housewares, and consumer electronics merchant wholesalers	
Transportation		
484122	General freight trucking, long-distance	Component shipping
Warehousing		
493110	Warehousing and storage	Component storage
Administrative support and waste management remediation services		
562111	Solid waste collection	Collection of panels, inverter
562112	Hazardous waste collection	
562212	Solid waste landfill	
562213	Solid Waste Combustors and Incinerators	

Table S-15: **Industries and services required for manufacturing and deploying the components of a clean cookstove of the type ACE-1, Related to STAR Methods.** Components are for an Ultra-Clean Biomass Cookstove manufactured by Africa Clean Energy, see components and materials in the user manual[57] and in[58] for the battery), listed in ascending order based on 3-digit NAICS codes.

SDG6	PV SYSTEMS			Local manufacturing
**NAICS Code**	**Industry**	**Indicator**	**Linkage**	**Explanation and Reference**
Mining				
212230	Copper mining	6.1.1	0	No direct link to the
			0	management of drinking water.
		6.2.1	0	No direct link to the
			0	management of drinking water.
		6.3.1	-1	Water pollution from copper mining is well documented.[59,60] Extent depends on wastewater regulations/compliance.
		6.3.2	-1	Copper mining and smelting has been linked to water acidification and contamination (e.g.,[61]).
		6.4.1	-1	Industrial water uses exhibit below average water use efficiency, and services above average efficiency[14] . We therefore assign a 1 to services and a -1 to all other sectors mentioned.
		6.4.2	-1	Groundwater levels are in decline in many copper mining regions.[62,63]
212299	All other metal ore mining	6.3.1	-1	High alkalinity of red mud from bauxite mining can cause pollution of local water bodies (e.g.,[64]). Extent depends on local regulations/compliance.
		6.3.2	-1	Bauxite/alumina mining has been shown to affect freshwater ecotoxicity (through emissions to water) and water resource depletion.[65]
		6.4.2	-1	Bauxite/alumina mining has been shown to affect freshwater ecotoxicity (through emissions to water) and water resource depletion.[65]
212322	Industrial sand mining	6.3.1	-1	Sand mining can affect iron and heavy metal concentration in local water bodies[66] unless wastewater safely treated.
Mining				
212322	Industrial sand mining	6.3.2	-1	Sand mining has been shown to cause saltwater intrusion, heavy metal leaching and acid mine drainage, all related to water supply damages.[67,68,69,66]
		6.4.1	-1	Industrial water uses exhibit below average and services above average water use efficiency. [14] We therefore assign a '1' to services and a '-1' to all other sectors mentioned.
		6.4.2	-1	Sand mining has been linked to the depletion of groundwater levels[66]

Table S-16: **References for linkages between PV industries and SDG6 indicators in the local manufacturing scenario, Related to STAR Methods.** The same references are used in the import scenario, except that linkages between manufacturing industries and indicators are set to zero because technologies are assumed to be manufactured outside the boundary of the region for which SDG linkages are evaluated.

SDG6	PV SYSTEMS			Local manufacturing
**NAICS Code**	**Industry**	**Indicator**	**Linkage**	**Explanation, References**
Power generation				
221113	Solar electric power generation	6.4.1	-1	PV water usage efficiency is below average water usage efficiency in most countries.[14,70]
Construction				
237130	Power and communication line and related structures construction	All SDG6 indicators	0	No impact since water consumption during PV installation is zero[70]
Specialty trade contractors				
238210	Electrical contractors	6.4.1	1	Contractors are service providers, and services exhibit above-average water use efficiency in most countries[14]
238910	Site preparation contractors	6.4.1	1	Contractors are service providers, and services exhibit above-average water use efficiency in most countries[14]
Manufacturing				
331313, 331314	Alumina refining, alumina secondary smelting	6.3.1	-1	Alumina has been shown to affect freshwater ecotoxicity.**Farjana2019impac**
		6.3.2	-1	Alumina smelting and refining has been shown to affect freshwater ecotoxicity.[65]
		6.4.1<br>6.4.2	-1<br>-1	Industrial water uses exhibit below average and services above average water use efficiency. [14] We therefore assign a '1' to services and a '-1' to all other sectors mentioned.
325211	Plastics material and resins manufacturing	6.3.1<br>6.3.2<br>6.4.1<br>6.4.2	-1<br>-1<br>-1<br>-1	Plastics manufacturing has been linked to water contamination (e.g., Bisphenol A[71]). Same treatment as other manufacturing industries for 6.4.1/6.4.2 (see above).
327211	Flat glass manufacturing	6.3.1<br>6.3.2<br>6.4.1<br>6.4.2	-1<br>-1<br>-1<br>-1	Glass industry wastewaters can contain glass splinters, silica particles, and oil residues[72,73] and the glass industry has been linked to water contamination.[74] Same treatment as other manufacturing industries for 6.4.1/6.4.2 (see SI Table S-17).

Table S-17: **References for linkages between PV industries and SDG6 indicators in the local manufacturing scenario, Related to STAR Methods.** The same references are used in the import scenario, except that linkages between manufacturing industries and indicators are set to zero because technologies are assumed to be manufactured outside the boundary of the region for which SDG linkages are evaluated.

SDG6	PV SYSTEMS			Local manufacturing
**NAICS Code**	**Industries**	**Indicator**	**Linkage**	**Explanation, References**
Manufacturing				
334412, 334413, 334417, 334515 335931	Bare circuit board manufacturing, Semiconductor and related device manufacturing (includes solar cells), Electrical connector manufacturing, Instrument manufacturing, Current-carrying wiring device manufacturing	6.3.1 6.3.2	-1	Electrical equipment manufacturing can pose water quality risks due to increased disposal of copper wastewater containing treatment sludge from circuit board etching.[75]
		6.4.1	-1	Industrial water uses exhibit below and services above average water use efficiency. [14] We therefore assign a '1' to services and a '-1' to all other sectors.
		6.4.2	-1	Circuit boards and instruments contain semiconductors, which require significant water withdrawals and can cause water stress.[76] Computer electronics and el. equipment manufacturing are among the most water-consuming industries.[77]
334418	Printed circuit assembly	6.3.1 6.3.2 6.4.2	0 0	Assembly itself does not require water usage.
		6.4.1	-1	Same as other industries
Merchant wholesalers				
423690	Other electronic parts and equipment wholesalers	6.4.1	1	Wholesalers are service providers; services exhibit above-average water use efficiency in most countries [14] (see SI section S-6).
Transportation				
483111,484122	Deep sea freight transportation, General freight trucking	6.4.1	1	Transportation is a service (see above)
Finance and insurance				
522110	Commercial banking	6.4.1	1	Banks provide services.
Professional services				
541310, 541330, 541350	Architectural services, Engineering services, Building inspection services	6.4.1	1	Same treatment as all services (see above).
All PV industries				
See list in SI Table S-9		6.5.1	0	No documented link to integrated water management.
		6.5.2	0	No documented link to water cooperation.
		6.6.1	0	No documented link to changes in the spatial extent of water bodies.
		6.a.1	0	No documented link to development assistance.
		6.b.1	0	No documented link to policy focus on water management.

Table S-18: **References for linkages between PV industries and SDG6 indicators in the local manufacturing scenario, Related to STAR Methods.** The same references are used in the import scenario, except that linkages between manufacturing industries and indicators are set to zero because technologies are assumed to be manufactured outside the boundary of the region for which SDG linkages are evaluated.

SDG6	WIND (Part 1)			Local manufacturing
**NAICS Code**	**Industries**	**Indicator**	**Linkage**	**Explanation, References**
Mining				
212312, 212321	Crushed and broken limestone mining and quarrying; Construction sand and gravel mining;	6.1.1<br>6.2.1	0<br>0	No direct link to the management of drinking water or to the availability of sanitation services.
		6.4.1	-1	See SI section S-6
212312		6.3.1	-1	Water pollution has been linked to limestone mining in several countries.[78,79,80]
		6.4.2	-1	Limestone mining has been associated with water scarcity (e.g., streams become seasonal rather than perennial because they are intercepted by mines.[81]
212321		6.3.1	-1	Sand mining has been shown to cause saltwater intrusion as well as heavy metal leaching and acid mine drainage, which are all related to water supply damages[67,69,66,68]
		6.4.2	-1	Sand mining has been linked to depletion of groundwater levels.[66]
Manufacturing				
325220	Artificial and Synthetic Fibers and Filaments Manufacturing	6.3.1<br>6.3.2	0<br>0	Water is used for fiber washing and cooling,[82] but no evidence found on related wastewater issues.
		6.4.1	-1	Same treatment as all manufacturing industries (see SI section S-6).
		6.4.2	-1	High energy intensity of carbon fiber production compared to conventional metals[83] means that fiber manufacturing can lead to water stress.
327320	Ready-Mix Concrete Manufacturing	6.3.1<br>6.3.2	-1<br>-1	Cement manufacturing has been associated with water pollution (e.g.,[84]).

Table S-19: **References for linkages between wind turbine industries and SDG6 indicators in the local manufacturing scenario, Related to STAR Methods.** Linkages for industries and services required for both PV systems and wind turbines are not repeated in this table (see PV tables). These industries include: Copper mining (212210), all other metal ore mining (212299), alumina refining (331313), alumina secondary smelting (331314), bare circuit board manufacturing (334412), semiconductor and related device manufacturing (334413), electrical connector manufacturing (334417), printed circuit assembly (334418), instrument manufacturing for measurement (334515), current-carrying wiring device manufacturing (335931), power and communications line construction (237130), electrical trade contractors (238210), site preparation contractors (238910), engineering services (541330), deep sea freight transportation (483111), general freight trucking (484122), other electronic parts and equipment wholesalers (423690), and commercial banking (522110). The same references are used in the import scenario, except that linkages between manufacturing industries and indicators are set to zero because technologies are assumed to be manufactured outside the boundary of the region for which SDG linkages are evaluated.

SDG6	WIND (Part 2)			Local manufacturing
**NAICS Code**	**Industries**	**Indicator**	**Linkage**	**Explanation, References**
Manufacturing				
331410, 331492	Non-ferrous (except Aluminum) metal smelting and refining; Secondary Smelting, Refining, and Alloying of Nonferrous Metal (except Copper and Aluminum)	6.3.1<br>6.3.2	-1<br>-1	Alumina smelting and refining has been shown to affect freshwater ecotoxicity.[65]
		6.4.1	-1	Same treatment as all manufacturing industries (see SI section S-6).
		6.4.2	-1	All types of metals can cause water contamination and scarcity during mining, processing, and smelting.[61]
		All other SDG6 indicators	0	
332111, 332312, 332313 332811	Iron and steel forging; Fabricated structural metal manufacturing; Plate work manufacturing; Metal heat treating;	6.3.1<br>6.3.2	-1<br>-1	Iron and steel industries can cause significant water pollution (mainly nitrogen, iron, and chromium[85]).
		6.4.1	-1	Same treatment as all manufacturing industries (see SI section S-6).
		6.4.2	-1	Iron and steel industries contribute significantly to industrial water withdrawals.[86] Overall, the manufacturing industry accounts for over 20% of global freshwater withdrawals.[87]
		All other SDG6 indicators	0	

Table S-20: **References for linkages between wind turbine industries and SDG6 indicators in the local manufacturing scenario, Related to STAR Methods.** Linkages for industries and services required for both PV systems and wind turbines are not repeated in this table (see PV tables). These industries include: Copper mining (212210), all other metal ore mining (212299), alumina refining (331313), alumina secondary smelting (331314), bare circuit board manufacturing (334412), semiconductor and related device manufacturing (334413), electrical connector manufacturing (334417), printed circuit assembly (334418), instrument manufacturing for measurement (334515), current-carrying wiring device manufacturing (335931), power and communications line construction (237130), electrical trade contractors (238210), site preparation contractors (238910), engineering services (541330), deep sea freight transportation (483111), general freight trucking (484122), other electronic parts and equipment wholesalers (423690), and commercial banking (522110). The same references are used in the import scenario, except that linkages between manufacturing industries and indicators are set to zero because technologies are assumed to be manufactured outside the boundary of the region for which SDG linkages are evaluated.

SDG6	WIND (Part 3)			Local manufacturing
**NAICS Code**	**Industries**	**Indicator**	**Linkage**	**Explanation, References**
Manufacturing				
333611, 333612	Turbine and Turbine Generator Set Units Manufacturing; Speed Changer, Industrial High-Speed Drive, and Gear Manufacturing	6.3.1<br>6.3.2	-1<br>-1	Machinery manufacturing generates polluted wastewater (e.g.,.[88] Water treatment will depend on local regulations and compliance.
		6.4.1	-1	Same treatment as all manufacturing industries (see SI section S-6).
		6.4.2	-1	Manufacturing industry accounts for more than 20% of global freshwater withdrawals.[87]
335311	Power, Distribution, and Specialty Transformer Manufacturing	6.3.1<br>6.3.2	-1<br>-1	Transformer manufacturing can cause heavy metal contamination of water bodies.[89]
		6.4.1	-1	Same treatment as all manufacturing industries (see SI section S-6).
		6.4.2	-1	Manufacturing industry accounts for more than 20% of global freshwater withdrawals.[87]
Power generation				
221115	Wind electric power generation	6.4.1	1	The water usage efficiency of wind turbines (approximately 500 USD/m^3, see SI section S-6.1.1) is significantly higher than the average water usage efficiency across countries, which ranged from 1 (Madagascar) and 262 (Denmark) USD/m^3 in 2018.[14,70]
		All other SDG6 indicators	0	
Construction				
237130	Power and communication line and related structures construction	All SDG6 indicators	0	No impact since water consumption during wind turbine construction is zero.[70]

Table S-21: **References for linkages between wind turbine industries and SDG6 indicators in the local manufacturing scenario, Related to STAR Methods.** Linkages for industries and services required for both PV systems and wind turbines are not repeated in this table (see PV tables). These industries include: Copper mining (212210), all other metal ore mining (212299), alumina refining (331313), alumina secondary smelting (331314), bare circuit board manufacturing (334412), semiconductor and related device manufacturing (334413), electrical connector manufacturing (334417), printed circuit assembly (334418), instrument manufacturing for measurement (334515), current-carrying wiring device manufacturing (335931), power and communications line construction (237130), electrical trade contractors (238210), site preparation contractors (238910), engineering services (541330), deep sea freight transportation (483111), general freight trucking (484122), other electronic parts and equipment wholesalers (423690), and commercial banking (522110). The same references are used in the import scenario, except that linkages between manufacturing industries and indicators are set to zero because technologies are assumed to be manufactured outside the boundary of the region for which SDG linkages are evaluated.

SDG6	WIND (Part 4)			Local manufacturing
**NAICS Code**	**Industries**	**Indicator**	**Linkage**	**Explanation, References**
Specialty trade contractors				
238110	Poured Concrete Foundation and Structure Contractors	6.4.1	1	Contractors are service providers, and services exhibit above-average water use efficiency in most countries[14]
		All other SDG6 indicators	0	
Merchant wholesalers				
423320	Brick, Stone, and Related Construction Material Merchant Wholesalers	6.4.1	1	Wholesalers are service providers. Services exhibit above-average water use efficiencies[14] (see SI section S-6)
Professional services				
541360	Geophysical surveying	6.4.1	1	Same treatment as other engineering services (see table S-18)
All wind industries				
		6.5.1	0	No direct link to water resource management.
		6.5.2	0	No direct link to water cooperation arrangements.
		6.6.1	0	Would require a link between contamination and amount of water in ecosystems plus overall spatial extent of water ecosystems,[90] which is not well established.
		6.A.1	0	No direct link of industry/service type to water development assistance.
		6.B.1	0	No direct link of industry/service type to operational policies and procedures.

Table S-22: **References for linkages between wind turbine industries and SDG6 indicators in the local manufacturing scenario, Related to STAR Methods.** Linkages for industries and services required for both PV systems and wind turbines are not repeated in this table (see PV tables). These industries include: Copper mining (212210), all other metal ore mining (212299), alumina refining (331313), alumina secondary smelting (331314), bare circuit board manufacturing (334412), semiconductor and related device manufacturing (334413), electrical connector manufacturing (334417), printed circuit assembly (334418), instrument manufacturing for measurement (334515), current-carrying wiring device manufacturing (335931), power and communications line construction (237130), electrical trade contractors (238210), site preparation contractors (238910), engineering services (541330), deep sea freight transportation (483111), general freight trucking (484122), other electronic parts and equipment wholesalers (423690), and commercial banking (522110). The same references are used in the import scenario, except that linkages between manufacturing industries and indicators are set to zero because technologies are assumed to be manufactured outside the boundary of the region for which SDG linkages are evaluated.

SDG6	NUCLEAR (Part 1)			Local manufacturing
**NAICS Code**	**Industries**	**Indicator**	**Linkage**	**Explanation, References**
Mining				
212291	Uranium-radium-vanadium ore mining	6.1.1	0	Same treatment as
		6.2.1	0	PV (see S-16.
		6.3.1	-1	Uranium mining has been associated with radionucleide and metal contamination in surface and groundwater in the US and China.[91,92]
		6.4.1	-1	See SI section S-6
		6.4.2	-1	Uranium mining and enrichment is similarly water intensive as coal mining.[93]
Manufacturing				
325180	Other basic inorganic chemical manufacturing	6.3.1	-1	Fuel fabrication causes only a small fraction of overall emissions from the nuclear fuel cycle, but fuel fabrication plants generate radioactive and chemically polluted wastewater[94,95]
		6.3.2	-1	Nuclear fuel fabrification can contribute to freshwater eutrophication[54]
		6.4.1	-1	See SI section S-6
		6.4.2	-1	Fuel fabrication processes use water for converting uranium hexafluoride or uranium trioxide to uranium dioxide and therefore contribute to total water use per kWh.[96,54]
331110, 331210, 332911 333415, 333912, 333914 335312, 335313	Iron/steel mills, ferroalloy manufacturing; Iron steel pipes and tubes manufacturing; Industrial valve manufacturing; Air-conditioning and warm air heating equipment and commercial and industrial refrigeration equipment manufacturing; Air and gas compressor manufacturing; Measuring, Dispensing, and other pumping equipment manufacturing; Motor and generator manufacturing; Switchgear and switchboard apparatus manufacturing;	6.3.1	-1	The iron and steel industry is a significant source of water contamination and stress due to water use for heat.[97]
		6.3.2	-1	
		6.4.1	-1	Same treatment as all manufacturing industries (see SI section S-6).
		6.4.2	-1	The manufacturing industry accounts for ¿20% of global freshwater withdrawals[87] and can therefore contribute to water stress.

Table S-23: **References for linkages between nuclear fission industries and SDG6 indicators in the local manufacturing scenario, Related to STAR Methods.** Linkages for industries and services required for both nuclear, PV systems and wind turbines are not repeated in this table (see PV/wind tables).The same references are used in the import scenario, except that linkages between manufacturing industries and indicators are set to zero because technologies are assumed to be manufactured outside the boundary of the region for which SDG linkages are evaluated.

SDG6	NUCLEAR (Part 2)			Local manufacturing
**NAICS Code**	**Industries**	**Indicator**	**Linkage**	**Explanation, References**
221113	Nuclear electric power generation	6.4.1	country-specific	The water usage efficiency of nuclear power generation (approximately 55 USD/m^3, see SI section S-6.1.1) is higher than the average global water usage efficiency in 2018 (43 USD/m^3). Given the wide range overall (from 1 USD/m^3 in Madagascar to 262 in Denmark[14,70]), decisions on potential linkages
		All other SDG6 indicators	0	should be made on a country-by-country basis (see SI section S-6.1.1 for country-specific assumptions.
Specialty trade contractors				
238120, 238220	Structural steel and precast concrete contractors; Plumbing, heating, AC contractors;	6.4.1	1	Contractors are service providers, and services exhibit above-average water use efficiency in most countries[14]
		All other SDG6 indicators	0	
Merchant wholesalers				
423690	Other electronic parts and equipment wholesalers	6.4.1	1	Wholesalers are service providers. Services exhibit above-average water use efficiencies[14] (see SI section S-6)
All nuclear industries				
		6.5.1	0	No direct link to water resource management.
		6.5.2	0	No direct link to water cooperation arrangements.
		6.6.1	0	Would require a link between contamination and amount of water in ecosystems plus overall spatial extent of water ecosystems,[90] which is not well established.
		6.A.1	0	No direct link of industry/service type to water development assistance.
		6.B.1	0	No direct link of industry/service type to operational policies and procedures.

Table S-24: **References for linkages between nuclear fission plant industries and SDG6 indicators in the local manufacturing scenario, Related to STAR Methods.** Linkages for industries and services required for both nuclear fission plants, PV systems and wind turbines are not repeated in this table (see PV tables). The same references are used in the import scenario, except that linkages between manufacturing industries and indicators are set to zero because technologies are assumed to be manufactured outside the boundary of the region for which SDG linkages are evaluated.

SDG6	COOKSTOVES (Part 1)			Local manufacturing
**NAICS Code**	**Industries**	**Indicator**	**Linkage**	**Explanation, References**
Mining				
212325	Clay and ceramic and refractory minerals mining	6.1.1	0	Same treatment as PV (see S-16.
		6.2.1	0	
		6.3.1	-1	Clay mining has been linked to river water contamination through atmospheric mining dust deposition in the Americas,[98] India,[99] and in the UK.[100]
		6.4.1	-1	Same treatment as other manufacturing industries (see SI section S-6).
		6.4.2	-1	Clay mining has been linked to a lowering of water tables in India[101] and Sri Lanka.[102]
212393	Other chemical and fertilizer mineral mining	6.3.1	-1	Lithium ion mining is associated with water contamination.[103]
		6.3.2	-1	
		6.4.1	-1	Same treatment as other manufacturing industries (see SI section S-6).
		6.4.2	-1	Lithium mining is a water-intensive industry that is associated with water shortages.[104]
Manufacturing				
327120	Clay building material and refractories manufacturing;	6.3.1	0	No major hazardous pollutants in ceramics industry wastewaters.[105]
		6.3.2	0	
		6.4.1	-1	Tile manufacturing is a water-intensive process, consuming appr. 20 l/m^2.[106]
		6.4.2	-1	
332215	Metal kitchen cookware, utensil, cutlery, and flatware (except precious) manufacturing;	6.3.1	0	No evidence found on water contamination.
		6.3.2	0	
		6.4.1	-1	Same treatment as all manufacturing industries (see SI section S-6).
335912	Primary battery manufacturing;	6.3.1	0	Lithium-ion battery manufacturing is not associated with water pollution (mining impacts are accounted for under Mining (212393)).
		6.3.2	0	
		6.4.1	-1	Same treatment as all manufacturing industries (see SI section S-6). Overall, the manufacturing industry accounts for >20% of global freshwater withdrawals[87] and can therefore contribute to water stress.

Table S-25: **References for linkages between cookstove industries and SDG6 indicators in the local manufacturing scenario, Related to STAR Methods.** Linkages for industries and services required for both nuclear, PV systems and wind turbines are not repeated in this table (see PV/wind tables).The same references are used in the import scenario, except that linkages between manufacturing industries and indicators are set to zero because technologies are assumed to be manufactured outside the boundary of the region for which SDG linkages are evaluated.

SDG6	COOKSTOVES (Part 2)			Local manufacturing
**NAICS Code**	**Industries**	**Indicator**	**Linkage**	**Explanation, References**
Agriculture				
113210, 115112	Forest nurseries and gathering of forest products; Soil preparation, planting, and cultivating	6.3.1<br>6.3.2	-1<br>-1	Woodfuel production can contribute to eutrophication and acidification, and wastewater can play a role.[107] consuming appr. 20 l/m^2.[106]
		6.4.1	-1	Same treatment as all industries.
		6.4.2	-1	Woodfuel production can affect water stress through changes in water tables.[107]
		All other SDG6 indicators	0	
Merchant wholesalers				
423620	Household appliances, electric housewares, and consumer electronics merchant wholesalers	6.4.1	1	Same treatment as other services (see SI section S-6).
		All other SDG6 indicators	0	

Table S-26: **References for linkages between cookstove industries and SDG6 indicators in the local manufacturing scenario, Related to STAR Methods.** Linkages for industries and services required for both nuclear, PV systems and wind turbines are not repeated in this table (see PV/wind tables).The same references are used in the import scenario, except that linkages between manufacturing industries and indicators are set to zero because technologies are assumed to be manufactured outside the boundary of the region for which SDG linkages are evaluated.

SDG8 (PART 1)	PV SYSTEMS			Local manufacturing
**NAICS Code**	**Industries**	**Indicator**	**Linkage**	**Explanation, References**
All PV industries				
212230, 212299, 212322 221113, 237130, 331313 331314, 334417,334515 335931, 334412, 334413 334418,334515, 335931 493110	See Table x for list of all PV industries	8.1.1 8.2.1	1 1	Mining and manufacturing growth are positively associated with economic growth in developing countries,[**haraguchi2017importance**, 108] b relationship weakens as economies industrialise (inverse u-shape[109]). Manufacturing contributes to economic growth due to the sector's higher productivity and potential for productivity growth compared to sectors like agriculture, as well as higher potential for capital aggregation
238210, 238910, 483111 484122, 423690, 522110 541310, 541330, 541350		8.1.1 8.1.2	1 1	Services also contribute to economic growth,[110,109] particularly when growth is high.[109]
Multiple PV industries				
237130, 238210, 238910 423690, 483111, 484122 493110, 522110, 541310 541330, 541350	Power and Communication Line and Related Structures Construction; Electrical contractors; Site preparation contractors; Other electronic parts wholesalers; Deep sea freight transportation; General freight trucking; Warehousing and storage; Commercial banking; Architectural services; Engineering services; Building inspection services;	8.3.1	1	Solar PV and distributed generation more broadly has been reported to create twice as many informal as formal jobs in India, Kenia, and Nigeria.[1,111] These jobs exist mostly in commerce and communications,[1] not manufacturing; we therefore assign '1' here for all non-manufacturing PV industries; and '0' for other PV industries
Multiple PV industries				
212230, 212299, 212322 325211, 331313, 331314 334412, 334413, 334417 334515, 335931, 483111 484122	Copper mining; All other metal ore mining; Industrial sand mining; Plastics material and resins manufacturing; Alumina refining; Alumina secondary smelting; Bare circuit board manufacturing; Semiconductor and related device manufacturing; Electrical connector manufacturing; Instrument manufacturing; Current-carrying wiring device manufacturing; Deep sea freight transportation; General freight trucking	8.4.1 8.4.2	-1 -1	According to the UN's 2021 indicator meta-data[112] the total material footprint is the sum of the material footprint for biomass, fossil fuels, metal ores and non-metal ores. Materials footprint is defined as the footprint in kg per units of constant USD of final demand. We therefore assign a '1' to all industries requiring direct use of substantial amounts of materials or fuels. E.g., warehousing is assigned '0' because the materials and fuels embodied in the stored products is already accounted for in manufacturing industries.

Table S-27: **References for linkages between PV industries and SDG8 indicators in the local manufacturing scenario, Related to STAR Methods.** The same references are used in the import scenario, except that linkages between manufacturing industries and indicators are set to zero because technologies are assumed to be manufactured outside the boundary of the region for which SDG linkages are evaluated.

SDG8 (PART 2)	PV SYSTEMS			Local manufacturing
**NAICS Code**	**Industries**	**Indicator**	**Linkage**	**Explanation, References**
Multiple PV industries				
212230, 212299, 212322 238910, 325180, 331313 331314, 334412, 334417 334418, 334515, 423690 483111, 484122, 522110 541310, 541330, 541350	Copper mining; All other metal ore mining; Industrial sand mining; Site preparation contractors; Other basic inorganic chemical manufacturing; Bare circuit board manufacturing; Electrical connector manufacturing; Printed circuit assembly; Instrument manufacturing; Other electronic parts manufacturing; Deep sea freight transportation; General freight trucking; Commercial banking; Architectural services; Engineering services; Building inspection services;	8.5.1	0	These industries are not among the clean energy production industries with above average earnings in the countries where information specific to clean energy industries is available (e.g., U.S.[113]). A similar grouping of industries into above- and below-average paying can be observed in other countries where information specific to clean energy is not available (e.g., wages are above average for engineering-related manufacturing and electricity generation and below average for other manufacturing, retail services, and transportation[43]).
221113, 237130, 238210 334413, 335931	Solar electric power generation; Power and comm. line construction; Electrical contractors; Semiconductor and related device manufacturing; Current-carrying wiring device manufacturing;	8.5.1	1	These industries are listed as clean energy production industries with above average earnings in the countries where information specific to clean energy industries is available (e.g., for the U.S., see Table A1 in[113]).
All PV industries				
		8.5.2	1	All industries have the potential to contribute to reductions in the unemployment rate.
All PV industries				
		8.6.1	0	No reason to expect effects on youth employment. For countries where information specific to clean energy industries is available, average worker ages tend to be advanced (e.g., 42 y. in the U.S.[113]).

Table S-28: **References for linkages between PV industries and SDG8 indicators in the local manufacturing scenario, Related to STAR Methods.** The same references are used in the import scenario, except that linkages between manufacturing industries and indicators are set to zero because technologies are assumed to be manufactured outside the boundary of the region for which SDG linkages are evaluated.

SDG8 (PART 3)	PV SYSTEMS			Local manufacturing
**NAICS Code**	**Industries**	**Indicator**	**Linkage**	**Explanation, Refere**
Multiple PV industries				
212230, 212299, 212322 238910, 331313, 331314 334412, 334413, 334417 334418, 334515, 335931 423690, 483111, 484122 522110, 541310, 541330 541350	Copper mining; All other metal ore mining; Industrial sand mining; Site preparation contractors; Bare circuit board manufacturing; Semiconductor and related device manufacturing; Electrical connector manufacturing; Printed circuit assembly; Instrument manufacturing; Other electronic parts manufacturing; Deep sea freight transportation; General freight trucking; Commercial banking; Architectural services; Engineering services; Building inspection services;	8.7.1	-1	Mining, transport and storage, basic metals, and ICT&electronics a among the top industr in terms of child labor risk in two or more world regions according to an OECD report.[114] All other PV industries have been assigned '0' because they are not high-risk for child labo though regional supply chains may neverthele involve illegal labor.
Multiple PV industries				
212230, 212299, 212322 237130, 238210, 238910 325211, 331313, 331314 493110, 334412, 334413 334417, 334515, 335931 Plastics material 483111, 484122	Copper mining; All other metal ore mining; Industrial sand mining; Power & communication line construction; Electrical contractors; Site preparation contractors; and resins manufacturing; Alumina refining; Alumina secondary smelting; Bare circuit board manufacturing; Semiconductor and related device manufacturing; Electrical connector manufacturing; Instrument manufacturing; Current-carrying wiring device manufacturing; Deep Warehousing & storage;	8.8.1	-1<br>of non-fatal injury rates	Mining, manufacturing construction, and tran services are among the most dangerous industries in terms (U.S. data[115]).
All PV industries				
		8.8.2	0	No studies found in the available literatu on industry differences labour rights complian
All PV industries				
		8.9.1<br>8.9.2	0	No studies found on contributions of clean energy to GDP through tourism growth. (Renewable en have minor effects on tourism (e.g.,[116]) but there is currently no indication for GDP-sca
All PV industries				
		8.10.1<br>8.10.2	0	No documented effects of PV industry scaling on bank branch density or bank account ownershi

Table S-29: **References for linkages between PV industries and SDG8 indicators in the local manufacturing scenario, Related to STAR Methods.** The same references are used in the import scenario, except that linkages between manufacturing industries and indicators are set to zero because technologies are assumed to be manufactured outside the boundary of the region for which SDG linkages are evaluated.

SDG8 (PART 1)	WIND			Local manufacturing
**NAICS Code**	**Industries**	**Indicator**	**Linkage**	**Explanation, References**
All wind industries				
212312, 212321, 325220 327320, 331410, 331492 332111; 332312; 332313 332811;333611;333612	Crushed and broken limestone quarrying and mining; Construction sand and gravel mining; Artificial and Synthetic Fibers and Filaments Manufacturing; Ready Mix Concrete Manufacturing; Non-ferrous (except Aluminum) metal melting and refining; Iron and steel forging; Fabricated structural metal manufacturing; Plate work manufacturing; Metal heat treating; Turbine and Turbine Generator Set Units Manufacturing; Speed Changer, Industrial High-Speed Drive, and Gear Manufacturing	8.1.1 8.2.1	1 1	Mining and manufacturing growth are positively associated with economic growth in developing countries ,[**haraguchi2017importance**, 108] but the relationship weakens as economies industrialise (inverse u-shape[109]). Manufacturing contributes to economic growth due to the sector's higher productivity and potential for productivity growth compared to sectors like agriculture, as well as higher potential for capital aggregation.
221115, 238110, 423320 541360	Wind electric power generation; Poured Concrete Foundation and Structure Contractors; Brick, Stone, and Related Construction Material Merchant Wholesalers; Geophysical surveying;	8.1.1 8.1.2	1 1	Services also contribute to economic growth,[110,109] particularly when growth is high.[109]
All wind industries				
See full list in table xy		8.3.1	0	No studies found that link wind supply chain to informal employment (neither,[1] a multi-country summary, nor.[117] Jobs in the wind industry tend to be high-skill,[117] and thus in the formal economy.
Multiple wind industries				
212312, 212321, 325220 327320, 332312, 332313 332811, 333611, 333612 335311	Crushed and Broken Limestone Mining and Quarrying; Construction Sand and Gravel Mining; Artificial and Synthetic Fibers and Filaments Manufacturing; Ready-Mix Concrete Manufacturing; Fabricated structural metal manufacturing; Plate work manufacturing; Metal heat treating; Turbine and Turbine Generator Set Units Manufacturing; Speed Changer, Industrial High-Speed Drive, and Gear Manufacturing; Power, Distribution, and Specialty Transformer Manufacturing;	8.4.1 8.4.2	-1 -1	Same treatment as for PV industries (see SI Table S-29 for details). Here we list only wind industries not also required for PV.

Table S-30: **References for linkages between wind power industries and SDG8 indicators in the local manufacturing scenario, Related to STAR Methods.** The same references are used in the import scenario, except that linkages between manufacturing industries and indicators are set to zero because technologies are assumed to be manufactured outside the boundary of the region for which SDG linkages are evaluated.

SDG8 (PART 2)	WIND			Local manufacturing
**NAICS Code**	**Industries**	**Indicator**	**Linkage**	**Explanation, References**
Multiple wind industries				
221115, 333611, 335311	Wind electric power generation; Turbine and Turbine Generator Set Units Manufacturing; Power, Distribution, and Specialty Transformer Manufacturing;	8.5.1	1	Same treatment as PV (see SI Table S-28). These industries are listed as clean energy production industries with above average earnings in the countries where information specific to clean energy industries is available (e.g., U.S., see Table A1 in[113]). Here we list only industries not also required for PV.
All other wind industries			0	See directly above
All wind industries				
		8.5.2	1	All industries have the potential to contribute to reductions in the unemployment rate.
All wind industries				
		8.6.1	0	No reason to expect effects on youth employment. For countries where information specific to clean energy industries is available, average worker ages tend to be advanced (e.g., 42 y. in the U.S.[113]).
Multiple wind industries				
212312, 212321, 423320	Crushed and Broken Limestone Mining and Quarrying; Construction Sand and Gravel Mining; Brick, Stone, and Related Construction Material Merchant Wholesalers;	8.7.1	-1	Same treatment as PV industries (see SI Table S-29). Here we list only industries not also required for PV.
212312, 212321, 238110 423320	Crushed and Broken Limestone Mining and Quarrying; Construction Sand and Gravel Mining; Poured Concrete Foundation and Structure Contractors; Brick, Stone, and Related Construction Material Merchant Wholesalers	8.8.1	-1	Same treatment as PV industries (see SI Table S-29). Here we list only industries not also required for PV.
All wind industries				
		8.9.1<br>8.9.2<br>8.10.1<br>8.10.2	0<br>0<br>0<br>0	Same treatment as PV (see SI Table S-29)

Table S-31: **References for linkages between wind power industries and SDG8 indicators in the local manufacturing scenario, Related to STAR Methods.** The same references are used in the import scenario, except that linkages between manufacturing industries and indicators are set to zero because technologies are assumed to be manufactured outside the boundary of the region for which SDG linkages are evaluated.

SDG8 (PART 1)	NUCLEAR			Local manufacturing
**NAICS Code**	**Industries**	**Indicator**	**Linkage**	**Explanation, References**
All nuclear industries				
212291, 331210, 332410 332420, 332911, 333415 333912, 333914, 335312 335313	Uranium-radium-vanadium ore mining; Iron steel pipes and tubes manufacturing Power boiler/heat exchanger manufacturing Metal tank manufacturing; Industrial valve manufacturing; Air-conditioning and warm air heating equipment and commercial and industrial refrigeration equipment manufacturing; Air and gas compressor manufacturing; Measuring, dispensing, and other pumping equipment manufacturing; Motor and generator manufacturing; Switchgear and switchboard apparatus manufacturing;	8.1.1<br>8.2.1<br>8.4.1<br>8.4.2	1<br>1<br>-1<br>-1	Same treatment as for PV and wind. Here we list only industries not also required for PV and wind.<br>Same as above.
221113, 238110, 238120 238220, 561612	Nuclear electric power generation; Poured concrete contractors; Structural steel and precast concrete contractors; Plumbing, heating, AC contractors; Security guard, patrol services	8.1.1<br>8.1.2	1<br>1	Services also contribute to economic growth,[110,109] particularly when growth is high.[109]
All nuclear industries				
See full list in SI tables S-12 and S-13		8.3.1	0	Book by OECD and Nuclear Energy Agency on employment generated by nuclear industry in US, Korea, and France does not mention any informal employment.[118]
221113		8.5.1	1	Same treatment as PV and wind (see SI Table S-28) Here we only list nuclear-specific industries.
All nuclear industries				
		8.6.1	0	The median age of the clean energy production workforce is >42 years in the US,[113] so there is no reason to assume that scaling nuclear would would reduce youth unemployment.

Table S-32: **References for linkages between nuclear fission plant industries and SDG8 indicators in the local manufacturing scenario, Related to STAR Methods.** The same references are used in the import scenario, except that linkages between manufacturing industries and indicators are set to zero because technologies are assumed to be manufactured outside the boundary of the region for which SDG linkages are evaluated.

SDG8 (PART 2)	NUCLEAR			Local manufacturing
**NAICS Code**	**Industries**	**Indicator**	**Linkage**	**Explanation, References**
All nuclear industries				
212291, 331210, 423320	Uranium-radium-vanadium ore mining; Iron steel pipes and tubes manufacturing Brick, stone, and related construction material merchant wholesalers	8.7.1<br>8.2.1	-1<br>1	Same treatment as for PV and wind. Here we list only nuclear-specific industries
325180	Other basic inorganic chemical manufacturing	8.7.1	0	No evidence on child labor in nuclear fuel fabrication. Highly specialized and closely monitored facilities with little to no opportunity for informal/illegal labor.
		8.8.1	-1	Same treatment as other manufacturing industries.
212291, 238110, 238120 238220	Uranium-radium-vanadium ore mining; Poured concrete contractors; Structural steel and precast concrete contractors; Plumbing, heating, AC contractors	8.8.1	-1	Same treatment as PV and wind. Contractor industries are listed here because 'Construction' is the fourth most dangerous industry both by death and by non-fatal injury rate.[115]
	All other nuclear industries	8.8.1	0	
	All nuclear industries	8.8.2<br>8.9.1<br>8.9.2<br>8.10.1<br>8.10.2	0<br>0<br>0<br>0<br>0	No evidence based on which to assume a link.

Table S-33: **References for linkages between nuclear fission plant industries and SDG8 indicators in the local manufacturing scenario, Related to STAR Methods.** The same references are used in the import scenario, except that linkages between manufacturing industries and indicators are set to zero because technologies are assumed to be manufactured outside the boundary of the region for which SDG linkages are evaluated.

SDG8 (PART 1)	COOKSTOVES			Local manufacturing
**NAICS Code**	**Industries**	**Indicator**	**Linkage**	**Explanation, References**
Manufacturing industries				
212324, 212393, 327120<br>332215, 335912	Clay and ceramic and<br>refractory minerals mining;<br>Other chemical and<br>fertilizer mineral mining;<br>Clay building material<br>and refractories manufacturing<br>(includes ceramic tiles<br>manufacturing, fire brick<br>manufacturing, and liner<br>brick manufacturing)<br>Metal kitchen cookware,<br>utensil, cutlery, and flatware<br>(except precious) manufacturing;<br>Primary battery manufacturing	8.1.1<br>8.2.1<br>8.4.1<br>8.4.2<br><br>8.4.1<br>8.4.2	1<br>1<br>-1<br>-1<br><br>-1<br>-1	Same treatment as<br>for PV and wind. Here<br>we list only industries<br>not also required for<br>PV, wind, or nuclear.<br><br>Same as above.
423620,	Household appliances, electric<br>housewares, and consumer<br>electronics merchant wholesalers	8.1.1<br>8.1.2<br>8.3.1	1<br>1<br>1	Services also contribute to<br>economic growth,[110,109]<br>particularly when growth is<br>high.[109]
Agriculture				
		8.1.1<br>8.2.1	-1<br>-1	Agriculture is a comparatively<br>low-productivity sector,<br>associated with slower<br>growth.[119]
Agriculture				
113210, 115112	Forest nurseries and<br>gathering of forest products;<br>Soil preparation, planting,<br>and cultivating	8.5.1	-1	Agricultural worker make<br>less than half of<br>median wage in US[120]<br>and agriculture is also<br>a below average earnings<br>industry in other countries

Table S-34: **References for linkages between clean cookstove industries and SDG8 indicators in the local manufacturing scenario, Related to STAR Methods.** The same references are used in the import scenario, except that linkages between manufacturing industries and indicators are set to zero because technologies are assumed to be manufactured outside the boundary of the region for which SDG linkages are evaluated.

SDG8 (PART 2)	COOKSTOVES			Local manufacturing
**NAICS Code**	**Industries**	**Indicator**	**Linkage**	**Explanation, References**
332215	Metal kitchen cookware, utensil, cutlery, and flatware (except precious) manufacturing	8.6.1	1	This will be country-dependent, but youth participati formal (manufacturing) and info (logistics) jobs directly related to clean cooking is high, in the countries where data is available.[121]
113210, 115112	Forest nurseries and gathering of forest products; Soil preparation, planting, and cultivating			Agricultural workers have been reported to be relatively young (e.g., in the U.S., India[122,123]).
113210, 115112, 212324 212393, 423620	Forest nurseries and gathering of forest products; Soil preparation, planting, and cultivating; Clay and ceramic and refractory minerals mining; Other chemical and fertilizer minerals mining; Household appliances, electric housewares, and consumer electronics merchant wholesalers	8.7.1	-1	Country-dependent, but agriculture, mining, and wholesa are among the top industries by child labor risk in 3 out of 5 world regions in an OECD 2019 report (,[114] Table 1)
		8.8.1	-1	Agriculture and forestry is the most/2nd most dangerous industry in terms of death rate/non-fatal injury rate.[115] Mining is the second most dangerous industry by death rate and the 12th most dangerous industry by non-fatal industry rate[115] . Wholesale trade is the 5th most dangerous industry by death rate and the 8th most dangerous industry by illness/injury rate.[115]

Table S-35: **References for linkages between clean cookstove industries and SDG8 indicators in the local manufacturing scenario, Related to STAR Methods.** The same references are used in the import scenario, except that linkages between manufacturing industries and indicators are set to zero because technologies are assumed to be manufactured outside the boundary of the region for which SDG linkages are evaluated.

SDG9 (PART 1)	PV SYSTEMS			Local manufacturing
**NAICS Code**	**Industries**	**Indicator**	**Linkage**	**Explanation, References**
Multiple PV industries				
212230, 212299, 212322	Copper mining; All other metal ore mining; Industrial sand mining;	9.1.1	1	Sand, bauxite, and metal ore mines more broadly require access roads and road connections to railway stations and ports.[124,125,126,127]
212230, 212299, 212322, 331313, 331314, 334412, 334413, 334417, 334418 334515, 335931, 483111 484122, 493110	Copper mining; All other metal ore mining; Industrial sand mining; Alumina refining; Alumina secondary smelting; Bare circuit board manufacturing; Semiconductor and related device manufacturing; Electrical connector manufacturing; Printed circuit assembly; Instrument manufacturing for measurement; Current-carrying wiring device manufacturing; Deep sea freight; General freight trucking; Warehousing & storage	9.1.2	1	Mines are often far from receiver areas, and roads and railway are typical means of transportation for mining products (e.g.,[128]). Other indicators: Scaling of most industrial/commercial activities related to PV will require more PV components to be manufactured, stored, and transported.
237130	Power and communications line and related structures construction;	9.1.2	0	Power line component transportation is accounted for under the respective component industries.
238210, 238910, 522110 541310, 541330	Electrical contractors; Commercial banking; Site preparation contractors; Architectural services; Engineering services	9.1.2	0	PV industry scaling will only involve transportation of a limited number of professionals.
Multiple PV industries				
212230, 212299, 212322 237130; 238210; 238910; 493110;	Copper mining; All other metal ore mining; Industrial sand mining; Power & communication line construction; Electrical contractors; Site preparation contractors; Warehousing & storage;	9.2.1<br>9.2.2	0<br>0	Manufacturing is defined as including all industries involved in the physical or chemical transformation of materials, substances, or components into new products, (based on the International Standard Industrial Classification of All Economic Activities (ISIC), Rev. 4[129,130]). This definition does not include mining, contractor services, warehousing, or construction.

Table S-36: **References for linkages between PV industries and SDG9 indicators in the local manufacturing scenario, Related to STAR Methods.** The same references are used in the import scenario, except that linkages between manufacturing industries and indicators are set to zero because technologies are assumed to be manufactured outside the boundary of the region for which SDG linkages are evaluated.

SDG9 (PART 2)	PV SYSTEMS			Local manufacturing
**NAICS Code**	**Industries**	**Indicator**	**Linkage**	**Explanation, References**
Multiple PV industries				
331313, 331314, 334412 334413, 334417, 334418 334515, 335931	Alumina refining; Alumina secondary smelting; Circuit board manufacturing; Semiconductor and related; device manufacturing; Electrical connector manufacturing; Printed circuit assembly; Instrument manufacturing; Current-carrying wiring device manufacturing;	9.2.1<br>9.2.2	1<br>1	Manufacturing is defined as including all industries involved in the physical or chemical transformation of materials, substances, or components into new products, (based on the International Standard Industrial Classification of All Economic Activities (ISIC), Rev. 4[129,130]). This definition includes the manufacturing of basic metals, semiconductors, instruments, and electrical components.
Multiple PV industries				
212230, 212299, 212322 237130; 238210; 238910; 334413, 334417, 334418 334515, 335931, 483111 484122, 493110	Copper mining; All other metal ore mining; Industrial sand mining; Alumina refining; Alumina secondary smelting; Bare circuit board manufacturing; Semiconductor and related device manufacturing; Electrical connector manufacturing; Printed circuit assembly; Instrument manufacturing for measurement; Current-carrying wiring device manufacturing	9.3.1<br>9.3.2	0<br>0	Small-scale industries are defined as enterprises with 5-19 employees.[130] Industry-specific enterprise sizes are country-specific, but extraction companies tend to be larger.[131] In many OECD countries, the service sector includes a factor 5-10 more small-scale enterprises than the manufacturing and construction sectors (see SI section S-7 for details). We therefore assign a '1' to all service industries and a '0' to all manufacturing and construction industries. We treat 9.3.1 and 9.3.2 equally because most businesses have loans or lines of credit.
		9.3.1<br>9.3.2	1<br>1	See explanation directly above.

Table S-37: **References for linkages between PV industries and SDG9 indicators in the local manufacturing scenario, Related to STAR Methods.** The same references are used in the import scenario, except that linkages between manufacturing industries and indicators are set to zero because technologies are assumed to be manufactured outside the boundary of the region for which SDG linkages are evaluated.

SDG9 (PART 3)	PV SYSTEMS			Local manufacturing
**NAICS Code**	**Industries**	**Indicator**	**Linkage**	**Explanation, Referen**
Multiple PV industries				
212230, 212299, 212322 237130; 238210; 238910; 325211, 334413, 334417 334418, 334515, 335931 483111, 484122, 493110	Copper mining; All other metal ore mining; Industrial sand mining; Plastics material and resin manufacturing; Alumina refining; Alumina secondary smelting; Bare circuit board manufacturing; Semiconductor and related device manufacturing; Electrical connector manufacturing; Printed circuit assembly; Instrument manufacturing for measurement; Current-carrying wiring device manufacturing	9.4.1	-1	In 73% of countries and years for which data is available from the Unit Nations Economic Commission for Europe ,[132,133] manufacturing value is more CO_2-intensive than average value added in tons of CO_2 per GDP, and service value less CO_2-intensive (assuming tot CO_2 per value added is the sum of manufacturing- and service- related CO_2). The data covers the 2007-2018 period. We therefore assign a '-1' to manufacturing and a '1' to other industries.
All other PV industries			1	See directly above
High-tech PV industries				
334412, 334413, 334417 334418, 334515, 335931	Bare circuit board manufacturing; Semiconductor and related device manufacturing; Electrical connector manufacturing; Printed circuit assembly; Instrument manufacturing for Measurement; Current-carrying device manufacturing;	9.5.1	1	'1' for all high- and medium-high R&D-intensity industries as classified in[22] for all countries. See SI section S-7.2 for details.
Medium-tech PV industries				
325211, 331313, 331314	Plastics material and resins manufacturing; Alumina refining; Alumina secondary smelting;	9.5.1	Country-dependent	'1' only for selected medium-R&D industrie for middle- and low-inc countries, since their average R&D spending lower than that of medium- R&D intensit industries. See SI sectio S-7.2 for details.
All PV industries				
		9.5.2		Same treatment as 9.5.1. See SI section S- for details.
All industries				
		9.A.1	1	Most industries receive official international support based on OEC data.[25] See SI section S-7.4 for details

Table S-38: **References for linkages between PV industries and SDG9 indicators in the local manufacturing scenario, Related to STAR Methods.** The same references are used in the import scenario, except that linkages between manufacturing industries and indicators are set to zero because technologies are assumed to be manufactured outside the boundary of the region for which SDG linkages are evaluated.

SDG9 (PART 4)	PV SYSTEMS			Local manufacturing
**NAICS Code**	**Industries**	**Indicator**	**Linkage**	**Explanation, References**
334412, 334413, 334417 334418, 334515, 335931	Bare circuit board manufacturing; Semiconductor and related device manufacturing; Electrical connector manufacturing; Printed circuit assembly; Instrument manufacturing for Measurement; Current-carrying device manufacturing;	9.B.1	1	The UN defines this indicator only for manufacturing industries, ('value added' refers to manufacturing value, using the classification of MHT industries by ISIC Rev. 3 and ISIC Rev. 4.[26] Based on this classification, medium- and high-tech industries include: Manufacture of computer, electronic and optical products; Manufacture of electrical equipment; Manufacture of chemicals and chemical products; Manufacture of machinery and equipment; Manufacture of electrical machinery and apparatus; Manufacture of radio, television and communication equipment and apparatus; We assign a '1' to the corresponding industries based on NAICS-codes.
All PV industries				
		9.C.1	0	No industry or service strictly necessitates a mobile network.

Table S-39: **References for linkages between PV industries and SDG9 indicators in the local manufacturing scenario, Related to STAR Methods.** The same references are used in the import scenario, except that linkages between manufacturing industries and indicators are set to zero because technologies are assumed to be manufactured outside the boundary of the region for which SDG linkages are evaluated.

SDG9 (PART 1)	WIND			Local manufacturing
**NAICS Code**	**Industries**	**Indicator**	**Linkage**	**Explanation, References**
Multiple wind industries				
212312, 212321	Crushed and Broken Limestone Mining and Quarrying; Construction Sand and Gravel Mining;	9.1.1	1	Same treatment as PV. Here we only list industries not also required for PV. Sand, limestone, and metal ore mines more broadly require access roads and road connections to railway stations and ports .[134,124,125,126,127]
212312, 212321, 325220 327320, 332111, 332312 332313, 332811, 333612 335311	Crushed and broken limestone mining and quarrying; Construction sand and gravel mining; Artificial and synthetic Fibers and filaments manufacturing; Ready mix Concrete manufacturing; Iron and steel forging; Fabricated structural metal manufacturing; Plate work manufacturing; Metal heat treating; Turbine and turbine generator set units manufacturing; Speed changer, industrial high-speed drive, and gear manufacturing; Power, distribution, and specialty transformer manufacturing	9.1.2	1	Same treatment as PV. Here we only list industries not also required for PV.
221115, 238110, 423320 541360	Wind electric power generation; Poured concrete foundation and structure contractors; Brick, stone, and related construction material merchant wholesalers; Geophysical surveying	9.1.2	0	Same treatment as for PV. Materials and component transportation is accounted for under the respective transportation services.
Manufacturing industries				
325220, 327320, 332111 332312, 332313, 332811 333611, 333612, 335311	Artificial and synthetic fibers and filaments manufacturing; Ready mix concrete manufacturing; Iron and steel forging; Fabricated structural metal manufacturing; Plate work manufacturing; Metal heat treating; Turbine and turbine generator set units manufacturing; Speed changer, industrial high-speed drive, and gear manufacturing; Power, distribution, and specialty transformer manufacturing	9.2.1<br>9.2.2	1<br>1	Same treatment as PV (see SI Table S-36). Here we only list the industries not also required for PV.
All other wind industries				
212312 , 212321, 238110	Crushed and broken limestone mining and quarrying; Construction sand and gravel mining; Poured concrete foundation and structure contractors	9.2.1<br>9.2.2	0<br>0	Same treatment as PV (see SI Table S-36). Here we only list the industries not also required for PV.

Table S-40: **References for linkages between wind industries and SDG9 indicators in the local manufacturing scenario, Related to STAR Methods.** The same references are used in the import scenario, except that linkages between manufacturing industries and indicators are set to zero because technologies are assumed to be manufactured outside the boundary of the region for which SDG linkages are evaluated.

SDG9 (PART 2)	WIND			Local manufacturing
**NAICS Code**	**Industries**	**Indicator**	**Linkage**	**Explanation, References**
Manufacturing industries				
212312, 212321, 325220 327320, 332111, 332312 332313, 332811, 333612 335311	Crushed and broken limestone mining and quarrying; Construction sand and gravel mining; Artificial and synthetic Fibers and filaments manufacturing; Ready mix Concrete manufacturing; Iron and steel forging; Fabricated structural metal manufacturing; Plate work manufacturing; Metal heat treating; Turbine and turbine generator set units manufacturing; Speed changer, industrial high-speed drive, and gear manufacturing; Power, distribution, and specialty transformer manufacturing	9.3.1<br>9.3.2	0<br>0	Same treatment as PV. (see SI section S-7 and SI Table S-37 for details). Here we only list industries not also required for PV.
Service industries				
238110, 423320, 541360	Poured concrete foundation and structure contractors; Brick, stone, and related construction material merchant wholesalers; Geophysical surveying	9.3.1<br>9.3.2	1<br>1	See explanation directly above.
Manufacturing industries				
325220, 327320, 332111 332312, 332313, 332811 333611, 333612, 335311	Artificial and synthetic fibers and filaments manufacturing; Ready mix concrete manufacturing; Iron and steel forging; Fabricated structural metal manufacturing; Plate work manufacturing; Metal heat treating; Turbine and turbine generator set units manufacturing; Speed changer, industrial high-speed drive, and gear manufacturing; Power, distribution, and specialty transformer manufacturing	9.4.1	-1	Same treatment as PV (see SI Table S-38). Here we only list the industries not also required for PV.
238110, 423320, 541360	Poured concrete foundation and structure contractors; Brick, stone, and related construction material merchant wholesalers; Geophysical contractors;	9.4.1	-1	Activity/service not associated with significant CO_2 emissions (we neglect transportation of personne to and from the site).

Table S-41: **References for linkages between wind industries and SDG9 indicators in the local manufacturing scenario, Related to STAR Methods.** The same references are used in the import scenario, except that linkages between manufacturing industries and indicators are set to zero because technologies are assumed to be manufactured outside the boundary of the region for which SDG linkages are evaluated.

SDG9 (PART 3)	WIND			Local manufacturing
**NAICS Code**	**Industries**	**Indicator**	**Linkage**	**Explanation, References**
High-tech wind industries				
333611, 333612, 335311	Turbine and turbine generator set units manufacturing; Speed changer, industrial high-speed drive, and gear manufacturing; Power, distribution, and specialty transformer ; manufacturing	9.5.1<br>9.5.2	1<br>1	Same treatment as PV (see SI Table S-38 and SI section S-7.2 for details). Here we list industries not also required for PV.
Medium-tech wind industries				
332111, 332312, 332313 332811	Iron and steel forging; Fabricated structural metal manufacturing; Plate work manufacturing; Metal heat treating	9.5.1<br><br>9.5.2	Country -dependent<br>Country -dependent	Same treatment as for PV industries (see references above).
All wind industries				
		9.A.1	1	Same treatment as PV.
Multiple wind industries				
		9.B.1	1<br>1	Same treatment as PV (see SI Table S-39 for details).
All wind industries				
		9.C.1	0	Same treatment as PV (see SI Table S-39).

Table S-42: **References for linkages between wind industries and SDG9 indicators in the local manufacturing scenario, Related to STAR Methods.** The same references are used in the import scenario, except that linkages between manufacturing industries and indicators are set to zero because technologies are assumed to be manufactured outside the boundary of the region for which SDG linkages are evaluated.

SDG9 (PART 1)	NUCLEAR			Local manufacturing
**NAICS Code**	**Industries**	**Indicator**	**Linkage**	**Explanation, References**
Multiple nuclear industries				
212291, 331210, 332410 332420, 332911, 333415 333912, 333914, 335312 335313, 423320	Uranium-radium-vanadium ore mining; Iron steel pipes and tubes manufacturing Power boiler/heat exchanger manufacturing Metal tank manufacturing; Industrial valve manufacturing; Air-conditioning and warm air heating equipment and commercial and industrial refrigeration equipment manufacturing; Air and gas compressor manufacturing; Measuring, dispensing, and other pumping equipment manufacturing; Motor and generator manufacturing; Switchgear and switchboard apparatus manufacturing; Brick, stone, and related construction material merchant wholesalers	9.1.1<br>9.1.2	1<br>1	Same treatment as for PV and wind. Here we list only industries not also required for PV and wind.<br><br>Same as above.
221113, 238120, 238220 561612	Nuclear electric power generation; Poured concrete foundation and structure contractors; Plumbing, heating, AC contractors; Security guard, patrol services	9.1.2	0	Same treatment as for PV. Materials and component transportation is accounted for under the respective transportation services.
Manufacturing industries				
212291, 325180, 331210 332410, 332420, 332911 333415, 333912, 333914 335312, 335313	Uranium-radium-vanadium ore mining; Other basic inorganic chemical manufacturing; Iron steel pipes and tubes manufacturing; Power boiler/heat exchanger manufacturing; Metal cask manufacturing; Industrial valve manufacturing; Air-conditioning and warm air heating equipment and commercial and industrial refrigeration equipment manufacturing; Air and gas compressor manufacturing; Measuring, dispensing, and other pumping equipment manufacturing; Motor and generator manufacturing; Switchgear and switchboard apparatus manufacturing;	9.2.1<br>9.2.2	1<br>1	Same treatment as for PV and wind. Here we list only industries not also required for PV and wind.<br><br>Same as above.
All other nuclear industries				
221113, 238120, 238220 423320, 561612	Nuclear electric power generation; Structural steel and precast concrete contractors; Plumbing, heating, AC contractors; Brick, stone, and related construction material merchant wholesalers; Security guard, patrol services;	9.2.1<br>9.2.2	0<br>0	Same treatment as PV (see SI Table S-36). Here we only list the industries not also required for PV.

Table S-43: **References for linkages between wind industries and SDG9 indicators in the local manufacturing scenario, Related to STAR Methods.** The same references are used in the import scenario, except that linkages between manufacturing industries and indicators are set to zero because technologies are assumed to be manufactured outside the boundary of the region for which SDG linkages are evaluated.

SDG9 (PART 2)	NUCLEAR			Local manufacturing
**NAICS Code**	**Industries**	**Indicator**	**Linkage**	**Explanation, References**
Manufacturing industries				
212291, 325180, 331210 332410, 332420, 332911 333415, 333912, 333914 335312, 335313, 423320	Uranium-radium-vanadium ore mining; Other basic inorganic chemical manufacturing; Iron steel pipes and tubes manufacturing; Power boiler/heat exchanger manufacturing; Metal cask manufacturing; Industrial valve manufacturing; Air-conditioning and warm air heating equipment and commercial and industrial refrigeration equipment manufacturing; Air and gas compressor manufacturing; Measuring, dispensing, and other pumping equipment manufacturing; Motor and generator manufacturing; Switchgear and switchboard apparatus manufacturing;	9.3.1<br>9.3.2	0<br>0	Same treatment as for PV and wind (see SI section S-7). Here we list only industries not also required for PV and wind. Same as above.
Service industries				
221113, 238120, 238220 423320, 561612	Nuclear electric power generation; Structural steel and precast concrete contractors; Plumbing, heating, AC contractors; Brick, stone, and related construction material merchant wholesalers; Security guard, patrol services;	9.3.1<br>9.3.2	1<br>1	Same treatment as PV (see SI Table S-36). Here we only list the industries not also required for PV.
Manufacturing industries				
325180, 331210, 332410 332420, 332911, 333415 333912, 333914, 335312 335313, 423320	Other basic inorganic chemical manufacturing; Iron steel pipes and tubes manufacturing; Power boiler/heat exchanger manufacturing; Metal cask manufacturing; Industrial valve manufacturing; Air-conditioning and warm air heating equipment and commercial and industrial refrigeration equipment manufacturing; Air and gas compressor manufacturing; Measuring, dispensing, and other pumping equipment manufacturing; Motor and generator manufacturing; Switchgear and switchboard apparatus manufacturing;	9.4.1	-1	Same treatment as for PV and wind (see SI Table S-38). Here we list only industries not also required for PV and wind. Same as above.
All other nuclear industries				
		9.4.1	1	Same treatment as PV (see SI Table S-38).

Table S-44: **References for linkages between nuclear fission plant industries and SDG9 indicators in the local manufacturing scenario, Related to STAR Methods.** The same references are used in the import scenario, except that linkages between manufacturing industries and indicators are set to zero because technologies are assumed to be manufactured outside the boundary of the region for which SDG linkages are evaluated.

SDG9 (PART 3)	NUCLEAR			Local manufacturing
**NAICS Code**	**Industries**	**Indicator**	**Linkage**	**Explanation, References**
High-tech nuclear industries				
325180, 332410, 332420 332911, 333415, 333912 333914, 335312, 335313 423320	Other basic inorganic chemical manufacturing; Power boiler/heat exchanger manufacturing; Metal cask manufacturing; Industrial valve manufacturing; Air-conditioning and warm air heating equipment and commercial and industrial refrigeration equipment manufacturing; Air and gas compressor manufacturing; Measuring, dispensing, and other pumping equipment manufacturing; Motor and generator manufacturing; Switchgear and switchboard apparatus manufacturing;	9.5.1<br>9.5.2<br>9.B.1	1<br>1<br>1	Same treatment as for PV and wind (see SI Table S-7). Here we list only industries not also required for PV and wind. See SDG9 PV Table 4 for 9.B.1
All nuclear industries				
		9.A.1<br>9.C.1	1<br>0	Same treatment as PV.

Table S-45: **References for linkages between nuclear fission plant industries and SDG9 indicators in the local manufacturing scenario, Related to STAR Methods.** The same references are used in the import scenario, except that linkages between manufacturing industries and indicators are set to zero because technologies are assumed to be manufactured outside the boundary of the region for which SDG linkages are evaluated.

SDG9 (PART 1)	COOKSTOVES			Local manufacturing
**NAICS Code**	**Industries**	**Indicator**	**Linkage**	**Explanation, References**
Mining industries				
212325, 212393	Clay and ceramic and refractory minerals mining; Other chemical and fertilizer mineral mining	9.1.1	1	Same treatment as PV and wind. Here we only list cookstove-specific industries.
Multiple industries				
212325, 212393, 327120 332215, 335912	Clay and ceramic and refractory minerals mining; Clay building material and refractories manufacturing (includes ceramic tiles manufacturing, fire brick manufacturing, and liner brick manufacturing) Metal kitchen cookware, utensil, cutlery, and flatware (except precious) manufacturing; Primary battery manufacturing;	9.1.2	1	Same treatment as PV and wind.
Agriculture				
113210, 115112	Forest nurseries and gathering of forest products; Soil preparation, planting, and cultivating	9.1.2	0	No clear link (fuel wood wood or other solid fuel types are typically collected by hand directly by the consumer, not requiring transport by air or truck (e.g.,.[135])
423620	Household appliances, electric housewares, and consumer electronics merchant x wholesalers	9.3.1<br>9.3.2	1<br>1	Same treatment as as for PV and wind. Here we list only industries not also used by PV and wind.
Manufacturing industries				
	Clay building material and refractories manufacturing (includes ceramic tiles manufacturing, fire brick manufacturing, and liner brick manufacturing) Metal kitchen cookware, utensil, cutlery, and flatware (except precious) manufacturing; Primary battery manufacturing;	9.2.1<br>9.2.2	1<br>1	Same treatment as PV and wind.

Table S-46: **References for linkages between clean cookstove industries and SDG9 indicators in the local manufacturing scenario, Related to STAR Methods.** The same references are used in the import scenario, except that linkages between manufacturing industries and indicators are set to zero because technologies are assumed to be manufactured outside the boundary of the region for which SDG linkages are evaluated.

SDG9 (PART 2)	COOKSTOVES			Local manufacturing
**NAICS Code**	**Industries**	**Indicator**	**Linkage**	**Explanation, References**
212325, 212393	Clay and ceramic and refractory minerals mining Other chemical and fertilizer mineral mining	9.4.1	-1	CO_2 emissions arise from fuel use during mining as well as from clay dryers and calciners.[100]
327130	Clay building material and refractories manufacturing	9.4.1	-1	
332215	Metal kitchen cookware	9.4.1	-1	Iron/steel industries are energy intensive and contribute substantially to industrial CO_2 emissions (e.g.,[136,137])
335912		9.4.1	-1	Battery manufacturing emits 6-18 kg CO2 per kg of battery for Li-Ion.[138]
113210, 115112	Forest nurseries and gathering of forest products; Soil preparation, planting, and cultivating	9.4.1	-1	Country-dependent but fuel wood production can contribute to forest loss and thereby to indirect CO_2 emissions from land use changes.
335912	Primary battery manufacturing	9.5.1<br>9.5.2<br>9.B.1	1<br>1<br>1	Same treatment as PV. Electrical equipment is a medium-high R&D intensity industry.[22]
All cookstove industries				
		9.A.1<br>9.C.1	1<br>0	Same treatment as PV

Table S-47: **References for linkages between clean cookstove industries and SDG9 indicators in the local manufacturing scenario, Related to STAR Methods.** The same references are used in the import scenario, except that linkages between manufacturing industries and indicators are set to zero because technologies are assumed to be manufactured outside the boundary of the region for which SDG linkages are evaluated.

References

[1] *Renewable Energy and Jobs - Annual Review 2020. (2020).* Tech. rep. International Renewable Energy Agency.

[2] Cameron L and Van Der Zwaan B. (2015). "Employment factors for wind and solar energy technologies: A literature review". In: *Renewable and Sustainable Energy Reviews* 45 (), pp. 160–172.

[3] UN (2021). *SDG indicator metadata: Indicator information for Indicator 9.b.1.* Tech. rep. United Nations. URL: https://unstats.un.org/sdgs/metadata/files/Metadata-09-0B-01.pdf.

[4] Kanagawa M. and Nakata T. (2008). "Assessment of access to electricity and the socio-economic impacts in rural areas of developing countries". In: *Energy policy* 36.6 (2008), pp. 2016–2029.

[5] Willcox M. et al. (2015). "Utilising Electricity Access for Poverty Reduction: ANNEX 2 - CASE STUDY REPORT: KENYA". In: *Study Report, Practical Action Consulting* (2015).

[6] Lenz L. et al. (2017). "Does large-scale infrastructure investment alleviate poverty? Impacts of Rwandaâs electricity access roll-out program". In: *World Development* 89 (2017), pp. 88–110.

[7] Muhammad Sajjad (2021). "Disaster resilience in Pakistan: a comprehensive multi-dimensional spatial profiling". In: *Applied geography* 126 (), p. 102367.

[8] Cutter S. (2016). "The landscape of disaster resilience indicators in the USA". In: *Natural hazards* 80.2 (), pp. 741–758.

[9] Hertwich E. et al. (2015). "Integrated life-cycle assessment of electricity-supply scenarios confirms global environmental benefit of low-carbon technologies". In: *Proceedings of the National Academy of Sciences* 112.20 (), pp. 6277–6282.

[10] EPA (2017). *2017 National Emissions Inventory (NEI) Data.* Tech. rep. United States Environmental Protection Agency. URL: https://www.epa.gov/air-emissions-inventories/2017-national-emissions-inventory-nei-data.

[11] Sohail Ahmad (2014)., Manu V Mathai, and Govindan Parayil. "Household electricity access, availability and human well-being: Evidence from India". In: *Energy Policy* 69 (), pp. 308–315.

[12] Winkler H. et al. (2011). "Access and affordability of electricity in developing countries". In: *World development* 39.6 (), pp. 1037–1050.

[13] ILO. *Women in managerial and leadership positions in the G20.* Tech. rep. International Labor Office. URL: https://www.ilo.org/wcmsp5/groups/public/---dgreports/---ddg_p/documents/publication/wcms_762098.pdf.

[14] GEMI (2019). *Step-by-step monitoring methodology for SDG indicate 6.4.1.* Tech. rep. Integrated Monitoring Initiative for SDG 6.

[15] *United Nations Global SDG Database.* https://unstats.un.org/sdgs/indicators/database/. Accessed: 2021-07-29.

[16] Meldrum J. et al. (2013). "Life cycle water use for electricity generation: a review and harmonization of literature estimates". In: *Environmental Research Letters* 8.1 (), p. 015031.

[17] *Global average LCOEs and auction results for utility-scale PV by commissioning date.* https://www.iea.org/data-and-statistics/charts/global-average-lcoes-and-auction-results-for-utility-scale-pv-by-commissioning-date.

[18] *Actual and forecast onshore wind costs, 2016-2025.* https://www.iea.org/data-and-statistics/charts/actual-and-forecast-onshore-wind-costs-2016-2025.

[19] Lazard (2020). *Lazard's Levelized Cost of Electricity Analysis - Version 14.0.* Tech. rep.

[20] OECD Publishing. *Entrepreneurship at a Glance 2017.* Tech. rep. OECD, 2017.

[21] *SMME Quarterly Update: 1st Quarter 2019.* http://www.seda.org.za/Publications/Publications/SMME%20Quarterly%202019-Q1.pdf.

[22] Galindo-Rueda F. and Verger. F. (2016). "OECD taxonomy of economic activities based on R&D intensity". In: ().

[23] *Research and development expenditure (% of GDP).* https://data.worldbank.org/indicator/GB.XPD.RSDV.GD.ZS.

[24] *Researchers per million inhabitants.* https://en.unesco.org/sites/default/files/researchers_per_million_inhabitants_fte_1996-2018.pdf.

[25] *SDG Pulse 2021: Official international assistance plays a key role in financing for sustainable development.* https://sdgpulse.unctad.org/official-support-development/#Ref_Oecd2020b.

[26] *United Nations Metadata Repository, Indicator 9.B.1.* https://unstats.un.org/sdgs/metadata/files/Metadata-09-0B-01.pdf.

[27] Nicolau R. et al. (2019). "Ratio of land consumption rate to population growth rateâAnalysis of different formulations applied to mainland Portugal". In: *ISPRS International Journal of Geo-Information* 8.1 (), p. 10.

[28] Ostro B. (1993). "The association of air pollution and mortality: examining the case for inference". In: *Archives of Environmental Health: An International Journal* 48.5 (), pp. 336–342.

[29] Yin P. et al (2020). "The effect of air pollution on deaths, disease burden, and life expectancy across China and its provinces, 1990–2017: an analysis for the Global Burden of Disease Study 2017". In: *The Lancet Planetary Health* 4.9 (), e386–e398.

[30] Balakrishnan K. et al. (2019). "The impact of air pollution on deaths, disease burden, and life expectancy across the states of India: the Global Burden of Disease Study 2017". In: *The Lancet Planetary Health* 3.1 (), e26–e39.

[31] *Trade in environmentally sound technologies: Implications for Developing Countries.* Tech. rep. United Nations Environment Programme (2018). URL: https://wedocs.unep.org/bitstream/handle/20.500.11822/27595/TradeEnvTech.pdf?sequence=1&isAllowed=y.

[32] *List of waste referred to in Article 7 of directive 2008/98/EC.* Tech. rep. Chen, Wei-Qiang (2008). URL: https://eur-lex.europa.eu/legal-content/EN/TXT/?uri=CELEX:02000D0532-20150601.

[33] *National Overview: Facts and Figures on Materials, Wastes and Recycling.* Tech. rep. URL: https://www.epa.gov/facts-and-figures-about-materials-waste-and-recycling/national-overview-facts-and-figures-materials#recycling.

[34] *Municipal solid waste statistics.* Tech. rep. Eurostat (2022). URL: https://ec.europa.eu/eurostat/statistics-explained/index.php?title=Municipal_waste_statistics.

[35] Graedel T. et al. (2011). "What do we know about metal recycling rates?" In: *Journal of Industrial Ecology* 15.3 (), pp. 355–366.

[36] Chen WQ (2013). "Recycling rates of aluminum in the United States". In: *Journal of Industrial Ecology* 17.6 (), pp. 926–938.

[37] Intergovernmental Oceanographic Commission and others (2005). "IOC criteria and guidelines on the transfer of marine technology." In: ().

[38] Van de Ven D.-J. et al. (2021). "The potential land requirements and related land use change emissions of solar energy". In: *Scientific reports* 11.1 (2021), pp. 1–12.

[39] Denholm P. et al. (2009). *Land use requirements of modern wind power plants in the United States.* Tech. rep. National Renewable Energy Lab.(NREL), Golden, CO (United States).

[40] Fthenakis V. and Kim HC (2009). "Land use and electricity generation: A life-cycle analysis". In: *Renewable and Sustainable Energy Reviews* 13.6-7 (), pp. 1465–1474.

[41] Worlanyo A.S. and Jiangfeng L. (2021). "Evaluating the environmental and economic impact of mining for post-mined land restoration and land-use: A review". In: *Journal of Environmental Management* 279 (2021), p. 111623.

[42] UNECE (2022). *Indicator 15.3.1: Proportion of land that is degraded over total land area.* Tech. rep. United Nations Economic Commission for Europe. URL: https://w3.unece.org/SDG/en/Indicator?id=66.

[43] *EARN03: Average weekly earnings by industry.* Tech. rep. United Kingdom Office for National Statistics.

[44] L Kruitwagen (2021). et al. "A global inventory of photovoltaic solar energy generating units". In: *Nature* 598.7882 (), pp. 604–610.

[45] Mingquan Li (2022). et al. "High-resolution data shows Chinaâs wind and solar energy resources are enough to support a 2050 decarbonized electricity system". In: *Applied Energy* 306 (), p. 117996.

[46] *Private Participation in Infrastructure Database.* Tech. rep. The World Bank (2022). URL: https://ppi.worldbank.org/en/customquery.

[47] *Public Private Partnership Legal Resource Center.* Tech. rep. The World Bank (2022). URL: https://ppp.worldbank.org/public-private-partnership/sector.

[48] UN Economic and Social (2016). Council. "Report of the inter-agency and expert group on sustainable development goal indicators". In: *Stat. Comm* 13 ().

[49] *End of Life Management Solar Photovoltaic Panels.* Tech. rep. International Renewable Energy Agency (2016). URL: https://www.irena.org/-/media/Files/IRENA/Agency/Publication/2016/IRENA_IEAPVPS_End-of-Life_Solar_PV_Panels_2016.pdf.

[50] Majewski P. et al. (2021). "Recycling of solar PV panels-product stewardship and regulatory approaches". In: *Energy Policy* 149 (), p. 112062.

[51] Dodd N. et al (2020). *Preparatory study for solar photovoltaic modules, inverters and systems, EUR 30468 EN.* Tech. rep. Publications Office of the European Union. URL: https://susproc.jrc.ec.europa.eu/product-bureau/sites/default/files/2020-12/jrc12431preparatory_study_for_solar_photovoltaic_modules_kj-na-30468-en.pdf.

[52] Razdan P. and Garrett P. (2019). *Life Cycle Assessment of Electricity Production from an Onshore V150-4.2 MW Plant.* Tech. rep. Vestas Wind System. URL: https://www.vestas.com/content/dam/vestas-com/global/en/sustainability/reports-and-ratings/lcas/LCA%5C%20of%5C%20Electricity%5C%20Production%5C%20from%5C%20an%5C%20onshore%5C%20V15042MW%5C%20Wind%5C%20PlantFinal.pdf.coredownload.inline.pdf.

[53] Deeney P. et al. (2021). "End-of-Life alternatives for wind turbine blades: Sustainability Indices based on the UN sustainable development goals". In: *Resources, Conservation and Recycling* 171 (), p. 105642.

[54] UNECE (2021). *Life Cycle Assessment of Electricity Generation Options.* Tech. rep. United Nations Economic Commission for Europe. URL: https://unece.org/sites/default/files/2021-10/LCA-2.pdf.

[55] *Managing low radioactivity material from the demolition of nuclear facilities.* Tech. rep. International Atomic Energy Agency (2008). URL: https://www-pub.iaea.org/MTCD/publications/PDF/trs462_web.pdf.

[56] *Radioactive Waste Management.* Tech. rep. World Nuclear Association (2022). URL: https://world-nuclear.org/information-library/nuclear-fuel-cycle/nuclear-wastes/radioactive-waste-management.aspx.

[57] *ACE Ultra-Clean Biomass Cookstove User Manual.* Tech. rep. African Clean Energy (ACE) (2021). URL: http://www.thestoveauction.org/uploads/2/4/8/5/24859908/1505_ace_1_instruction_manual.pdf.

[58] Zhu L. and Chen M. (2020). "Research on Spent LiFePO4 Electric Vehicle Battery Disposal and Its Life Cycle Inventory Collection in China". In: *International Journal of Environmental Research and Public Health* 17.23 (), p. 8828.

[59] A Samecka-Cymerman (2004). and AJ Kempers. "Toxic metals in aquatic plants surviving in surface water polluted by copper mining industry". In: *Ecotoxicology and Environmental Safety* 59.1 (), pp. 64–69.

[60] Ek A.S. and Renberg I. (2001). "Heavy metal pollution and lake acidity changes caused by one thousand years of copper mining at Falun, central Sweden". In: *Journal of paleolimnology* 26.1 (), pp. 89–107.

[61] Dudka S. and Adriano DC (1997). "Environmental impacts of metal ore mining and processing: a review". In: *Journal of environmental quality* 26.3 (), pp. 590–602.

[62] Northey S., Haque N., and Mudd G. (2013). "Using sustainability reporting to assess the environmental footprint of copper mining". In: *Journal of Cleaner Production* 40 (), pp. 118–128.

[63] Houston J. (2002). "Groundwater recharge through an alluvial fan in the Atacama Desert, northern Chile: mechanisms, magnitudes and causes". In: *Hydrological processes* 16.15 (), pp. 3019–3035.

[64] Soropogui M. et al. (2019). "Bauxite mining and refinery: investigating prospective red mud management strategies". In: *J. Eng. Sci. Technol.* 14 (), pp. 120–134.

[65] Farjana S. et al. "Impacts of aluminum production: A cradle to gate investigation using life-cycle assessment". In: *Science of The Total Environment* 663 (), pp. 958–970.

[66] M Naveen Saviour (2012). "Environmental impact of soil and sand mining: a review". In: *International Journal of Science, Environment and Technology* 1.3 (), pp. 125–134.

[67] Torres A. et al. "A looming tragedy of the sand commons". In: *Science* 357.6355 (), pp. 970–971.

[68] Anthony E. et al. (2015). "Linking rapid erosion of the Mekong River delta to human activities". In: *Scientific reports* 5.1 (2015), pp. 1–12.

[69] Pereira K. and Ratnayake R. (2013). "Water integrity in action: Curbing illegal sand mining in Sri Lanka". In: *Berlin, Germany: Water Integrity Network* ().

[70] Macknick J. and Cohen S. (2015). *Water impacts of high solar PV electricity penetration.* Tech. rep. National Renewable Energy Lab.(NREL), Golden, CO (United States).

[71] Zhenkun Lin (2017). et al. "A study on environmental bisphenol a pollution in plastics industry areas". In: *Water, Air, & Soil Pollution* 228.3 (), p. 98.

[72] Werner Vogel (2012). *Glass chemistry.* Springer Science & Business Media.

[73] Gholipour A., Zahabi H., and Stefanakis A. (2020). "A novel pilot and full-scale constructed wetland study for glass industry wastewater treatment". In: *Chemosphere* 247 (), p. 125966.

[74] Varun M. et al. "Metal contamination of soils and plants associated with the glass industry in North Central India: prospects of phytoremediation". In: *Environmental Science and Pollution Research* 19.1 (), pp. 269–281.

[75] Ozkan E., Elginoz N., and Babuna F.G. (2018). "Life cycle assessment of a printed circuit board manufacturing plant in Turkey". In: *Environmental Science and Pollution Research* 25.27 (), pp. 26801–26808.

[76] Frost K. and Hua I. (2019). "Quantifying spatiotemporal impacts of the interaction of water scarcity and water use by the global semiconductor manufacturing industry". In: *Water Resources and Industry* 22 (), p. 100115.

[77] Rao P., Sholes D., and Cresko J. (2019). "Evaluation of US manufacturing subsectors at risk of physical water shortages". In: *Environmental science & technology* 53.5 (), pp. 2295–2303.

[78] Ghinwa M Naja (2011). et al. “Hydrochemical impacts of limestone rock mining”. In: *Water, Air, & Soil Pollution* 217.1 (), pp. 95–104.

[79] Iwanoff A. (1998). “Environmental impacts of deep opencast limestone mines in Laegerdorf, Northern Germany”. In: *Mine Water and the Environment* 17.1 (), pp. 52–61.

[80] RE Lamare (2016). and OP Singh. “Limestone mining and its environmental implications in Meghalaya, India”. In: *ENVIS Bulletin Himalayan Ecology* 24 (), pp. 87–100.

[81] O.P. Singh (2019). *Study on Mining Affected Areas and its Impact on Livelihood.* Tech. rep. Meghalaya Basin Management Agency.

[82] Das S. (2011). “Life cycle assessment of carbon fiber-reinforced polymer composites”. In: *The International Journal of Life Cycle Assessment* 16.3 (), pp. 268–282.

[83] Sunter D. et al. (2015). “The manufacturing energy intensity of carbon fiber reinforced polymer composites and its effect on life cycle energy use for vehicle door lightweighting”. In: *Proceedings of the 20th International Conference on Composite Materials (ICCM), Copenhagen, Denmark.*

[84] Javad Dawoudian (2021)., Sadegh Bahamin, and Henry Bikwibili Tantoh. “Environmental impact assessment of cement industries using mathematical matrix method: case of Ghayen cement, South Khorasan, Iran”. In: *Environmental Science and Pollution Research* 28.18 (), pp. 22348–22358.

[85] Wenqiang Sun (2019). et al. “Environmental impact assessment of wastewater discharge with multi-pollutants from iron and steel industry”. In: *Journal of environmental management* 245 (), pp. 210–215.

[86] Chunyan Wang (2017). et al. “A technology-based analysis of the water-energy-emission nexus of Chinaâs steel industry”. In: *Resources, Conservation and Recycling* 124 (), pp. 116–128.

[87] Sachidananda M., Webb D. P., and Shahin S. (2016). Rahimifard. “A concept of water usage efficiency to support water reduction in manufacturing industry”. In: *Sustainability* 8.12 (), p. 1222.

[88] Zheng B. et al (2020). “Dynamic wastewater-induced research based on input-output analysis for Guangdong Province, China”. In: *Environmental Pollution* 256 (), p. 113502.

[89] Hunziker (2020). C. et al. “Sustainability assessment of novel transformer technologies in distribution grid applications”. In: *Sustainable Energy, Grids and Networks* 21 (), p. 100314.

[90] *Monitoring methodology for SDG indicator 6.6.1.* `https://wesr.unep.org/media/docs/projects/6_6_1_methodology_final.pdf`.

[91] Kaufmann R. F., Eadie G.G., and Russell C.R. (1976). “Effects of uranium mining and milling on ground water in the Grants Mineral Belt, New Mexico”. In: *Groundwater* 14.5 (), pp. 296–308.

[92] Jin Wang (2012). et al. “Surface water contamination by uranium mining/milling activities in Northern Guangdong Province, China”. In: *CLEAN–Soil, Air, Water* 40.12 (), pp. 1357–1363.

[93] Mielke E., Anadon L.D., and Venkatesh V. (2010). Narayanamurti. “Water consumption of energy resource extraction, processing, and conversion”. In: *Belfer Center for Science and International Affairs* ().

[94] IAEA. *Environmental aspects based on operational performance of nuclear fuel fabrication facilities.* Tech. rep. International Atomic Energy Agency, 2002.

[95] CW Francis (2013). and CD Malone. “Anaerobic columnar denitrification of high nitrate wastewater”. In: *Proceedings of the Conference on Nitrogen As a Water Pollutant.* Elsevier, pp. 687–711.

[96] *Nuclear Fuel and its Fabrication.* Tech. rep. World Nuclear Association (2021). URL: `https://world-nuclear.org/information-library/nuclear-fuel-cycle/conversion-enrichment-and-fabrication/fuel-fabrication.aspx`.

[97] Yifan Gu (2015). et al. “Calculation of water footprint of the iron and steel industry: a case study in Eastern China”. In: *Journal of Cleaner Production* 92 (), pp. 274–281.

[98] Mello R. et al. (2016). “Using the mineralogy of river sediments as pollution indicator of clay mining activity”. In: *RBRH* 21 (), pp. 502–513.

[99] Manoj B, Vineethkumar V., and Prakash V. (2020). “Drinking water quality assessment in the water around a clay mine in Kannur district, Kerala”. In: *Radiation Protection and Environment* 43.2 (), p. 88.

[100] Mukherjee S. (2013). “Environmental impacts of clay-related industries”. In: *The Science of Clays.* Springer, pp. 280–295.

[101] V Santhosh (2013). et al. “Brick and tile clay mining from the paddy lands of Central Kerala (southwest coast of India) and emerging environmental issues”. In: *Environmental earth sciences* 68.7 (), pp. 2111–2121.

[102] Ranasinghe M. (1997). “Reconciling private profitability and social costs: the case of clay mining in Sri Lanka”. In: *Project Appraisal* 12.1 (), pp. 31–41.

[103] Bolan N. et al. (2021). “From mine to mind and mobiles–Lithium contamination and its risk management”. In: *Environmental Pollution* 290 (), p. 118067.

[104] Agusdinata W. (2020.) Liu (2020).and D.B. “Interdependencies of lithium mining and communities sustainability in Salar de Atacama, Chile”. In: *Journal of Cleaner Production* 260 (), p. 120838.

[105] Boschi G. et al. (2020). “Sustainability in Italian Ceramic Tile Production: Evaluation of the Environmental Impact”. In: *Applied Sciences* 10.24 (), p. 9063.

[106] Mezquita A. et al (2017). “How to reduce energy and water consumption in the preparation of raw materials for ceramic tile manufacturing: Dry versus wet route”. In: *Journal of Cleaner Production* 168 (), pp. 1566–1570.

[107] B Lattimore (2009). et al. “Environmental factors in woodfuel production: Opportunities, risks, and criteria and indicators for sustainable practices”. In: *Biomass and bioenergy* 33.10 (), pp. 1321–1342.

[108] Miao Wang (2009). “Manufacturing FDI and economic growth: evidence from Asian economies”. In: *Applied Economics* 41.8 (), pp. 991–1002.

[109] Szirmai A. and Verspagen B. (2015). “Manufacturing and economic growth in developing countries, 1950–2005”. In: *Structural change and economic dynamics* 34 (), pp. 46–59.

[110] Eichengreen B. and Gupta P. (2013). “The two waves of service-sector growth”. In: *Oxford Economic Papers* 65.1 (), pp. 96–123.

[111] *Powering Jobs Census 2019: The Energy Access Workforce.* Tech. rep. Power for All (2019). URL: `https://www.powerforall.org/application/files/8915/6310/7906/Powering-Jobs-Census-2019.pdf`.

[112] *SDG indicator metadata: Harmonized metadata template - format version 1.0.* Tech. rep. United Nations (2021). URL: `https://unstats.un.org/sdgs/metadata/files/Metadata-08-04-01.pdf`.

[113] Muro M. et al. (2019). *Advancing inclusion through clean energy jobs.* Tech. rep. Brookings Metropolitan Policy Program. URL: `https://www.brookings.edu/wp-content/uploads/2019/04/2019.04_metro_Clean-Energy-Jobs_Report_Muro-Tomer-Shivaran-Kane_updated.pdf`.

[114] *Ending child labour, forced labour and human trafficking in global supply chains.* Tech. rep. OECD (2019). URL: http://mneguidelines.oecd.org/Ending-child-labour-forced-labour-and-human-trafficking-in-global-supply-chains.pdf.

[115] *Most Dangerous Industries: Industry Incidence and Rates.* Tech. rep. National Safety Council. URL: https://injuryfacts.nsc.org/work/industry-incidence-rates/most-dangerous-industries/.

[116] FC Prinsloo (2013). "Impact of renewable energy structures on tourism". In: *Stellenbosch University* ().

[117] Kuldeep N., Chawla K., and Ghosh A. (2017). *Greening India's Workforce: Gearing up for Expansion of Solar and Wind Power in India.* Tech. rep. Natural Resources Defense Council. URL: https://www.powerforall.org/application/files/8915/6310/7906/Powering-Jobs-Census-2019.pdf.

[118] Nuclear Energy Agency and International Atomic Energy Agency. *Measuring Employment Generated by the Nuclear Power Sector*, p. 96. DOI: https://doi.org/https://doi.org/10.1787/9789264305960-en. URL: https://www.oecd-ilibrary.org/content/publication/9789264305960-en.

[119] *Wages, productivity and labour share in China: Research Note.* https://www.ilo.org/wcmsp5/groups/public/---asia/---ro-bangkok/documents/publication/wcms_475254.pdf.

[120] *Wages.* https://www.bls.gov/oes/current/oes452099.htm.

[121] Shirley R. et al. (2020). "Powering Jobs: the Employment Footprint of Clean Cooking Solutions in Kenya". In: ().

[122] Hernandez T. and Gabbard S. (2020). *Findings from the National Agricultural Workers Survey (NAWS) 2015-2016: A Demographic and Employment Profile of United States Farmworkers.* Tech. rep. JBS International. URL: https://www.dol.gov/sites/dolgov/files/ETA/naws/pdfs/NAWS_Research_Report_13.pdf.

[123] S Subramanian (2015). et al. *Emerging trends and patterns of India's agricultural workforce: Evidence from the census.* Institute for Social and Economic Change Bangalore, India.

[124] Mngeni Asabonga (2017). et al. "The physical and environmental impacts of sand mining". In: *Transactions of the Royal society of South Africa* 72.1 (), pp. 1–5.

[125] Johansson T. (2003). *Jamaican Deforestation and Bauxite Mining-Applying the Coase theorem.*

[126] Ric Careless (1993). and Lisa E Barnese. "The Tatshenshini Wilderness: under threat of mining". In: *Journal of the North American Benthological Society* 12.2 (), pp. 211–214.

[127] J. Schulman (2015). *Arizona State Freight Plan: Phase 3 Working Paper: Mining Sector Profile and Transportation Performance Needs.* Tech. rep. Arizona Department of Transportation.

[128] Blachowski J. (2014). "Spatial analysis of the mining and transport of rock minerals (aggregates) in the context of regional development". In: *Environmental earth sciences* 71.3 (), pp. 1327–1338.

[129] *International Standard Industrial Classification of All Economic Activities, Revision 4.* https://unstats.un.org/unsd/publication/seriesm/seriesm_4rev4e.pdf.

[130] *SDG Indicator Metadata: Goal 9.* https://unstats.un.org/sdgs/metadata/?Text=&Goal=9&Target=9.2.

[131] Kumar K.B., Rajan R.G., and Zingales L. (1999). *What determines firm size?* Tech. rep. National bureau of economic research.

[132] *Carbon dioxide emissions per unit of GDP, kg of CO2 per constant 2010 USD.* https://w3.unece.org/SDG/en/Indicator?id=28.

[133] *Carbon dioxide emissions per unit of manufacturing value added, kg of CO2 per constant 2010 USD.* https://w3.unece.org/SDG/en/Indicator?id=29.

[134] Joseph AH Oates (2008). *Lime and limestone: chemistry and technology, production and uses.* John Wiley & Sons.

[135] Das K., Pradhan G., and Nonhebel S. (2019). "Human energy and time spent by women using cooking energy systems: A case study of Nepal". In: *Energy* 182 (), pp. 493–501.

[136] Nicolás Pardo (2013). and José Antonio Moya. "Prospective scenarios on energy efficiency and CO2 emissions in the European Iron & Steel industry". In: *Energy* 54 (), pp. 113–128.

[137] Wenqing Xu (2016). et al. "CO2 emissions from China's iron and steel industry". In: *Journal of Cleaner Production* 139 (), pp. 1504–1511.

[138] JL Sullivan (2010)., Leigh Gaines, et al. "A review of battery life-cycle analysis: state of knowledge and critical needs." In: ().
